# Light-Driven Hybrid Nanoreactor Harnessing the Synergy of Carboxysomes and Organic Frameworks for Efficient Hydrogen Production

**DOI:** 10.1021/acscatal.4c03672

**Published:** 2024-12-06

**Authors:** Jing Yang, Qiuyao Jiang, Yu Chen, Quan Wen, Xingwu Ge, Qiang Zhu, Wei Zhao, Oluwatobi Adegbite, Haofan Yang, Liang Luo, Hang Qu, Veronica Del-Angel-Hernandez, Rob Clowes, Jun Gao, Marc A. Little, Andrew I. Cooper, Lu-Ning Liu

**Affiliations:** † Materials Innovation Factory and Department of Chemistry, University of Liverpool, Liverpool L7 3NY, U.K.; ‡ Institute of Systems, Molecular and Integrative Biology, University of Liverpool, Liverpool L69 7ZB, U.K.; § Hubei Key Laboratory of Agricultural Bioinformatics, College of Informatics, Huazhong Agricultural University, Wuhan 430070, China; ∥ College of Marine Life Sciences and Frontiers Science Center for Deep Ocean Multispheres and Earth System, Ocean University of China, Qingdao 266003, China; ⊥ Institute of Chemical Sciences, Heriot-Watt University, Edinburgh EH14 4AS, U.K.

**Keywords:** light-driven, nanoreactor, carboxysomes, organic frameworks, hybrid catalyst, hydrogen production

## Abstract

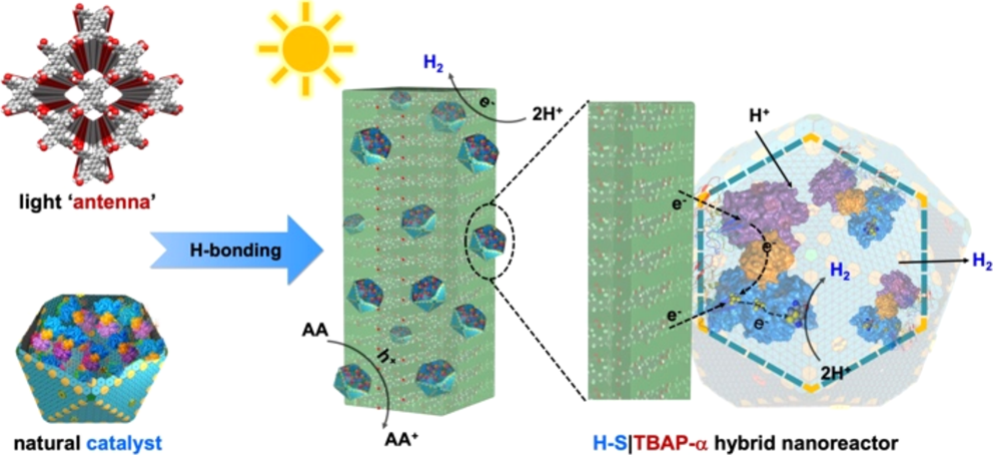

Synthetic photobiocatalysts are promising catalysts for valuable chemical transformations by harnessing solar energy inspired by natural photosynthesis. However, the synergistic integration of all of the components for efficient light harvesting, cascade electron transfer, and efficient biocatalytic reactions presents a formidable challenge. In particular, replicating intricate multiscale hierarchical assembly and functional segregation involved in natural photosystems, such as photosystems I and II, remains particularly demanding within artificial structures. Here, we report the bottom-up construction of a visible-light-driven chemical–biological hybrid nanoreactor with augmented photocatalytic efficiency by anchoring an α-carboxysome shell encasing [FeFe]-hydrogenases (H–S) on the surface of a hydrogen-bonded organic molecular crystal, a microporous α-polymorph of 1,3,6,8-tetra(4′-carboxyphenyl)pyrene (TBAP-α). The self-association of this chemical–biological hybrid system is facilitated by hydrogen bonds, as revealed by molecular dynamics simulations. Within this hybrid photobiocatalyst, TBAP-α functions as an antenna for visible-light absorption and exciton generation, supplying electrons for sacrificial hydrogen production by H–S in aqueous solutions. This coordination allows the hybrid nanoreactor, H–S|TBAP-α, to execute hydrogen evolution exclusively driven by light irradiation with a rate comparable to that of photocatalyst-loaded precious cocatalyst. The established approach to constructing new light-driven biocatalysts combines the synergistic power of biological nanotechnology with the multilength-scale structure and functional control offered by supramolecular organic semiconductors. It opens up innovative opportunities for the fabrication of biomimetic nanoreactors for sustainable fuel production and enzymatic reactions.

## Introduction

Solar energy harvesting and conversion have the potential to contribute to a sustainable, carbon-neutral environment.^1−3^ In nature, multicomponent photosynthetic systems capture solar energy and use enzymatic cascades to orchestrate charge separation and catalytic proton-coupled electron transfer reactions to produce diverse natural products, albeit with certain intrinsic limitations, such as a relatively narrow light-harvesting window and susceptibility to chemical and structural damage by higher energy photons.^4−7^ Artificial photocatalysts employ synthetic materials, often inorganic semiconductors, to mimic natural photosynthetic systems, typically by combining various components to achieve the essential functions.^8^ For example, semiconductor particles have the capability of absorbing light, and when platinum nanoparticles are placed on the surface of the semiconductor, they could facilitate the reduction of protons to generate hydrogen gas.^9−11^ While these synthetic hybrids can achieve high solar-to-hydrogen efficiencies, particularly in the ultraviolet range of the solar spectrum,^12^ they lack the complex, hierarchically structured functionality and selectivity that are present in natural photosynthetic systems. Moreover, it is still challenging to develop artificial systems that can use the whole solar spectrum, and the most effective direct photocatalysts for overall water splitting operate in the ultraviolet spectral range, which constitutes only about 7% of total solar energy.^13^

One promising strategy to leverage the advantages of both biological and abiotic systems is to synergistically integrate natural biological machinery (such as enzymes, organelles, or whole cells) with artificial materials to construct new chemical–biological hybrid systems.^14−17^ Such hybrid systems can, in principle, combine broadband light absorption and high exciton generation efficiency from synthetic semiconductors and the selectivity and catalytic power of living biocatalysts. Chemical–biological hybrids can be generated through surface engineering (e.g., surface ionization^18,19^), redox mediator addition (e.g., methyl viologen^20^), and enzyme encapsulation in porous materials.^21^ However, establishing an efficient physical-chemical connection between biological and nonliving materials, which is crucial for facilitating electron cascades and energy transfer to catalytic centers and exceeding the catalytic potential of each component, poses a significant challenge.^22^ A major obstacle lies in the incompatibility between biocatalysts and synthetic materials in their working environment. Thus, there is a pressing need for more designable and effective approaches to constructing chemical–biological hybrid nanoreactors, allowing for precise structural control across various length scales.

Carboxysomes are anabolic bacterial microcompartments (BMCs) responsible for biological carbon fixation in cyanobacteria and some chemoautotrophic bacteria.^23−25^ The carboxysome sequesters the central CO_2_-fixing enzyme, Ribulose-1,5-bisphosphate carboxylase/oxygenase (RuBisco), and carbonic anhydrase (CA) within a self-assembling proteinaceous shell. The carboxysome shell is composed of numerous protein paralogs that are in the form of hexamers and pentamers and allows the entry of HCO_3_^–^ to create a CO_2_-rich microenvironment for facilitating carboxylation.^26−28^ These natural features of carboxysomes have inspired the design and reprogramming of carboxysome structures by using synthetic biology for various biotechnological applications. In addition, hydrogenases are native catalysts for H_2_ production in bacteria, archaea, and some eukaryotes with exceptional catalytic activity and efficiency and act as valid candidates for biological H_2_ production.^29,30^ In particular, [FeFe]-hydrogenases are highly active for H_2_ generation but are limited by their high oxygen sensitivity and irreversible inactivation by oxygen.^31^ Therefore, the carboxysome shell has demonstrated great potential to serve as novel nanoreactors to boost H_2_ evolution.^32,33^ However, the reported carboxysome-based H_2_-evolving nanoreactors require a constant supply of electrons from methyl viologen, which is not sustainable.

Here, we report a bottom-up approach to constructing a visible-light-driven chemical–biological hybrid nanoreactor by interfacing a porous crystalline organic semiconductor, microporous α-polymorph of 1,3,6,8-tetra(4′-carboxyphenyl)pyrene (TBAP-α), with recombinant α-carboxysome shells (with an average diameter of 90 nm) that encase [FeFe]-hydrogenases (H–S).^33^ The resulting hybrid photobiocatalyst was characterized by electron microscopy, confocal microscopy, isothermal titration calorimetry, photoluminescence and photoelectrochemical assays, and computational simulations. Our results demonstrate that the intimate bio- and abiotic interface, established through extensive hydrogen bonds, mediates charge transport between the organic semiconductor and the biocatalyst. This allows the light-driven hybrid nanoreactor (H–S|TBAP-α) to exhibit a high sacrificial H_2_-evolution efficiency compared to the photocatalyst alone, and the efficiency of this photobiocatalyst is comparable with analogous synthetic photocatalysts that use expensive precious metals, such as platinum.

## Results and Discussion

### Preparation of Porous Crystalline Photocatalyst TBAP-α

Light-driven chemical–biological hybrid nanoreactors should comprise light-harvesting antenna with strong visible-light absorption, high exciton production efficiency in moderate aqueous solution, and favorable hydrolytic stability to integrate with biological machinery with robust stability and high catalytic conversion. Hydrogen-bonding organic framework (HOF) photocatalysts that contain discrete hydrogen-bonding molecular building blocks, in principle, possess great potential for this application due to their inherent structure-tunability, functionality, and solution processability.^34^

**Figure 1:**
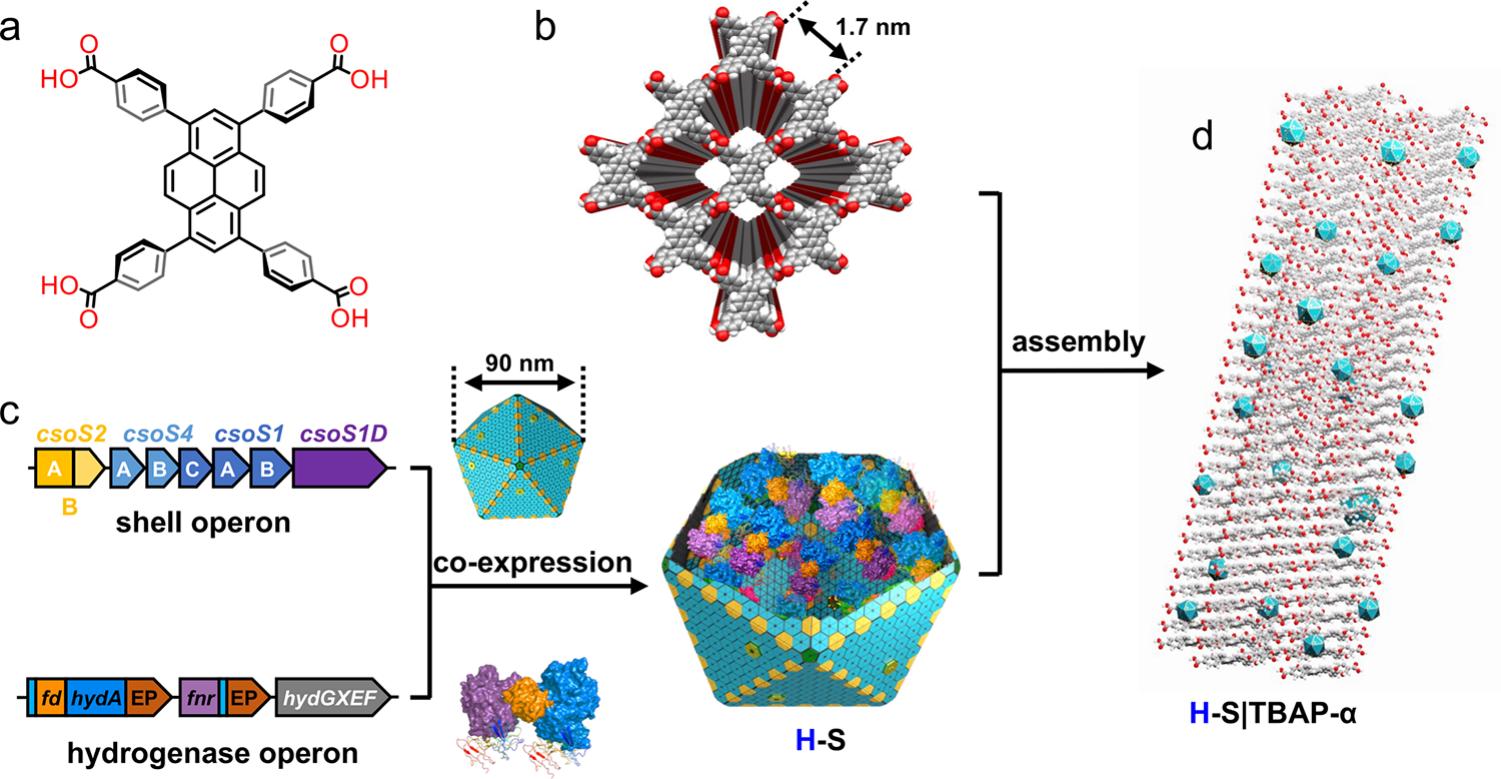
Generation of the organic semiconductor, the hydrogenase-containing carboxysome shell, and the chemical–biological hybrid nanoreactor, H–S|TBAP-α. (a) Chemical structure of 1,3,6,8-tetra(4′-carboxyphenyl)pyrene (TBAP). (b) Crystal structure of α-polymorph of 1,3,6,8-tetra(4′-carboxyphenyl)pyrene (TBAP-α). (c) Genetic organization of the α-carboxysome shell operon and hydrogenase-expressing operon for the production of recombinant shells and active [FeFe]-hydrogenases in *E. coli*, respectively, and a schematic model of the hydrogenase-shell nanoreactor (H–S). EP: CsoS2 C-terminus as the encapsulation peptide. The *hydGXEF* genes encode the crucial maturase enzymes for hydrogenase formation and activation, including HydE, HydF, and HydG. PDB ID: Ferredoxin (Fd)-[FeFe]-hydrogenase, 2N0S; ferredoxin-NADP reductase (FNR), 2XNJ. (d) 3D model representation of the H–S|TBAP-α hybrid system, in which H–S adheres to the hydrogen-bonding groups on the TBAP-α crystal surface.

1,3,6,8-tetra(4′-carboxyphenyl)pyrene molecule (TBAP) has been investigated as a building block in HOFs,^35^ metal–organic frameworks (MOFs),^36^ and protein-HOFs assemblies.^37^ TBAP has a pyrene core that can π–π stack and carboxylic acid groups that can hydrogen-bond (Figures S1–S3), making it an ideal candidate for assembling HOFs. The α-polymorph of TBAP, referred to hereafter as TBAP-α, was successfully crystallized by slow diffusion of chloroform (CHCl_3_) into a solution of TBAP dissolved in dimethylformamide (DMF).^35^ In the TBAP-α structure, 2-D layers of hydrogen-bonded TBAP molecules are closely packed and interact through π–π stacking of the pyrene units, which are separated by 0.38 nm. This generates a columnar microporous structure with 1.7 nm 1-D pores and unsaturated hydrogen-bonding layers on the surfaces of the needle-shaped crystals (Figures 1b and S4). Here, we characterized TBAP-α using single-crystal X-ray diffraction (sc-XRD) (Table S1), powder X-ray diffraction (PXRD), and gas absorption analysis of the micrometer-sized needle-shaped crystals (Figure S5).

TBAP-α exhibited remarkable visible-light absorption (Figure S6) and semiconducting properties with an optical bandgap of 2.30 eV and a conduction band (C.B.) of −1.15 V (Figure S7), determined by using the Mott–Schottky technique, performing a considerable thermodynamic driving force for H_2_ production from water. In addition, TBAP-α favors neutralized ascorbic acid (AA) aqueous solution as the sacrificial agent,^35,38^ a commonly used sacrificial agent in artificial photosynthesis due to its solubility in water and the compatibility to work in a neutral medium.^39^

### Self-Association of α-Carboxysome Shells and TBAP-α

**Figure 2:**
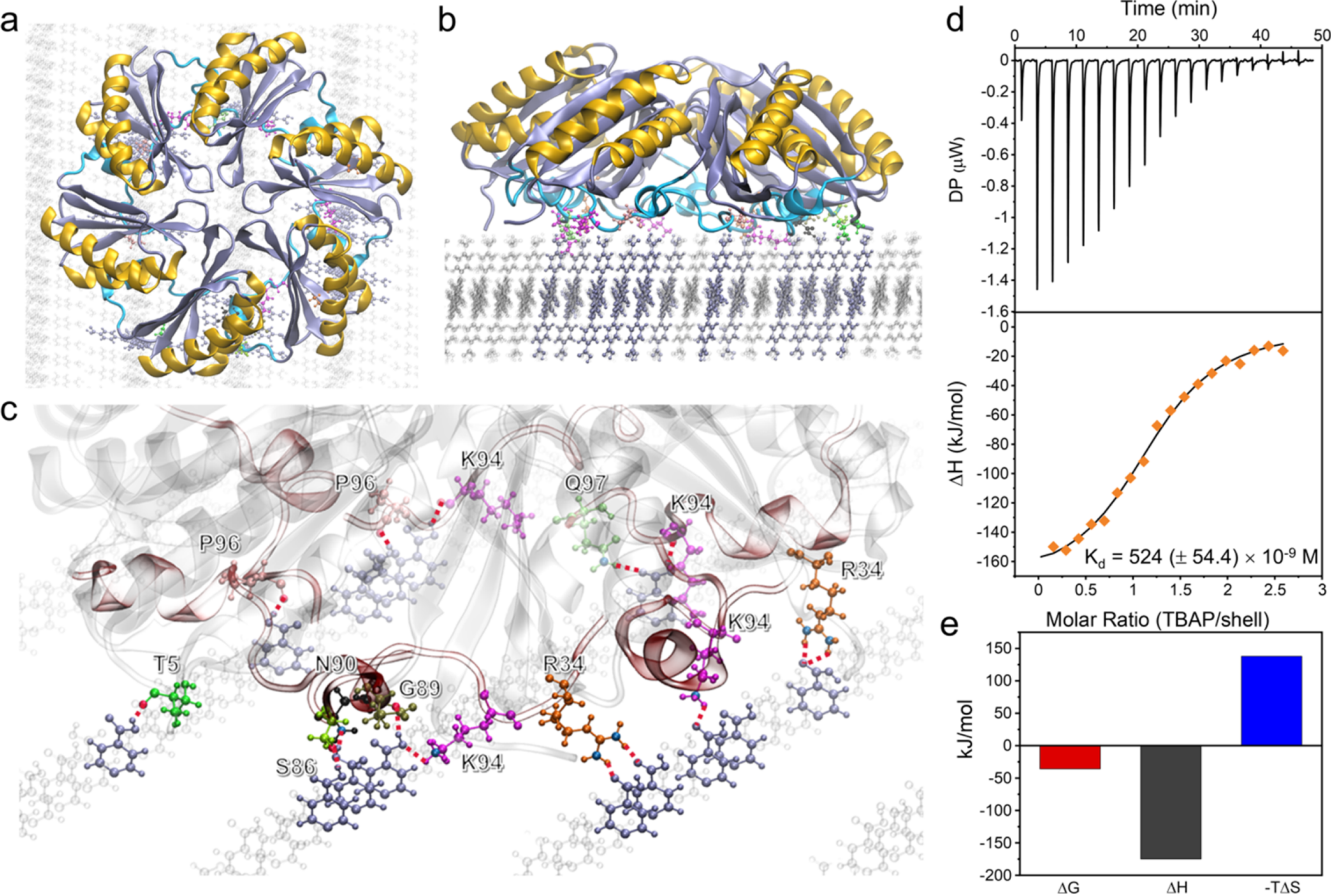
Binding between the α-carboxysome shell and TBAP-α. Results of molecular dynamics simulations of CsoS1A (PDB ID: 2EWH) and TBAP-α binding equilibrium states are shown in panels (a–c). (a) Front view. (b) Side view, showing the simulated binding interface between TBAP-α and the CsoS1A hexamer. (c) Zoom-in view of the noncovalent interactions (red dotted lines) formed between amino acid residues of CsoS1A (Pro96, Thr5, Ser86, Asp90, Gly89, Lys94, Arg34, Glut97) and TBAP-α (blue = N atoms, red = O atoms). (d) ITC thermogram resulting from the titration of TBAP (50 μM) into the α-carboxysome shell (3.75 μM) in TN buffer (20 mM Tris-HCl (pH = 8.0), 150 mM NaCl). (e) Thermodynamic profile of TBAP binding to α-carboxysome shell in TN buffer. Δ*G*, change in Gibbs Free Energy; Δ*H*, change in enthalpy; *T*, temperature in Kelvin; Δ*S*, change in entropy.

The recombinant α-carboxysome shell is composed of CsoS1 and CsoS4 paralogs, as well as the linker protein CsoS2 that is attached to the inner surface of the shell.^32,33^ CsoS2 exists as two distinct isoforms in *Halothiobacillus neapolitanus*: the longer form CsoS2A (∼130 kDa) and the short form CsoS2B (∼85 kDa).^40^ The two hexameric shell proteins, CsoS1A and CsoS1C, which show 98% similarity in amino acid sequence, represent the most abundant components on the α-carboxysome shell^23^ (Table S2). To verify the physical association between α-carboxysome shells and TBAP-α, we performed molecular dynamics (MD) simulations to study the binding between CsoS1A and TBAP-α, using the crystal structure of CsoS1A (PDB: 2EWH) and TBAP-α structure determined by sc-XRD. The calculated binding free energy is −22.10 kcal mol^–1^ by the MM/GBSA method in AmberTool22^41^ using the last 30 ns MD trajectory (150 frames) of the TBAP-α and CsoS1A protein binding simulation. MD simulations suggest that the residues mainly in the loop regions of CsoS1A, including Thr5, Arg34, Ser86, Gly89, Asp90, Lys94, Pro96, and Glu97, form strong interactions with the net-carboxylate on the surface of TBAP-α through hydrogen-bonding and salt bridges (Figure 2a–c and Tables S3 and S4). These amino acid residues are conserved among the main hexamer shell proteins, as indicated by multiple sequence alignment (Figure S8) and structural analysis (Figure S9), suggesting that the TBAP-α crystal surface interacts with α-carboxysome shells through cooperative hydrogen-bonding interactions with the shell proteins.

Next, the binding potential of the whole recombinant α-carboxysome shells and molecular TBAP-α was detected by using isothermal titration calorimetry (ITC) (Figure 2d). A low dissociation constant (*K*_d_) was measured as 524 (±54.4) × 10^–9^ M, indicating strong affinity between α-carboxysome shells and TBAP-α. In addition, the negative Δ*G* value indicates that the association between α-carboxysome shells and TBAP-α is energetically favorable, while the Δ*H* value reveals that this process is enthalpy-driven, suggesting that hydrogen bonds and van der Waals interactions may be the main driving forces in the association process (Figure 2e). The ITC results were consistent with our computational findings.

**Figure 3:**
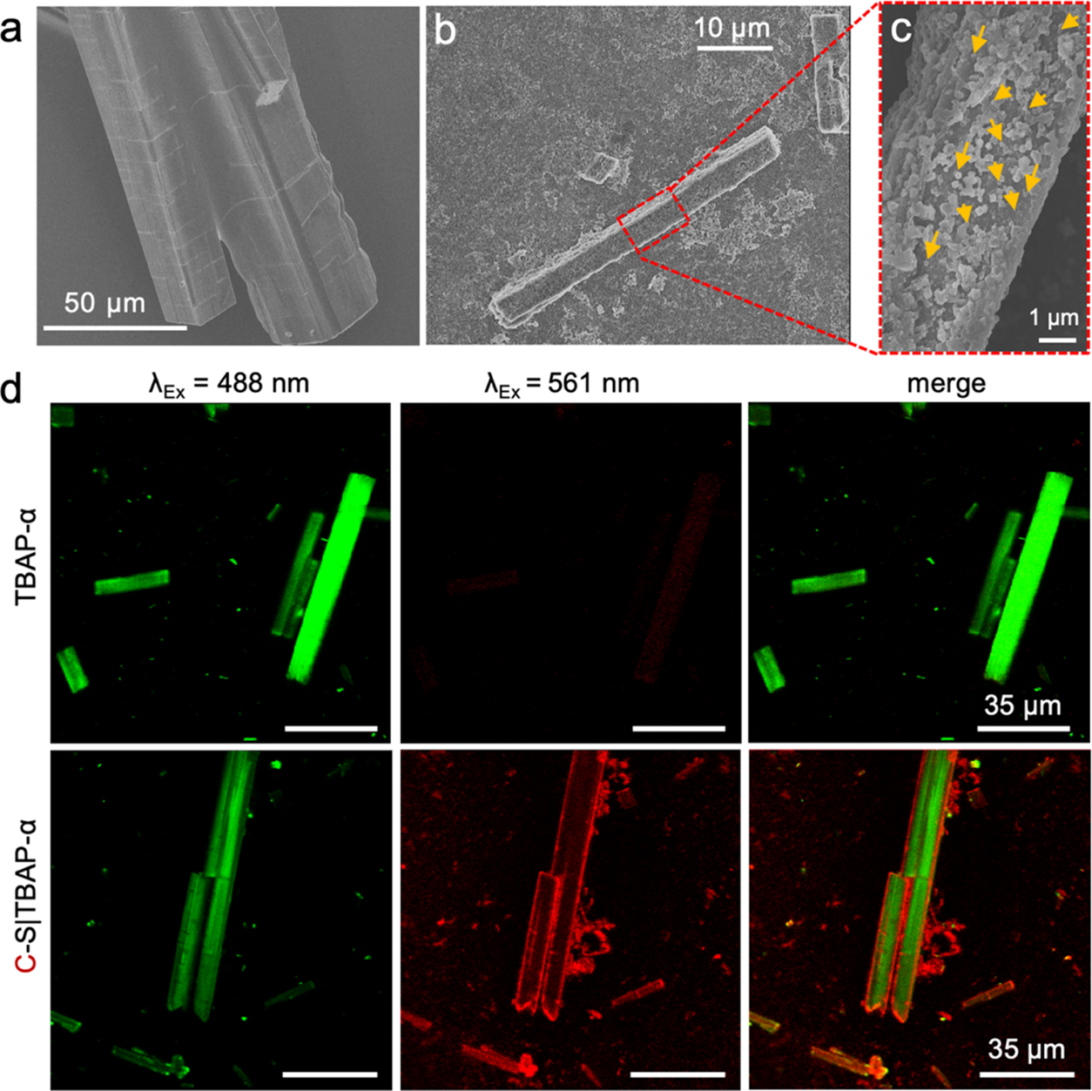
Attachment of mCherry-encapsulated C–S shells on the surface of TBAP-α crystals. SEM images of (a) uncoated TBAP-α, (b) C–S|TBAP-α (40 × 3.6 μm^2^ size), and (c) zoom-in view of C–S|TBAP-α. Orange arrows show the C–S particles that were attached to the TBAP-α crystal surface. (d) Fluorescence images of TBAP-α only (top row) and the C–S|TBAP-α hybrid (bottom row). Left, excitation at 488 nm; middle, excitation at 561 nm; right, the merged channel. All fluorescence images were adjusted to have the same brightness and contrast settings.

To directly visualize the association between α-carboxysome shells and TBAP-α crystals, we generated recombinant α-carboxysome shells that encapsulate mCherry, by fusing mCherry with the encapsulation peptide CsoS2 C-terminus (mCherry-EP) and coexpressing mCherry-EP with shell proteins. This approach led to the generation of mCherry-EP-Shell (C–S) (Figures S10 and S11). The purified C–S and TBAP-α were then incubated in solution for 30 min to enable self-association. Scanning electron microscopy (SEM) confirmed the coating of C–S particles on the surfaces of needle-shaped TBAP-α crystals (Figure 3a–c). Moreover, mCherry exhibits a distinct absorption maximum (λ_Ex_ = 587 nm) from that of TBAP-α (λ_Ex_ = 390 nm). Confocal laser scanning microscopy (CLSM) showed that the needle-shaped TBAP-α crystals exhibit bright fluorescence when excited at 488 nm and very weak red fluorescence when excited at 561 nm (Figure 3d, top row). In contrast, strong red fluorescence was recorded along the surface of TBAP-α crystals incubated with C–S when excited at 561 nm, indicating the association of C–S with the TBAP-α crystal surface (Figure 3d, bottom row). Both SEM and CLSM results demonstrate that the surfaces of TBAP-α crystals were densely coated with C–S particles in an aqueous suspension. In comparison, a bulk sample of unfunctionalized pyrene crystals^42^ was incubated with C–S using the same procedure to explore the contribution of the organized layer of unsaturated carboxylic acid groups on the TBAP-α surface. The SEM images showed no visible C–S particles on these pyrene crystals (Figure S12), indicating that unsaturated carboxylic acid groups serve as the main binding sites between TBAP-α and α-carboxysome shells.

### Construction of a Light-Driven Biomimetic Hybrid Nanoreactor for Hydrogen Production

Given the unique properties of TBAP-α for light absorption and electron–proton transfer^35^ as well as the strong binding between TBAP-α crystals and α-carboxysome shells, we hypothesized that the C–S|TBAP-α hybrid may serve as a novel biomimetic photocatalyst enabling electron transfer cascade and enhanced enzymatic performance in biotechnological applications. Intriguingly, sulfate ions can be soaked into the major shell hexamers of both α- and β-carboxysomes,^43,44^ indicating that the pores may act as the conduits for negatively charged metabolites. Furthermore, iron–sulfur (Fe–S) clusters have been found in catabolic BMCs,^45−48^ which could potentially facilitate the transfer of electrons and protons from external TBAP-α crystals across the shell to retain the internal redox environment.^49,50^

**Figure 4:**
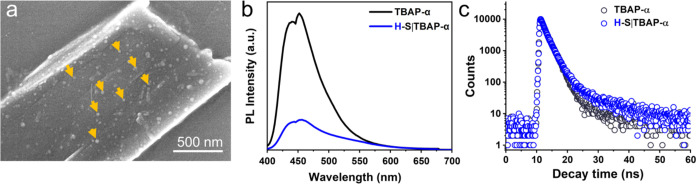
Characterization of the light-driven H–S|TBAP-α hybrid nanoreactor. (a) SEM image of H–S|TBAP-α. Orange arrows show the H–S particles that were attached to the TBAP-α crystal surface. (b) Photoluminescence (PL) spectrum and (c) Excited-state lifetimes of H–S|TBAP-α and TBAP-α alone in 0.1 M neutralized AA aqueous solution when excited at 390 nm.

To test the hypothesis, we chose the α-carboxysome shells that encase the oxygen-sensitive redox enzymes, hydrogenases, that bind to the TBAP-α crystals. The hydrogenase-containing shells (H–S) were generated by encapsulating [FeFe]-hydrogenase fused with the CsoS2 C-terminus (HydA-EP), ferredoxin (Fd), and ferredoxin: NADP^+^-oxidoreductase (FNR) into the α-carboxysome shells, along with coexpression of the crucial maturase enzymes HydE, HydF, and HydG^33^ (Figure 1c). H–S has been reported to exhibit enhanced H_2_ evolution and O_2_ tolerance, taking advantage of the O_2_-limiting microenvironment created within the α-carboxysome shell.^32,33^ The comparable ζ-potentials of empty α-carboxysome shell (S), mCherry-encapsulated α-carboxysome shell (C–S), and [FeFe]-hydrogenase-encapsulated α-carboxysome shell (H–S), as well as the more negative ζ-potentials of S, C–S, and H–S after binding with TBAP-α individually, indicated that binding of the α-carboxysome shells to the TBAP-α surface was not remarkably affected by cargo encapsulation (Figure S13). Electron microscopy further confirmed that the TBAP-α crystals were coated with H–S particles (Figures 4a and S14), consistent with the observations of C–S shells and TBAP-α crystals (Figure 3).

**Figure 5:**
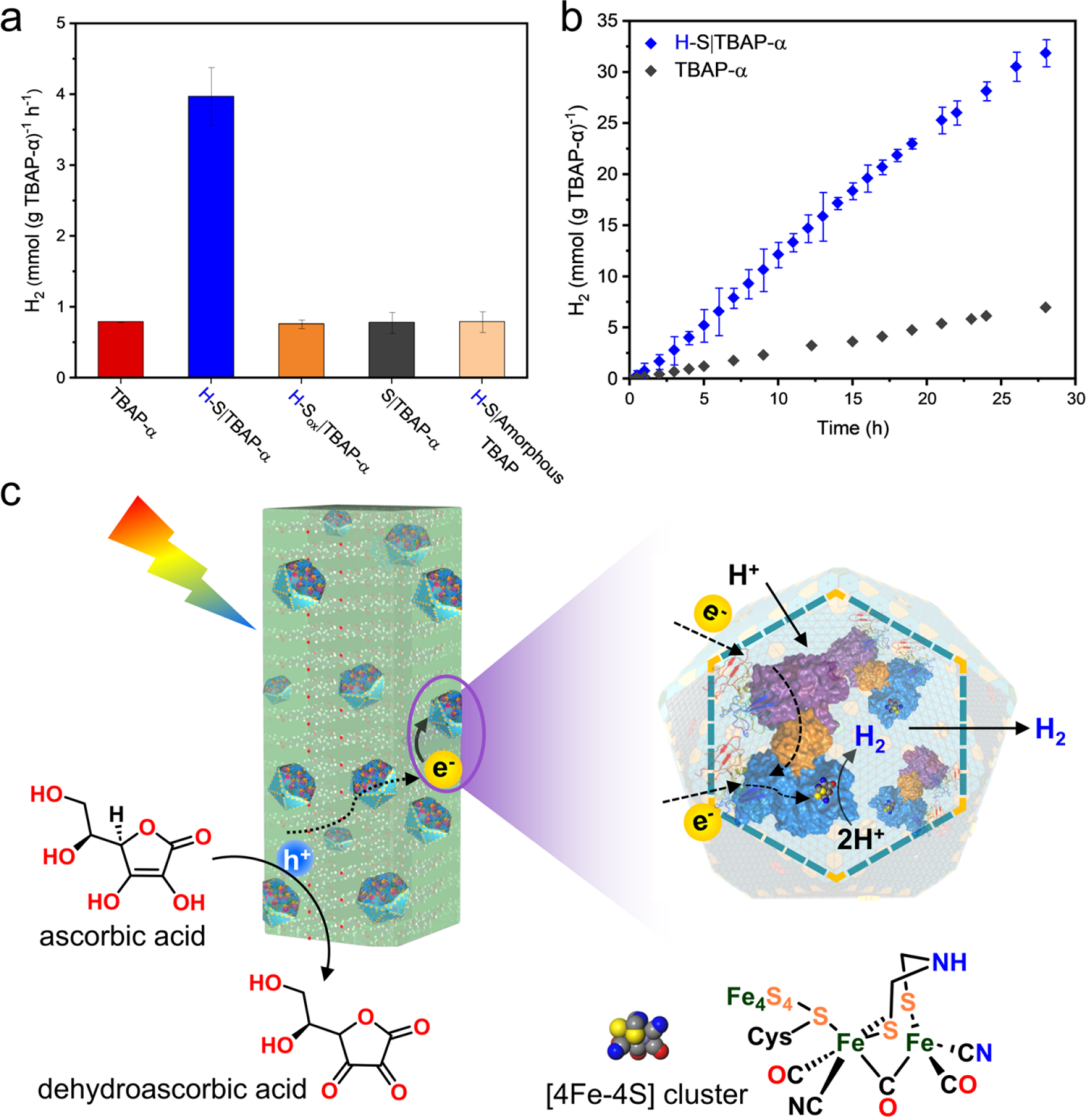
Visible-light-driven hydrogen production of H–S|TBAP-α. (a) H_2_-evolution rates of TBAP-α, H–S|TBAP-α, H–S_ox_|TBAP-α, S|TBAP-α, and H–S|Amorphous TBAP irradiated by a solar simulator for 2 h. (b) H_2_ evolution of TBAP-α (black dots) and H–S|TBAP-α (blue dots) as a function of time (λ > 420 nm). (c) Schematic diagram of proposed electron transfer pathway in H–S|TBAP-α for hydrogen production. Electrons generated from TBAP-α irradiation are transferred through holes on the α-carboxysome shell to the 4Fe–4S cluster via FNR or directly for proton reduction. Ascorbic acid acts as a sacrificial agent to prevent the photogenerated hole–electron recombination. Error bars represent the SD of the mean of three independent experiments.

The H–S|TBAP-α hybrid biocatalyst (Figure 1d) was generated by dispersing freshly purified H–S and TBAP-α in nitrogen-degassed neutralized AA solution (Figures S15 and S16), followed by incubation for 30 min at room temperature. UV–vis spectroscopy was used to examine the colloidal stability of H–S|TBAP-α. No significant changes were detected, even after 19 h incubation (Figure S17). We also employed photoluminescence (PL) spectroscopy to analyze the electron–hole pair recombination performance of H–S|TBAP-α and TBAP-α. The PL emission intensity of H–S|TBAP-α was quenched greatly compared to TBAP-α alone when excited at 390 nm (Figure 4b), indicating that the recombination rate for the photogenerated electron and hole was drastically reduced. This is beneficial for the transfer of electrons to H–S for the H_2_ production reaction. In addition, time-correlated single-photon counting (TCSPC) showed that the average weighted lifetime of H–S|TBAP-α (τ_avg_ = 4.11 ns) was greater than that of TBAP-α (τ_avg_ = 3.51 ns) in an aqueous suspension (Table S4), suggesting that more electrons were transferred to H–S rather than recombining with holes. Moreover, TBAP-α in aqueous suspension exhibited a shorter lifetime (τ_avg_ = 2.00 ns),^35^ which aligns with its remarkably lower H_2_ production performance, even when hybridized with H–S (0.79 mmol (g TBAP)^−1^ h^–1^, Figure 5a). Therefore, the PL quench and the extended lifetime of H–S|TBAP-α indicate that H–S facilitated the separation of electron–hole pairs.

The electron transfer performance of TBAP-α and H–S|TBAP-α was characterized by transient photocurrent (TPC) and electrochemical impedance spectroscopy (EIS). The transient photocurrent intensity of H–S|TBAP-α reached a value that was 30% higher than that of TBAP-α alone (Figure S18a), indicating that photogenerated electrons and holes could be separated and transferred effectively to H–S in H–S|TBAP-α. The arc radius of EIS plots varied in the order H–S|TBAP-α < TBAP-α (Figure S18b), suggesting that H–S|TBAP-α showed lower electrical resistance than the TBAP-α crystalline, likely through enhanced charge transport. These assays confirmed the effective construction of the biomimetic nanoreactor with accelerated electron transfer and promising photocatalytic potential.

High-throughput sacrificial H_2_ evolution measurements revealed that the mass-normalized H_2_ evolution rate of H–S|TBAP-α (approximately 25 wt % of H–S) was 3.97 ± 0.41 mmol (g TBAP-α)^−1^ h^–1^ during 2 h irradiation in nitrogen-degassed neutralized AA aqueous solution (0.1 M) using a solar simulator (AM1.5G, 1440 W xenon, see Methods for details). The rate is almost 5 times higher than that obtained from TBAP-α alone under the same conditions (0.79 ± 0.01 mmol (g TBAP-α)^−1^ h^–1^; Figure 5a). The H_2_-evolution activity of TBAP-α alone is associated with the residual palladium present in the sample, which originates from the synthesis of TBAP and acts as a cocatalyst.^35^ When empty shell (S) and deactivated H–S with oxygen bubbling (H–S_ox_) (the deactivation of H–S_ox_ was confirmed by sodium dithionite-methyl viologen assays shown in Figure S19a) were mixed with TBAP-α respectively, the H_2_-evolution rates of both the resulting S|TBAP-α and H–S_ox_|TBAP-α declined significantly (0.78 ± 0.15 mmol (g TBAP-α)^−1^ h^–1^ for S|TBAP-α, 0.76 ± 0.06 mmol (g TBAP-α)^−1^ h^–1^ for H–S_ox_|TBAP-α), which were comparable to the amount of H_2_ produced by TBAP-α alone (Figure 5a). These results indicated that H–S in the H–S|TBAP-α hybrid nanoreactor was enzymatically active. Furthermore, no detectable amounts of H_2_ were produced from H–S|TBAP-α in a dark suspension, an irradiated suspension of H–S in a neutralized AA aqueous solution (0.1 M), or an irradiated suspension of H–S|TBAP-α in pure water over 2 h. Together, these control experiments indicate that TBAP-α donated photogenerated electrons for proton reduction and AA acted effectively as the hole scavenger (Figure S19 and Table S6). When TBAP-α was associated with the α-carboxysome shells that encapsulate only hydrogenases without FNR and Fd (HydA-S), there was a 33% decrease in the H_2_-evolution rate compared to H–S|TBAP-α (2.68 ± 0.45 mmol (g TBAP-α)^−1^ h^–1^ for HydA-S|TBAP-α, 3.97 ± 0.41 mmol (g TBAP-α)^−1^ h^–1^ for H–S|TBAP-α) (Figure S19d). These results suggest that FNR and Fd indeed contributed to the transport of photogenerated electrons to the active sites of hydrogenases.

One advantage of crystalline photocatalysts is their ability to accelerate electron transfer through a highly ordered packing pattern. This can be verified by a larger photocurrent density and smaller EIS radius from TBAP-α compared to amorphous TBAP (Figure S20). This observation coincides with the substantially higher H_2_ produced when H–S was hybridized with TBAP-α compared with an amorphous powdered TBAP sample. Specifically, the crystalline TBAP-α produced 3.97 ± 0.41 mmol^–1^ (g TBAP-α)^−1^ h^–1^, while the amorphous TBAP sample yielded only 0.79 ± 0.15 mmol^–1^ (g TBAP)^−1^ h^–1^ under the same conditions (Figure 5a). Hence, the synergy between H–S and TBAP-α in the hierarchical H–S|TBAP-α assemblies appears to be critical for the attached H–S structures, ensuring electron transfer between the two components and enhanced hydrogen evolution performance. The apparent quantum efficiency (AQY) was measured at different wavelengths to evaluate the photocatalytic H_2_ production performance of H–S|TBAP-α. The AQY measured at 420 nm was 2.8% over a 1.5 h experiment (Figure S21).

To examine the H_2_-evolution performance of H–S|TBAP-α in long-term irradiation, the H–S|TBAP-α hybrid nanoreactors were exposed to visible light (300 W Xe light, λ > 420 nm). As depicted in Figure 5b, H–S|TBAP-α exhibited a constant increase in H_2_ production as a function of time. Under the same conditions, H–S|TBAP-α produced an H_2_ efficiency that was roughly comparable to the noble metal-loaded system as reference, namely, 1 wt % platinum metal cocatalyst deposited on TBAP-α (Pt|TBAP-α), after 14 h of irradiation (144.3 μmol for H–S|TBAP-α, 170.2 μmol for Pt|TBAP-α) (Figure S22). In contrast, TBAP-α, without the addition of platinum cocatalyst or H–S, produced only 31.9 μmol of H_2_ after 14 h irradiation (Figure S22). The H_2_-production ability of H–S|TBAP-α ahowed a relatively steady increase as a function of time for 30 h irradiation, with an H_2_-evolution rate of 1,137.8 ± 47.2 μmol (g TBAP-α)^−1^ h^–1^. Intriguingly, H–S|TBAP-α exhibited remarkable stability during 5 consecutive runs over a total reaction time of 25 h, with no notable decline in the H_2_-production rate observed after the 5 cycles (Figure S23). From the listed state-of-the-art photocatalyst-hydrogenase hybrids (Table S7), H–S|TBAP-α has the unique advantage of considerable stability with a promising H_2_ production efficiency. In the long-term H_2_ evolution experiments, the HydA content was measured using purified HydA as a reference (Figure S24), and the total turnover number (TON) of H–S|TBAP-α was measured as 5,411,174 mol H_2_ (mol H_2_ase)^−1^ on average after 24-h irradiation (Xe lamp, λ > 420 nm), which is comparable to the reported organic materials-hydrogenase hybrids (Table S7). SEM was performed to examine the morphology changes of H–S|TBAP-α after 30 h of continuous irradiation. The attachment of H–S nanoparticles on the TBAP-α surface was not as obvious as before the illumination (Figure S25). Meanwhile, cracks appeared on the crystal surface, which is consistent with the crystallinity decrease found in the PXRD pattern (Figure S26).

Overall, our findings demonstrate the catalytic efficiency and robustness of H–S|TBAP-α under irradiation and the fact that the hybrid H–S|TBAP-α system has an enhanced photocatalytic H_2_-evolution capacity relative to the organic semiconductor TBAP-α crystal or H–S alone. We speculate that the enhanced photocatalytic H_2_-evolution performance of H–S|TBAP-α is partially due to accelerated electron flux from TBAP-α, through the ion-containing pores of shell protein complexes at the biotic–abiotic interface, to the catalytically active centers ([4Fe–4S] cluster) of encapsulated [FeFe]-hydrogenases, via FNR/Fd proteins or directly (Figure 5c).

## Conclusions

We developed a bottom-up strategy for biomimetic nanoreactor construction based on the inherent structural properties of biological machinery and synthetic organic materials. A crystalline HOF, TBAP-α, acts as a visible-light-capture organic antenna and can funnel energy into a complex, multicomponent biocatalytic nanoreactor, H–S, which mimics the natural photosynthesis process. This hybrid nanoreactor performs as a light-driven photocatalyst for sacrificial H_2_ evolution in water, and it exhibits improved activity than H–S or TBAP-α alone and comparable activity to the TBAP-α material deposited with 1 wt % of the precious metal cocatalyst, platinum. Our study suggests that H–S|TBAP-α is attributed to the separation of the photogenerated electron and hole pairs at the TBAP-α surface and the transport of electrons into the nanoreactor interior, which enhances the catalytic performance of encapsulated hydrogenases for the production of molecular hydrogen. The construction of H–S|TBAP-α with considerable hydrogen production activity acts as a promising model system to explore the potential of combining catalytic advantages from both artificial materials and natural machinery for solar energy conversion.

The successful assembly of complex, hierarchical structured nanoreactors using multiple noncovalent hydrogen-bonding interactions on organic semiconductor surfaces opens up avenues for designing new families of chemical-biologically active hybrid systems for reactions other than H_2_ production. Here, we demonstrated the generality and versatility of our approach, which utilizes noncovalent hydrogen-bonding interactions between multiple proteins and the organic semiconductor surface. We coated the TBAP-α crystal surface with three different proteins, C–S, H–S, and H–S_ox_, each with distinct catalytic properties. We postulate that this approach could be adapted to create biocatalytic nanoreactors for more complex chemical transformations by altering the cargo enzymes within the α-carboxysome shell.

## Methods

### Generation of Constructs

All assemblies between genes and linearized vectors were achieved by Gibson assembly (Gibson assembly kit, New England BioLabs, U.K.). The *mCherry* gene was cloned to pCDFDueT-1 linearized by *Eco*RI and AscI to generate the pCDFDueT-mCherry vector. The *mCherry* gene with the nucleotide sequence encoding EP fused at the C-terminus was cloned into pCDFDueT-1 and in the frame to create pCDFDueT-mCherry-EP, using primer pairs M-F/M-C-R and M-C-F/C-R (Table S8). The *mCherry* gene fused to the N-termini of these truncated CsoS2 C-terminal regions was inserted into pCDFDueT linearized by *Bam*HI and AscI. These constructs were verified by PCR and DNA sequencing and transformed into *Escherichia coli* BL21(DE3) cells. Plasmid maps (Figure S27) and sequences (Table S9) are available in the Supporting Information.

### Coexpression and Generation of α-Carboxysome Encapsulated with mCherry (C–S)

*E. coli* strains containing the *pCDTDuet-mCherry-CS2* plasmid and *pBAD-cso-2* plasmid were cultivated in LB medium containing 50 μg mL^–1^ of spectinomycin and 100 μg mL^–1^ of ampicillin. The mCherry expression was induced by adding 0.5 mM isopropyl β-d-1-thiogalactopyranoside (IPTG) at OD_600_ = 0.6–0.8. After 4 h induction of the mCherry-EP expression, the shell expression was induced by 1 mM l-Arabinose, and cells were then grown at 25 °C for 16 h with constant shaking. The mCherry induction was performed before the expression of shells to allow mCherry protein expression before shell encapsulation. The cells were then harvested by centrifugation at 5000*g* for 10 min. The cell pellets were washed with TN buffer (20 mM Tris-HCl pH = 8.0, 150 mM NaCl) and resuspended in TN buffer supplemented with 10% (v/v) CelLytic B cell Lysis reagent (Sigma-Aldrich) and 1% Protein Inhibitor Cocktail (100×) (Sigma-Aldrich). The cell suspensions were lysed by French Press (Stansted Fluid Power, U.K.). Cell debris was removed by centrifugation at 10,000*g* at 4 °C for 10 min, followed by centrifugation at 50,000*g* to enrich C–S at 4 °C. The pellets were resuspended in TN buffer and then loaded onto sucrose gradients (10–30% or 10–50%, w/v), followed by ultracentrifugation (Beckman, XL100K Ultracentrifuge) at 105,000*g* for 30 min. Each sucrose fraction was collected and stored at 4 °C. Electron microscopy, confocal microscopy, SDS-PAGE, and immunoblot analysis were performed, as described in the Supporting Information.

### Expression of Mature [FeFe]-Hydrogenase and Generation of α-Carboxysome Shells with Encapsulated Hydrogenases (H–S)

[FeFe]-hydrogenase from the green alga *Chlamydomonas reinhardtii* was expressed and encapsulated into α-carboxysome shells (H–S) in *E. coli*, as reported before.^24^ In summary, *E. coli* strains containing the *pCDFDuet-hydA-CS2* (GenBank accession code AAL23572.1) plasmid and the *pBAD-cso-2* plasmid were cultivated in LB medium containing 0.2 mM ferric ammonium citrate, 50 μg mL^–1^ spectinomycin, and 100 μg mL^–1^ ampicillin and degassed with N_2_ for 30 min at OD_600_ = 0.6–0.8. The hydA-EP expression was induced by the addition of 0.5 mM IPTG. After 4 h induction of the hydA-EP expression, the shell expression was induced by 1 mM l-Arabinose, and cells were then grown at 25 °C for 16 h. The purification of H–S was carried out following the method for C–S mentioned above. It should be noted that H–S should be expressed and purified under anaerobic conditions, and all of the solutions used should be degassed in advance. As a control, the plasmid for expressing hydrogenases without FNR and Fd (*pCDFDuet-hydA-hydGxEF-CS2*) was generated by deleting both genes encoding FNR and Fd from the *pCDFDuet-hydA-CS2* plasmid. The α-carboxysome shells that encapsulate hydrogenases only without FNR and Fd (HydA-S) were generated following the same procedure as that for H–S.

### TBAP-α Construction

Synthesis and characterization of 1,3,6,8-tetra(4′-carboxyphenyl)pyrene (TBAP) are described in detail in the Supporting Information. As-synthesized TBAP powder (100 mg) was dissolved in DMF (8 mL). The supernatant solutions were filtered into 25 mL vials using a 0.22 μm PTFE syringe filter to remove any particulates and then placed into larger 100 mL vials with antisolvent CHCl_3_. Vapor diffusion of the antisolvent into the TBAP solution was carried out for several days until the vials were nearly full of solvent. The resulting crystals were activated after the crystallization solvent was exchanged with acetone 10–12 times over a few days. The crystals were then collected by filtration and characterized by PXRD.

### Generation of H–S|TBAP-α Hybrid Nanoreactors

A 2 mg portion of TBAP-α and neutralized AA aqueous solution (0.1 M, 4.5 mL) were added into sample vials and purged with N_2_ to eliminate oxygen in the solution and sample vials. Next, 0.5 mL of H–S solution purified by a sugar gradient strategy (20% sucrose content, thereby diluting the total protein concentration to 1 mg mL^–1^ as measured via the Nanodrop method) was introduced into the sample vials under an N_2_ gas flow. The mixture solution was incubated on a rotator at room temperature for 30 min to facilitate the association of H–S on the TBAP-α surface. The H–S|TBAP-α construction was confirmed by SEM and photocatalytic H_2_ evolution performance.

### High-Throughput Photocatalytic H_2_ Production Experiments

2 mg of TBAP-α or amorphous TBAP powder and neutralized AA aqueous solution (0.1 M, 4.5 mL) were added into separate sample vials (vial size = 12.5 mL) and purged with N_2_ on a Chemspeed Technologies Sweigher platform for 6 h. Next, the purified H–S solution (0.5 mL in 20% sucrose, 1 mg mL^–1^ measured using the Nanodrop method) was added under N_2_ gas flow to make the total volume of 5 mL. For control samples, 0.5 mL of TN buffer was added to the vials to prepare solutions with a total volume of 5 mL.

All sample vials were irradiated using an Oriel Solar Simulator with an output of 1.0 sun (AM1.5G, Class AAA, IEC/JIS/ASTM, 1440 W xenon, 12 × 12 in., MODEL: 94123A) equipped with a rocker/roller device. The vial size was 23 × 46 mm^2^, and the irradiation area on the samples was 16.61 cm^2^. Gaseous products were analyzed on a Shimadzu GC-2010 equipped with a Shimadzu HS-20, injecting a sample from the headspace sampler via a transfer line (temperature 150 °C) onto an Rt-Msieve 5 Å column with He as the carrier gas at a flow rate of 30 mL min^–1^. H_2_ was detected with a barrier discharge ionization detector and referenced against a standard that contained a known concentration of H_2_. Data are represented as the mean ± standard deviation (SD) from at least three repeated measurements.

### Time-Course H_2_ Evolution Assays

Time-course H_2_ evolution measurements were conducted in a 67 mL quartz flask. 10 mg of TBAP-α and 22.5 mL of neutralized AA aqueous solution (0.1 M) were added to the quartz flask and purged with N_2_ for 30 min, followed by the addition of purified H–S solution (2.5 mL in 20% sucrose, 1 mg mL^–1^ measured using the Nanodrop method) under N_2_ gas flow to make the final volume of 25 mL. The reaction mixture was illuminated with a 300 W Newport Xe light source (Model: 6258, Ozone-free) using a λ > 420 nm cutoff filter. The light source was cooled by water circulating through a metal jacket. Gas samples were taken with a gastight syringe and run on a Bruker 450-GC gas chromatograph. H_2_ was detected with a thermal conductivity detector referencing standard gas with a known concentration of H_2_. Data are represented as mean ± SD from at least three repeated measurements.

### Molecular Dynamics (MD) Simulations

#### Model Setup

The TBAP-α model (1 row, 5 columns) was obtained from the published data,^35^ and the structure of the CsoS1A protein was downloaded from the Protein Data Bank (PDB ID: 2EWH).^44^ The CsoS1A was docked to the TBAP-α model using the HDOCK Server (http://hdock.phys.hust.edu.cn/).^51^ The structures with the highest scores were manually screened. The best-fit conformation was selected for molecular dynamic simulation using the AMBER20 software package.^52^ The ff14SB^53^ force field generated the protein parameters. The Generalized Amber Force field (GAFF) force field^54^ is used to create the force field of the TBAP molecule. The atomic charges of the TBAP molecule were calculated by the restricted electrostatic potential (RESP) method with B3LYP/6-31G(d)^55,56^ level of theory by the Gaussian 09 program. After examining the protonation state of the protein and adding hydrogen atoms, the system was solvated in a truncated octahedral box of the TIP3P water model^57^ with a buffer of 35.0 Å. Na^+^ and Cl^–^^58^ were added to neutralize the system and achieve a physiological salt concentration of 150 mM. The resulting system comprised 465,869 atoms.

#### MD Simulations

The system was minimized by 1000 steps of the fastest descent algorithm and 1000 steps of the conjugation gradient algorithm. Then, the system was heated to 300 K in 1 ns, and 300 ns of simulations were performed with 40 ns of NVT simulations and 260 ns of NPT simulation. The electrostatic interactions were calculated using the particle mesh Ewald (PME)^59^ method, and the periodic boundary conditions were used. The Langevin thermostat method^60^ is used to control the temperature, and the Berendsen barostat method^61^ was used to control the system’s pressure. To mimic the environment of carboxysomes, a restraint of 10 kcal mol^–1^ Å^–2^ was applied to the backbone of the protein and TBAP-α during the simulation. The trajectory of the last 30 ns was selected as a production run for analysis.

#### Analysis of Simulations

The root-mean-square deviation (RMSD) of the trajectory of molecular dynamics simulations is calculated by the RMSD Trajectory Tool in VMD.^62^ The hydrogen bond and salt bridge analysis used the PLIP web tool^57^ (https://plip-tool.biotec.tu-dresden.de/plip-web/plip/index). The maximum distance of the hydrogen bond was set to 4.1 Å, and the minimum angle of a hydrogen bond was 100°. The cutoff of the salt bridge interaction is 5.5 Å.

## Supporting Information Available

Chemical synthesis pathway of TBAP (Scheme S1); NMR spectrum of TBAP (Figures S1 and S2); FI-IR spectrum of TBAP (Figure S3); crystal model of TBAP-α and 3D model of α-carboxysome shell (Figure S4); PXRD patterns of TBAP phases, nitrogen adsorption isotherm and desorption isotherm for activated TBAP-α and amorphous TBAP, and SEM images of amorphous TBAP (Figure S5); solid UV–vis absorption spectrum of amorphous TBAP and TBAP-α (Figure S6); (α*h*ν)^1/2^ versus *h*ν curve and Mott–Schottky plot, diagram of conduction band and valence band of amorphous TBAP and TBAP-α, and cyclic voltammetry plot of TBAP-α (Figure S7); amino acid sequence alignment of α-carboxysome shell proteins (Figure S8); front and side views of the structures of α-carboxysome shell proteins (Figure S9); confocal microscopy images of *E. coli* cells expressing *mCherry-CsoS2*C (*mCherry-EP*) and coexpressing shells and *mCherry-csoS2C* (C–S) and SDS-PAGE of purified C–S (Figure S10); TEM image of C–S (Figure S11); SEM and confocal microscopy images of pyrene crystals and pyrene crystals with C–S (Figure S12); ζ-potentials of TBAP-α and α-carboxysome shell-encasing proteins (Figure S13); size distribution of H–S revealed by SEM (Figure S14); SDS-PAGE result of H–S purification (Figure S15); immunoblot analysis of purified H–S (Figure S16); colloidal stability of H–S|TBAP-α (Figure S17); photocurrent responses and EIS analysis for TBAP-α and H–S|TBAP-α (Figure S18); H_2_ production condition optimization of H–S|TBAP-α (Figure S19); photocurrent response and EIS analysis for amorphous TBAP and TBAP-α (Figure S20); wavelength-dependent AQY value of H–S|TBAP-α (Figure S21); H_2_ evolution of TBAP-α, H–S|TBAP-α, and 1 wt % Pt|TBAP-α (λ > 420 nm) as a function of time (Figure S22); cycling measurements for the photocatalytic hydrogen evolution of H–S|TBAP-α (Figure S23); immunoblot analysis of purified HydA (Fd-HydA-EP) (Figure S24); SEM images (Figure S25) and PXRD pattern (Figure S26) of H–S|TBAP-α after 30 h irradiation; gene maps of used plasmids (Figure S27); crystal data and structure refinement of TBAP-α (Table S1); protein components in recombinant α-carboxysomes from *E. coli* (Table S2); statistics of residues for TBAP-α and CsoS1A protein binding calculated by MD simulations (Tables S3 and S4); estimated fluorescence lifetimes of TBAP-α and H–S|TBAP-α (Table S5); a list of hydrogen evolution reaction conditions in this work (Table S6); a comparison of the H–S|TBAP-α assembly performance with the related state-of-the-art photocatalysts (Table S7); primers used for *pCDFDuet-mCherry-CS2* plasmid construction (Table S8); and gene sequences of used plasmids (Table S9). (PDF)
