# Supplementary material for: Light-Driven Hybrid Nanoreactor Harnessing the Synergy of Carboxysomes and Organic Frameworks for Efficient Hydrogen Production
Source: ACS Catal. 2024 Dec 6;14(24):18603–14. doi: 10.1021/acscatal.4c03672 (PMC11667666; doi:10.1021/acscatal.4c03672)
Supplement: Supplementary file 1 — cs4c03672_si_001.pdf [file cs4c03672_si_001.pdf]

Supporting Information

for

Light-driven hybrid nanoreactor harnessing the synergy of carboxysomes and organic frameworks for efficient hydrogen production

Jing Yang[1,2], Qiuyao Jiang[2], Yu Chen[2], Quan Wen[3], Xingwu Ge[2], Qiang Zhu[1], Wei Zhao[1], Oluwatobi Adegbite[2], Haofan Yang[1], Liang Luo[1], Hang Qu[1], Veronica Del-Angel-Hernandez[1], Rob Clowes[1], Jun Gao[3], Marc A. Little[1,5], Andrew I. Cooper[1]*, Lu-Ning Liu[2,4]**

*Correspondance: m.little@hw.ac.uk, aicooper@liverpool.ac.uk, luning.liu@liverpool.ac.uk

Supplmentary Information:

Supplmentary Methods

Supplementary Figures 1-27

Supplementary Tables 1-9

Supplementary References

SUPPLEMENTARY METHODS

1. Materials

All reagents were obtained from Sigma-Aldrich, TCI Europe or Carbosynth Ltd. Anhydrous solvents were purchased from Acros Organics or Fisher Scientific. All chemicals were used without further purification. All gases for sorption analysis were supplied by BOC at a purity of ≥ 99.999%. Reactions were carried out under an N_2 atmosphere using standard Schlenk techniques.

2. Synthesis and Characterization of 1,3,6,8-tetra(4′-carboxyphenyl)pyrene (TBAP)

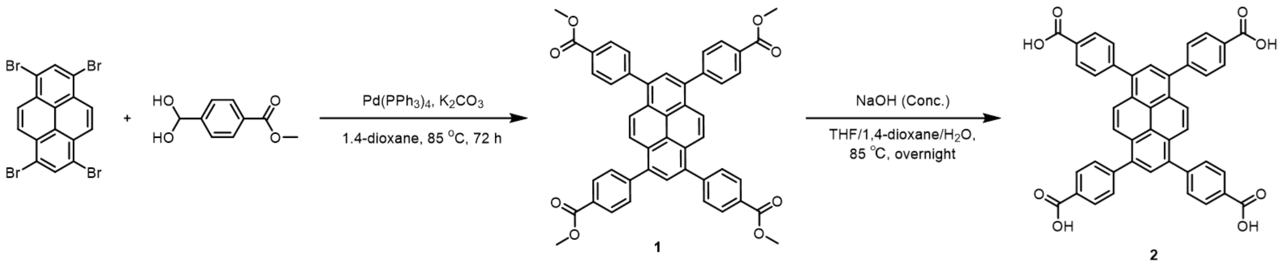

Scheme S1. Chemical synthesis of TBAP from 1,3,6,8-tetrabromopyrene. 1, TBAP-Me. 2, TBAP.

2.1 Synthesis of 1,3,6,8-Tetrakis(4-(methoxycarbonyl)phenyl)pyrene (TBAP-Me):

TBAP-Me was synthesized according to a previously reported procedure[1]. A mixture of (4-(methoxycarbonyl)phenyl)boronic acid (5 g, 32.9 mmol, 6 eq.), 1,3,6,8-tetrabromopyrene (2.85 g, 5.5 mmol), Pd tetrakis(triphenylphosphine) (0.1 g, 0.09 mmol, 1.6 mol%), and potassium carbonate (6 g, 44 mmol, 8 eq.) in dry dioxane (50 mL) was stirred under nitrogen for 3 days at 85 °C. The reaction mixture was poured into a water and ice solution containing 1 M aqueous hydrochloric acid solution. The organic phase was extracted with chloroform, the extract was dried over magnesium sulfate, and the solvent volume was reduced under vacuum. The residue was purified by a short silica column eluent with chloroform to remove Pd and afford 1,3,6,8-tetrakis(4-(methoxycarbonyl)phenyl)pyrene in an 80% yield: 3.29 g (4.4 mmol). ^{1}H-NMR (400 MHz, $CDCl_3$): δ 8.32–8.20 (m, 8H), 8.16 (d, J_{HH} = 10.9 Hz, 4H), 8.07–7.98 (m, 2H), 7.76 (t, J_{HH} = 9.9 Hz, 8H), 4.02 (s, 12H). The NMR data for 1 is consistent with literature-reported values[1].

2.2 Synthesis of TBAP:

A concentrated sodium hydroxide aqueous solution (20 mL) portion was added to a suspension of TBAP-Me (1 g) in THF/dioxane/H_2O (100 mL, ratio 5/2/2), and the mixture was stirred under reflux at 85 °C overnight. The solvent was removed under vacuum, and H_2O was added to the residue. The mixture (clear yellow solution) was stirred at room temperature for 2 h. The pH value was adjusted to 2 using concentrated HCl. The resulting yellow solid was collected by filtration, washed with water, 1 M HCl aqueous solution, and diethyl ether, and then dried under vacuum to afford TBAP in a 97% yield: 0.88 g (1.29 mmol). ^{1}H NMR (DMSO-*d*$_6$): δ 7.83 (d, *J* = 8.4 Hz, 8H), 8.06 (s, 2H), 8.14 (d, *J* = 8.4 Hz, 8H), 8.18 (s, 4H), 13.1 (s, 4H). ^{13}C NMR (DMSO-*d*$_6$): δ 167.7, 144.6, 136.7, 131.2, 130.7, 130.1, 129.9, 128.1, 125.9, 125.6. The NMR data for TBAP is consistent with literature-reported values[1].

3. Characterization Methods

3.1 Solution nuclear magnetic resonance (NMR)

^1H and ^{13}C NMR spectra were recorded in solution at 400 MHz and 100 MHz, respectively, using a Bruker Avance 400 NMR spectrometer.

3.2 Powder X-ray diffraction (PXRD)

PXRD patterns were collected in transmission mode on samples held on thin Mylar film in aluminium well plates on a Panalytical Empyrean diffractometer, equipped with a high throughput screening (HTS) XYZ stage, X-ray focusing mirror, and PIXcel detector, using Cu-Kα (λ = 1.541 Å) radiation. Diffraction patterns were measured over the 2θ range, 1–56°, in 0.013° steps for 60 minutes.

3.3 Gas sorption analysis

Surface areas were measured by nitrogen sorption at 77.3 K. Powder samples were degassed offline at 120°C for 15 h, followed by degassing on the analysis port under vacuum, also at 100°C. Isotherm measurements were performed using a Micromeritics 3flex surface characterization analyzer, equipped with a Cold-Edge technologies liquid helium cryostat chiller unit for temperature control. For TBAP-α, BET surface areas were calculated over the relative pressure range 0.02–0.06.

3.4 Scanning electron microscopy (SEM)

The morphologies of crystals and bio-hybrid nanoreactors were imaged using a Hitachi S-4800 cold field emission scanning electron microscope (FE-SEM). Samples were prepared by dropping 10 μL dispersed solution on 15 mm Hitachi M4 aluminium stubs using an adhesive high-purity carbon tab before coating with a 2 nm layer of gold using an Emitech K550X automated sputter coater. Imaging was conducted at a working voltage of 3-5 kV and a working distance of 8 mm using a combination of upper and lower secondary electron detectors.

3.5 Transmission electron microscopy (TEM)

TEM images were obtained on a JEOL 2100FCs microscopy at an accelerating voltage of 200 kV. TEM samples were prepared by depositing 3 μL protein solution on the copper grid for several minutes. Then, the solution was blotted away, and 3 μL 1 wt.% uranyl acetate solution was dropped to stain the samples for about 40 s. TEM samples were analyzed by ImageJ software. More than 150 particles were counted to obtain the size distribution.

3.6 Photoluminescence (PL) spectra and time-correlated single photon counting (TCSPC) measurements

Photoluminescence spectra were performed on a Shimadzu RF-5301PC fluorescence spectrometer. Time-correlated single photon counting (TCSPC) experiments were measured on an Edinburgh Instruments LS980-D2S2-STM spectrometer (EPL-375 diode, λ = 371 nm). The instrument response of the TCSPC spectrometer

was measured with colloidal silica (LUDOX HS-40, Sigma-Aldrich) at the excitation wavelength. Decay times were fitted in the FAST software using three decay exponents. Samples for PL spectrum and TCSPC measurements were prepared under anaerobic conditions.

3.7 UV-Visible spectrum (UV-Vis)

The colloidal stability of H-S|TBAP-α was monitored on a Cary 5000 UV-visible-NIR spectrophotometer. The absorption spectra of H-S|TBAP-α were detected at 0, 0.5, 1, 1.5, 2, 3 and 19 h, respectively.

3.8 Zeta potential (ZP)

Zeta Potential (ZP) measurements were performed in water on a Malvern Zetasizer Nano Particle Sizer, at 25°C. Data were obtained from 15 measurement cycles and repeated three times.

3.9 Confocal laser scanning microscopy (CLSM)

Overnight-induced *E. coli* cells or TBAP-α and C-S|TBAP-α dispersed solution were immobilized by drying a droplet of cell suspension onto LB agar pads. Blocks of agar with the cells absorbed onto the surface were covered with a coverslip and placed under the microscope. Laser-scanning confocal fluorescence microscopy imaging was performed on a Zeiss LSM780 confocal microscope with a 63×/1.4 NA oil-immersion objective with excitation wavelength at 488 nm and emission at 520 nm. Live-cell images were recorded from at least five different cultures. All images were captured with all pixels below saturation. Image analysis was carried out using ImageJ software (version 1.52 h).

3.10 Isothermal titration calorimetry (ITC)

ITC experiments were carried out with Malvern Microcal Peaq Automated fitted with its proprietary software (MicroCal PEAQ_ITC Automated Control Software). The instrument was set for 19 injections at 25 °C while reference power, initial delay, and stirring speed were set for 6 µcal/s, 60 s and 750 rpm, respectively. The cell contained 5 µM of carboxysome shell (6.5 MDa), while the syringe contained the ligand at a concentration of 50 µM. Both carboxysome and ligand were diluted in buffers containing 20% sucrose and TN buffer. The start injection volume was 0.4 µL while subsequent injections were 2 µL. MicroCal PEAQ-ITC Analysis Software was used for data evaluation.

3.11 Photoelectrochemical (PEC) measurements

Photoelectrochemical experiments were performed on the BioLogic SP200 workstation. The transient photocurrent responses (*I–t*) and electrochemical impedance spectra (EIS) of samples were investigated in a three-electrode system (FTO, Pt plate, and Ag/AgCl as working, counter, and reference electrode, respectively) using a 300 W Xe light source (Newport) with 420 nm filter. The electrolyte was 0.05 M 2-(*N*-Morpholino) ethane sulfonic acid (MES, pH 6) aqueous solution and was degassed for 30 minutes using N_2 at room temperature before the measurement. The working electrode of TBAP-α was prepared as follows: 2 mg of ground TBAP-α was mixed with 10 µL Nafion (5 wt.%) in 500 µL TN buffer, giving a slurry. 10 µL of the

above slurry was then coated onto FTO glass electrodes with an active area of 1 cm^2. The working electrode of H-S|TBAP-α was prepared as follows: 2 mg of ground TBAP-α and 0.5 mL H-S in TN buffer (1 mg/mL of total protein, measured by Nanodrop method) was mixed with 10 μL Nafion (5 wt.%) under anaerobic condition. 10 μL slurry of H-S|TBAP-α was then coated onto FTO glass electrodes with an active area of 1 cm^2 under anaerobic conditions. The applied bias for intermittent photocurrent intensity measurement was 0.4 V vs Ag/AgCl. The EIS spectra were determined by applying a 10 mV AC signal over the frequency range of 100 kHz to 10 mHz at a DC bias of 0.4 V vs Ag/AgCl.

3.12 Sodium dodecyl-sulfate polyacrylamide gel electrophoresis (SDS-PAGE) and immunoblot analysis

SDS-PAGE and immunoblot analysis samples were prepared by mixing protein samples with 4 x Loading Buffer and heating at 100 °C for 10 minutes. Then, 10 μL of each sample was loaded into each well on 15% (v/v) denaturing SDS-PAGE gels and electrophoresed for 45-90 minutes. SDS-PAGE results were analyzed after staining and destaining. For immunoblot analysis, gels were electroblotted onto a PVDF membrane (Bio-Rad) and treated with the membrane mouse monoclonal anti-His (Invitrogen, Catalog # 4E3D10H2/E3, dilution 1:5000) and horseradish peroxidase-conjugated goat anti-mouse IgG secondary antibody (Invitrogen, Catalog # C163, dilution 1:10,000). Signals were visualized using a chemiluminescence kit (Bio-Rad). Immunoblot images were collected using ImageQuant LAS 4000 software version 1.2.1.119.

3.13 Apparent quantum yield (AQY) measurement

The apparent quantum yield (AQY) was determined under monochromatic LED light irradiation at a certain wavelength (λ = 420 nm, 470 nm or 490 nm), and the light intensity was measured by a ThorLabs PM100D Power with a photodiode sensor. The AQY was calculated using the following equation:

$$AQY\ \% = \frac{[\text{hydrogen produced (mol)}] \times 2}{\text{photon number entered into the reactor (mol)}} \times 100$$
$$= \frac{[\text{Na} \times \text{h} \times \text{c}][\text{hydrogen produced (mol)}] \times 2}{\text{I} \times \text{S} \times \text{t} \times \lambda} \times 100$$

Where, Na is Avogadro's constant (6.022 × 1023 mol^{-1}), h is the Planck constant (6.626 × 10^{-34} J s), c is the speed of light (3 × 108 m s^{-1}), S is the irradiation area (cm^2), I is the intensity of irradiation light (W cm^{-2}), t is the photoreaction time (s), λ is the wavelength of the monochromatic light (m).

3.14 Cyclic voltammetry (CV)

Cyclic voltammetry was performed in a three-electrode one compartment cell configuration with Pt plate as the counter electrode, Fluorine-doped Tin Oxide (FTO) electrode as working electrode and Ag/AgCl 3M KCl as reference on the BioLogic SP200 workstation. Electrolytic solutions (0.1 M Na_2SO_4 aqueous solution) were degassed under nitrogen for 30 min prior to measurements with fresh volumes (40 mL) being used for each experiment. The working electrode was prepared by adding 2.0 mg of TBAP-α in 0.5 mL of ethanol and 10

μL of Nafion resin solution and sonicated for 15 min to ensure a homogeneous suspension. The ink was drop-cast onto an FTO glass electrode with an active area of 1 cm^2. For the illuminated CV measurements, a 300 W Xe lamp equipped with a 420 nm filter was used as the light source. Potentials were pH corrected following:

$$E_{(RHE)} = E_{Ag/AgCl} + 0.059\ pH + E^{o}_{Ag/AgCl}$$

3.15 Cycle runs experiment

10 mg TBAP-α and 22.5 mL neutralised AA aqueous solution (0.1 M) were added to the 67 Ml quartz flask and purged with N_2 for 30 minutes followed by the addition of purified H-S solution (2.5 mL in 20% sucrose, 1 mg mL^{-1} measured using the Nanodrop method) under N_2 gas flow to make the final volume of 25 mL. The reaction mixture was illuminated with a 300 W Newport Xe light source (Model: 6258, Ozone-free) using a λ > 420 nm cut-off filter. The light source was cooled by water circulating through a metal jacket. Gas samples were taken with a gas-tight syringe and run on a Bruker 450-GC gas chromatograph. H_2 was detected with a thermal conductivity detector referencing against standard gas with a known concentration of H_2. The solution was purged with N_2 for 15 minutes per 5 h. The cycle experiment was carried out for 5 runs.

3.16. Mott-Schottky measurement

The Mott-Schottky plots were performed on the BioLogic SP200 workstation in a standard three-electrode system (FTO, Pt plate, and saturated Ag/AgCl as working, counter, and reference electrode, respectively) with 0.1 M Na_2SO_4 (pH = 7) as the electrolyte. The working electrode was prepared by adding 2.0 mg of amorphous TBAP or TBAP-α in 0.5 mL of ethanol and 10 μL of Nafion resin solution and sonicated for 15 minutes to ensure a homogeneous suspension. The ink was drop-cast onto an FTO glass electrode with an active area of 1 cm^2.

SUPPLEMENTARY FIGURES

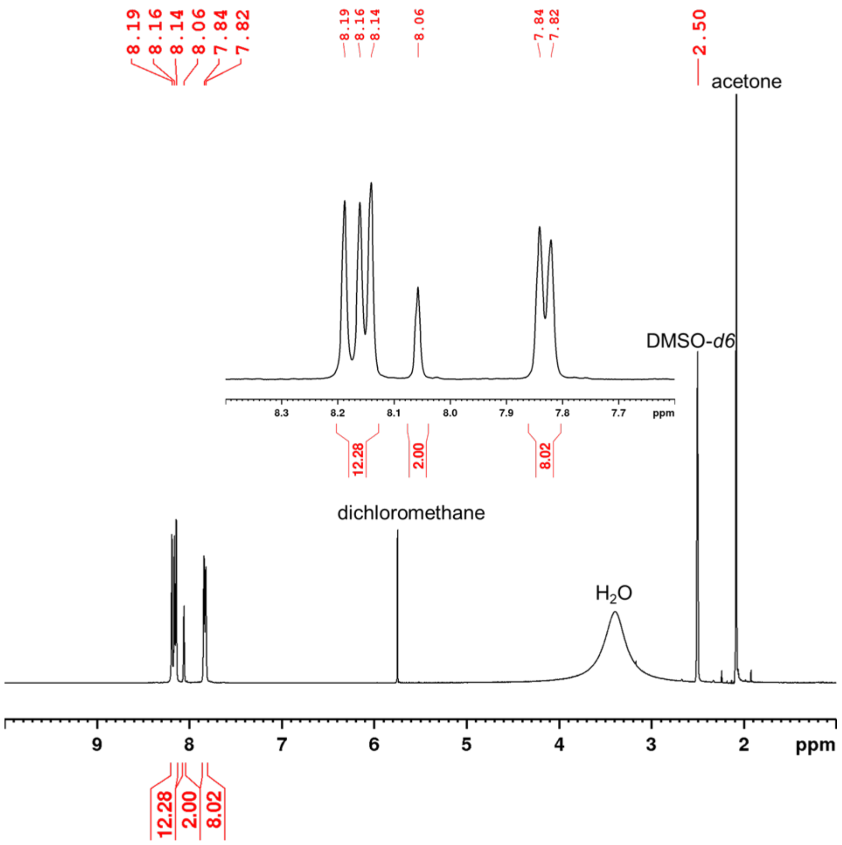

Figure S1. ^{1}H NMR (400 MHz, DMSO-*d6*) spectrum of TBAP. Inset, the splitting of the aromatic signals.

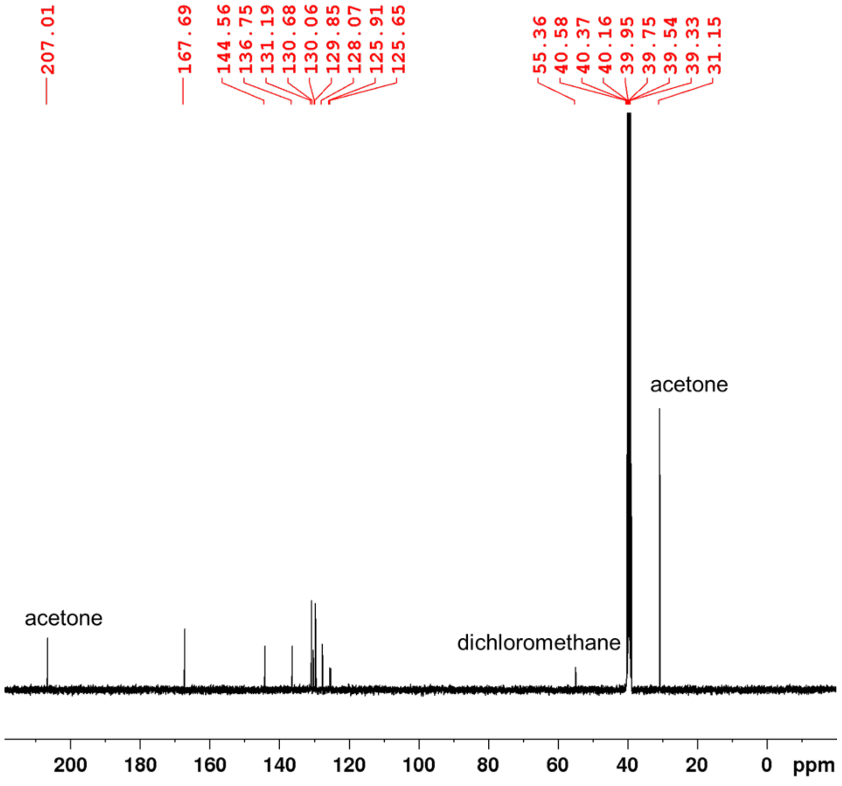

Figure S2. ^{13}C NMR (100 MHz, DMSO-*d$_6$*) spectrum of TBAP.

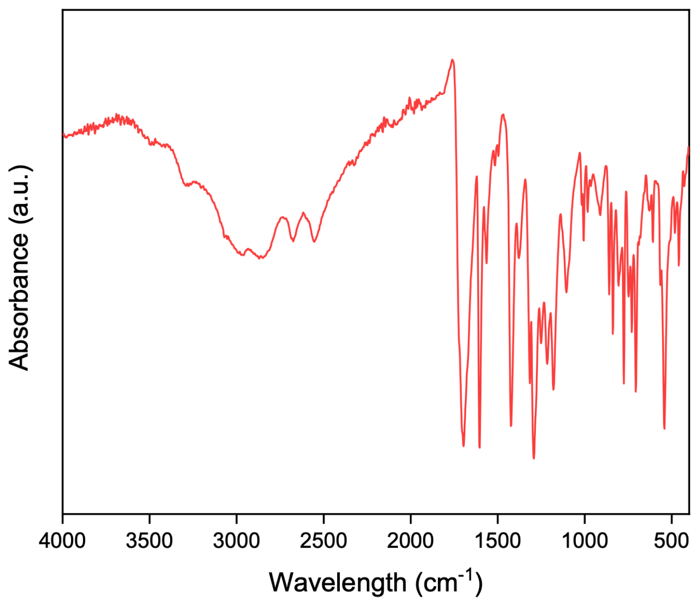

Figure S3. FT-TR spectrum of amorphous TBAP.

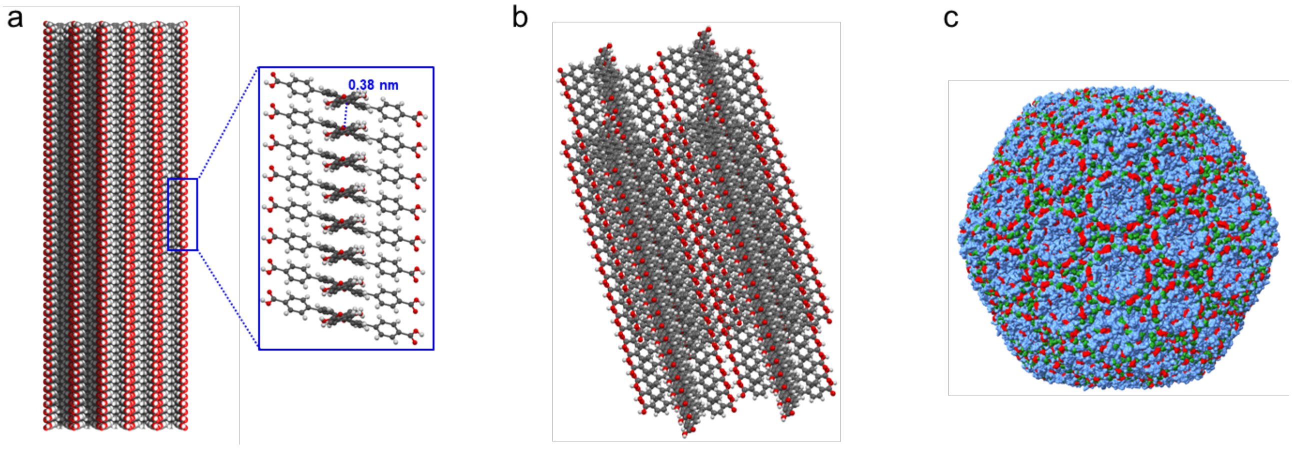

Figure S4. (a) and (b) Crystal model of TBAP-α (model created by Materials Studio, where grey = C atoms, white = H atoms, red = O atoms). (c) 3D model of α-carboxysome shell (PDB: 7CKC) viewed by ChimeraX. On the outer surface of the shell, red moieties represent arginine residues, and green moieties represent glutamic acid residues.

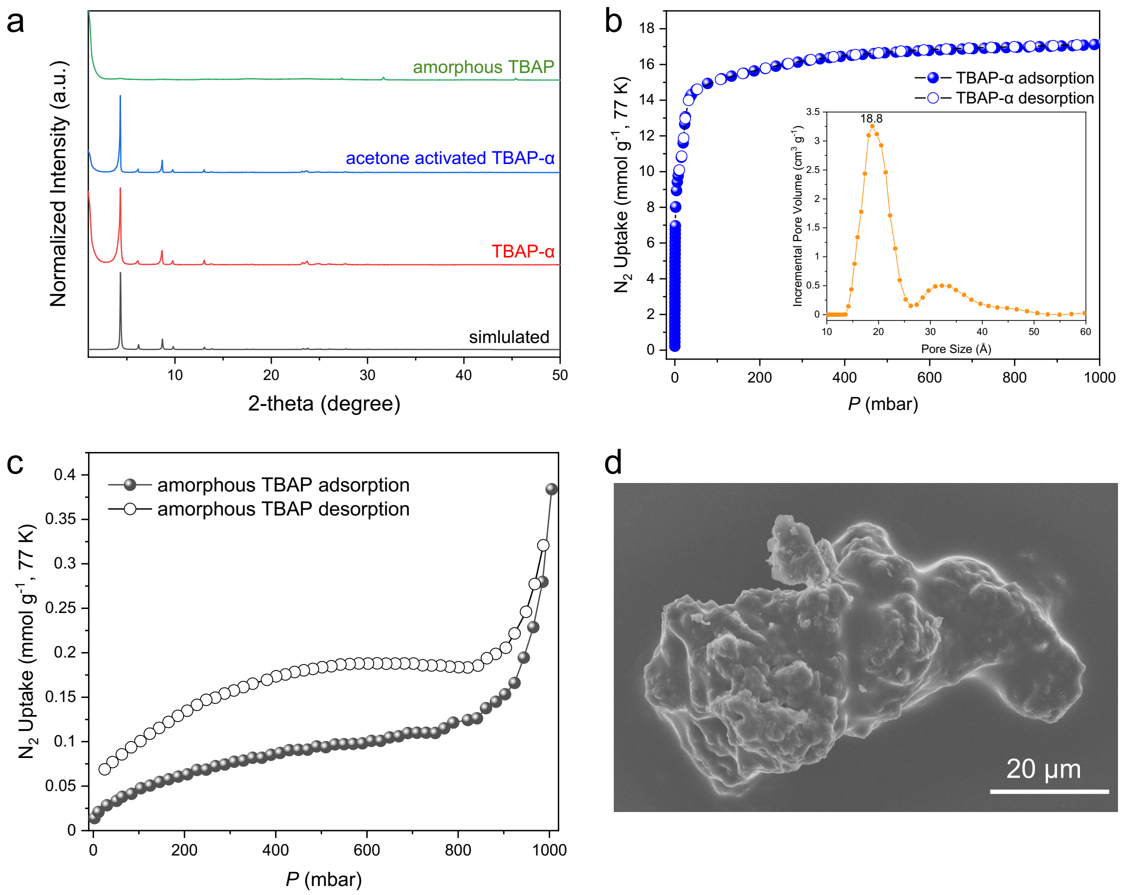

Figure S5. (a) PXRD patterns of TBAP phases: amorphous TBAP powder from synthesis (green line), activated TBAP-α used for gas sorption analysis and to construct H-S|TBAP-α (blue line), TBAP-α crystallised of DMF/$CHCl_3$ (red line), and simulated pattern of TBAP-α single crystal structure (black line). (b) Nitrogen adsorption isotherm (filled symbols) and desorption isotherm (open symbols) for activated TBAP-α recorded at 77.3 K. Inset, the pore size distribution plot. (c) Nitrogen adsorption isotherm (filled symbols) and desorption isotherm (open symbols) for amorphous TBAP recorded at 77.3 K. (d) SEM image as-synthesized amorphous TBAP.

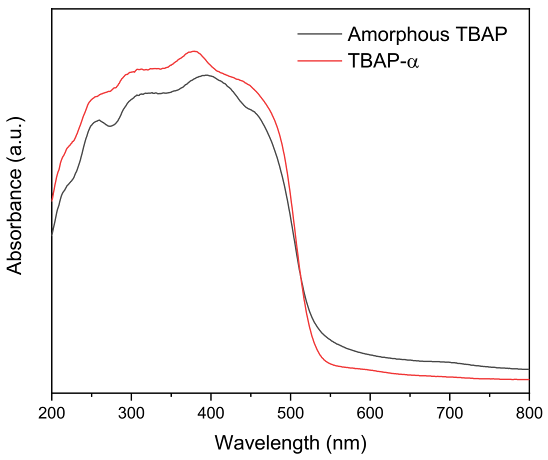

Figure S6. Solid UV-Vis absorption spectrum of amorphous TBAP (black line) and TBAP-α (red line).

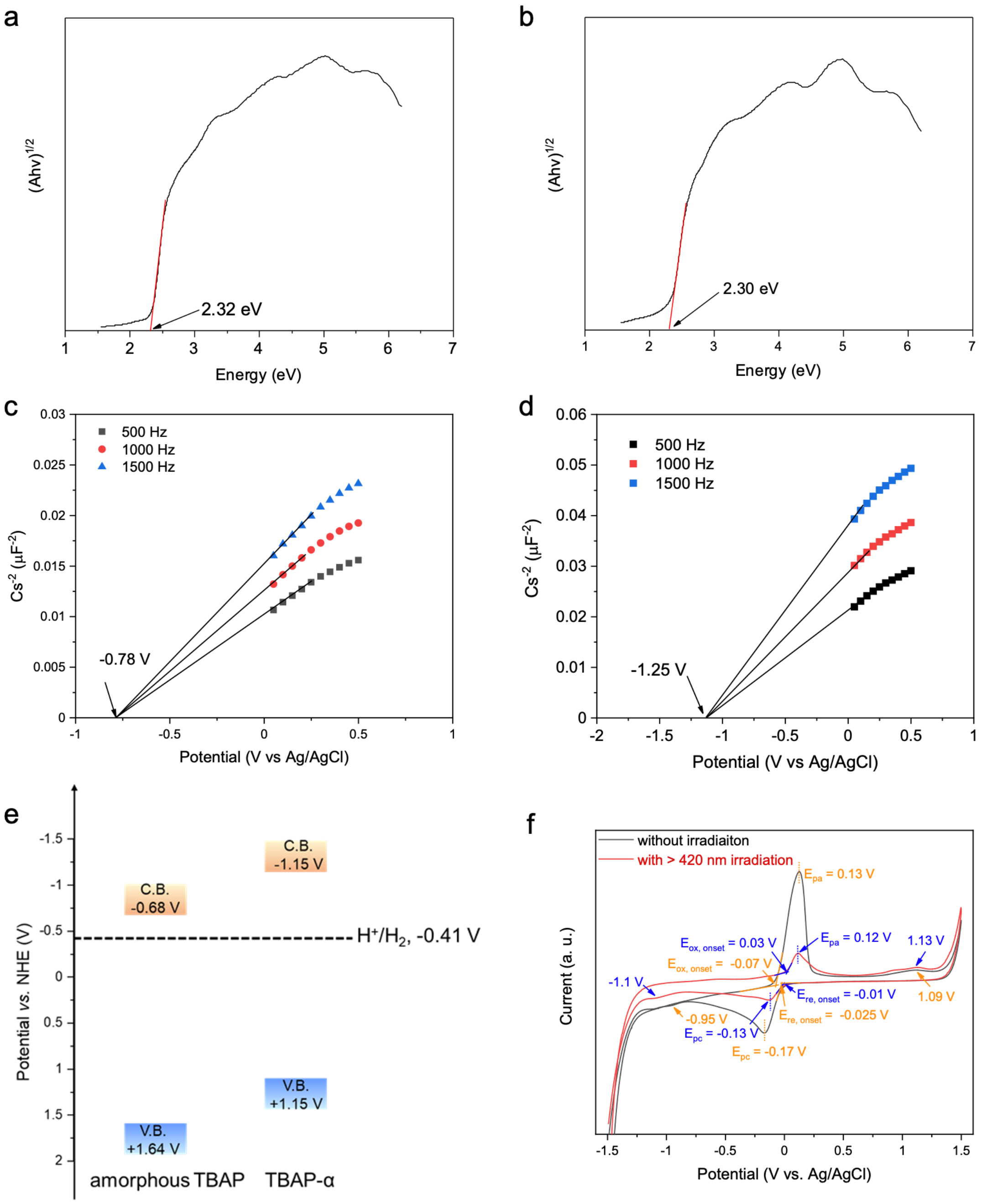

Figure S7. (a) and (b), $(\alpha h\nu)^{1/2}$ versus $h\nu$ curve of amorphous TBAP and TBAP-α (α, h, and ν are the absorption coefficient, Planck's constant, and light frequency, respectively). The intersection value between the tangent and the baseline is the bandgap (E_g) as 2.32 eV and 2.30 eV, respectively. (c) and (d), Mott-Schottky plot of amorphous TBAP and TBAP-α. The flat band (E_{fb}) potential of amorphous TBAP and TBAP-α were measured using the electrochemical Mott-Schottky technique on FTO glass in a standard three-electrode system, with 0.1 M Na_2SO_4 aqueous solution (pH = 7) as the electrolyte[2]. The E_{fb} values were +0.78 V for amorphous TBAP and -1.25 V for TBAP-α, which were then converted to -0.58 V vs. NHE for amorphous TBAP and -1.05 V vs. NHE for TBAP-α using the equation of $E_{NHE} = E_{Ag/AgCl} + 0.197$ V[3,4]. In addition, the positive slope indicated that amorphous TBAP and TBAP-α were n-type semiconductors. (e) Diagram of the conduction bands (E_{CB}) and valence bands (E_{VB}) of amorphous TBAP and TBAP-α. For many n-type semiconductors, E_{fb} is normally considered to be approximately 0.1 V more positive than their E_{CB}[3]. Therefore, E_{CB} was calculated to be -0.68 V vs. NHE for amorphous TBAP and -1.15 V vs. NHE for TBAP-α (Figure S7e). E_{VB} was calculated at 1.64 V vs. NHE for amorphous TBAP and 1.15 V vs. NHE for TBAP-α, using the equation of $E_{VB} = E_{CB} + E_g$. (f) Cyclic voltammetry plots of TBAP-α under dark (black line) and illuminated (300W Xe lamp with 420 nm filter, red line) conditions were measured using the suspension of TBAP-α in water deposited on FTO glass in 0.1 M Na_2SO_4 aqueous solution at the scan rate of 100 mV s^{-1}, taking the Pt plate as the counter electrode and Ag/AgCl (3M KCl) as reference. $E_{ox,\ onset}$ and $E_{re,\ onset}$ refer to the onset oxidation potential and onset reduction potential, respectively. E_{pa} and E_{pc} are the anodic peak potential and cathodic peak potential, respectively.

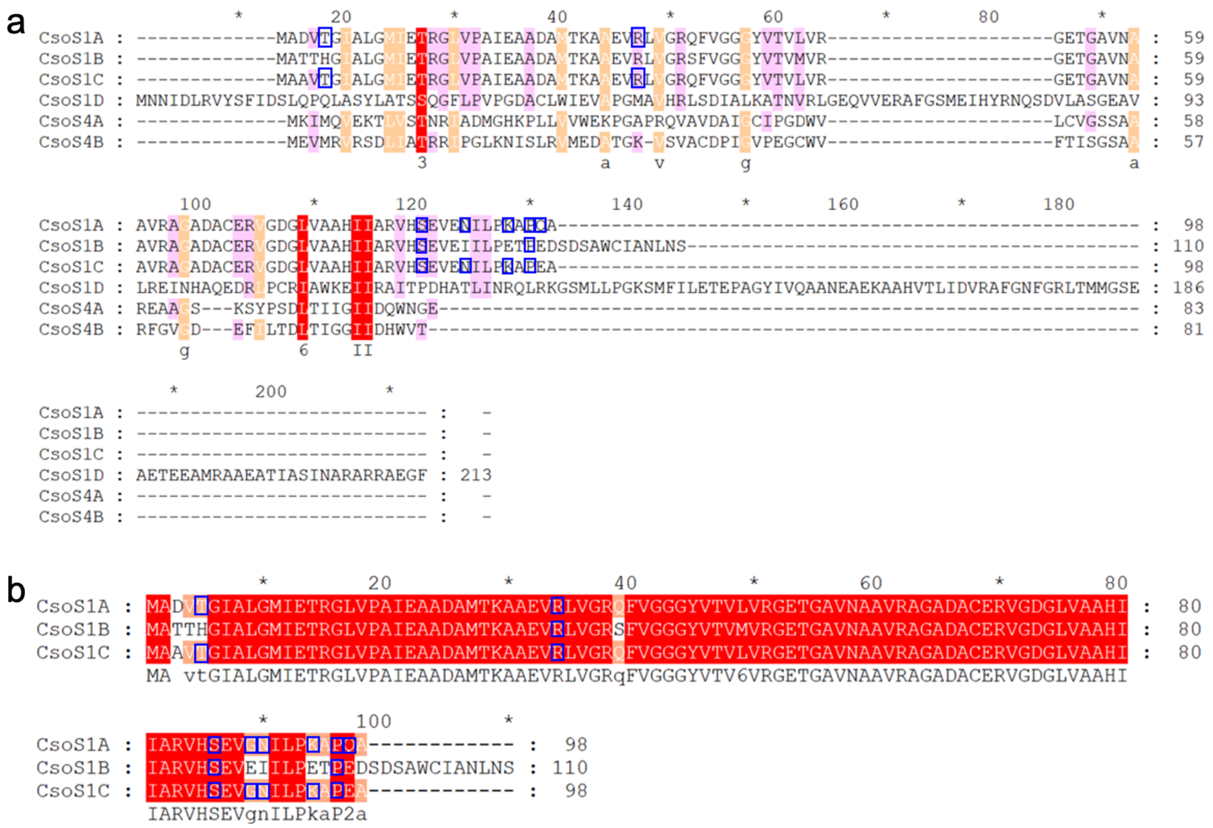

Figure S8. Amino acid sequence alignment of α-carboxysome shell proteins (a) CsoS1A, CsoS1B, CsoS1C, CsoS1D, CsoS4A, and CsoS4B; (b) the main hexamer shell proteins: CsoS1A, CsoS1B and CsoS1C. Highlight in red: conserved sequences (up to 100%); highlight in orange: highly conserved sequences (up to 80%), highlight in pink: moderately conserved sequences (up to 60%). blue box: suggested amino acids from MD simulations.

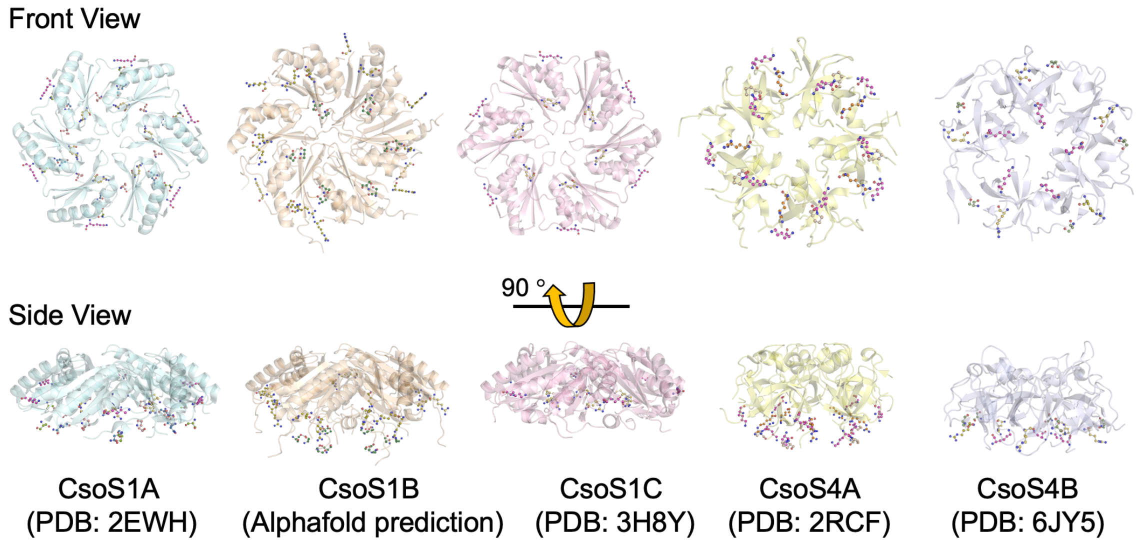

Figure S9. Front (top) and side (bottom) views of the structures of α-carboxysome shell proteins (CsoS1A, CsoS1B, CsoS1C, CsoS4A, CsoS4B). Ball-and-stick models indicate the Thr5, Arg34, Ser86, Gly89, Asp90, Lys94, Pro96, and Glu97 residues that bind with TBAP-α.

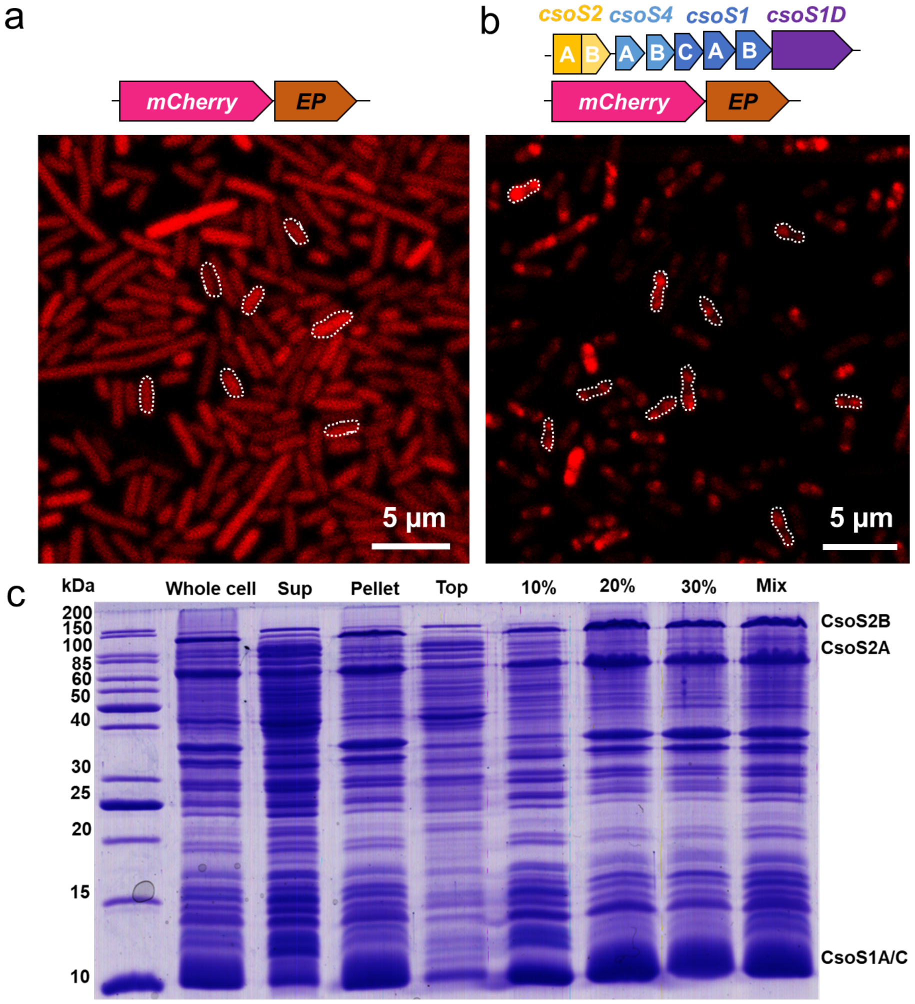

Figure S10. Confocal images of *E. coli* cells expressing (a) *pCDFDuet-mCherry-CsoS2* (mCherry-EP), (b) co-expressing *pCDFDuet-mCherry-CsoS2* and *pBAD-cso-2* (shell) (C-S). (c) SDS-PAGE of mCherry-EP-Shell (C-S) after co-expression and purification by sucrose gradient ultracentrifugation. The samples in the panels are (from left to right): whole cell, supernatant after 50,000g centrifugation, pellet after 50,000g centrifugation, top fraction and 10% to 30% sucrose fraction after sugar gradient ultracentrifugation, respectively. The corresponding expressed protein names are in brackets.

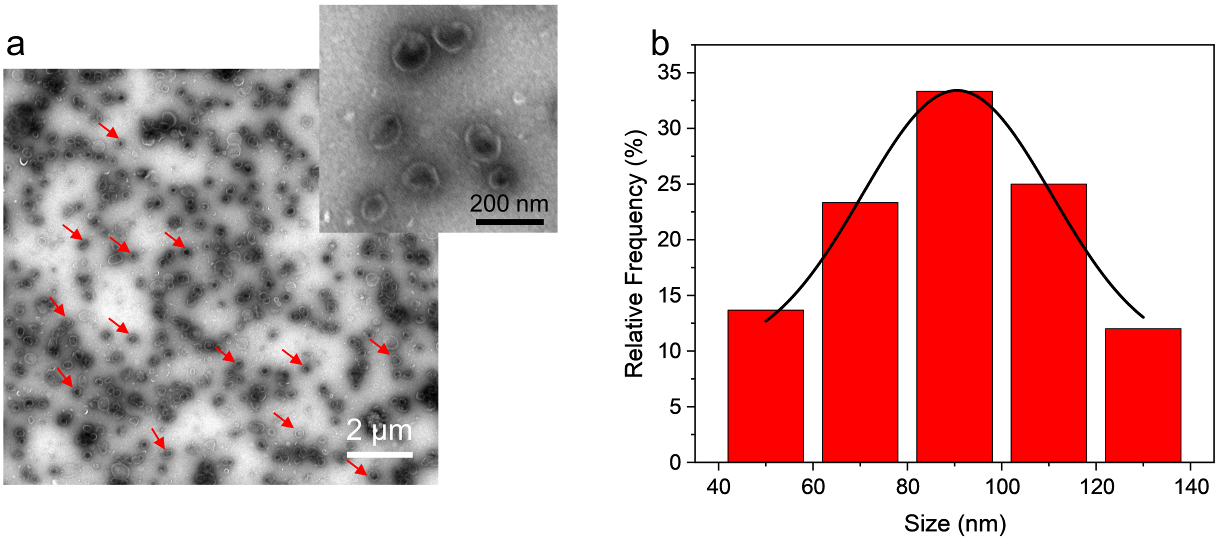

Figure S11. (a) TEM image of mCherry-EP-Shell (C-S) (inset, zoom-in view). (b) Size distribution of C-S in 20% sucrose fraction after purification by sucrose gradient ultracentrifugation, analysed by ImageJ.

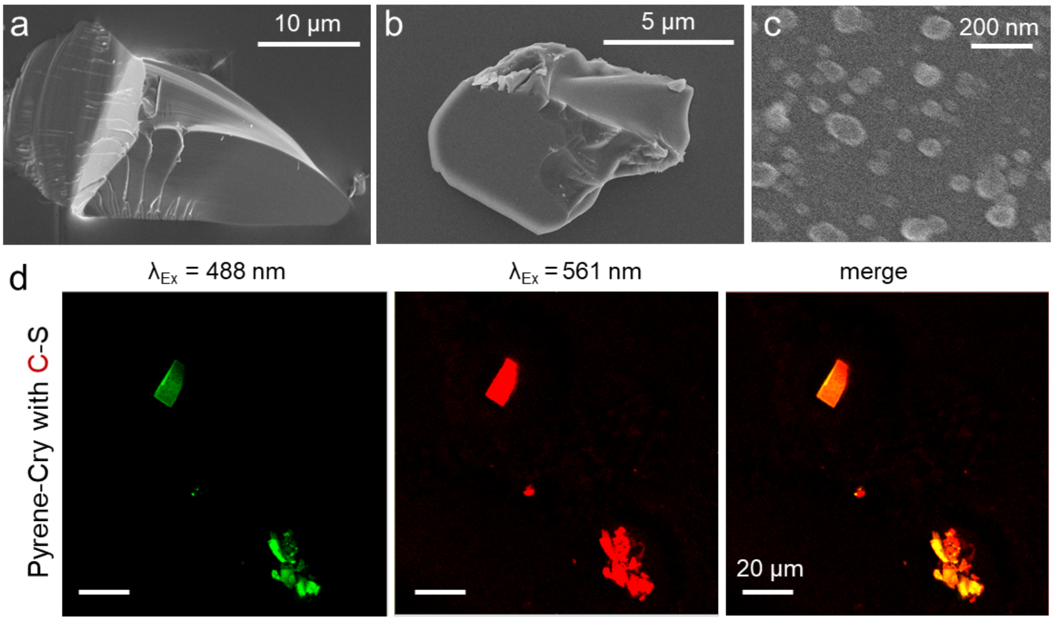

Figure S12. Scanning electron microscopy images of (a) bulk pyrene crystals grown from acetone, (b) a mixture of C-S and pyrene crystals, and (c) nanoparticles in the solution of C-S and pyrene crystals mixture. (d) Confocal microscopy images of pyrene crystals with C-S when excited at 488 and 561 nm. All fluorescence images were adjusted to have the same brightness and contrast settings.

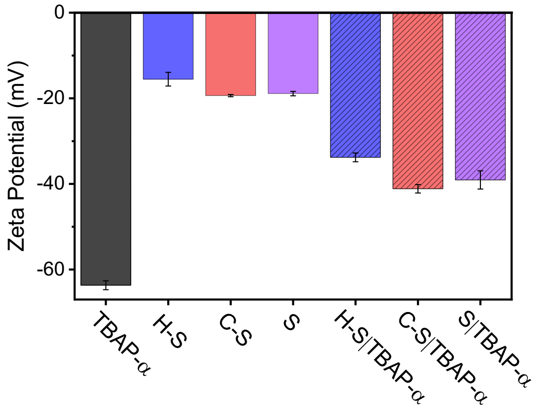

Figure S13. Zeta potentials of TBAP-α, H-S (α-carboxysome shell encapsulating [FeFe]-hydrogenases), C-S (α-carboxysome shell encapsulating mCherry), and S (α-carboxysome shell), as well as their hybrid forms associated with TBAP-α, including H-S|TBAP-α, C-S|TBAP-α, and S|TBAP-α. Error bars represent the standard deviation of the mean of three independent experiments.

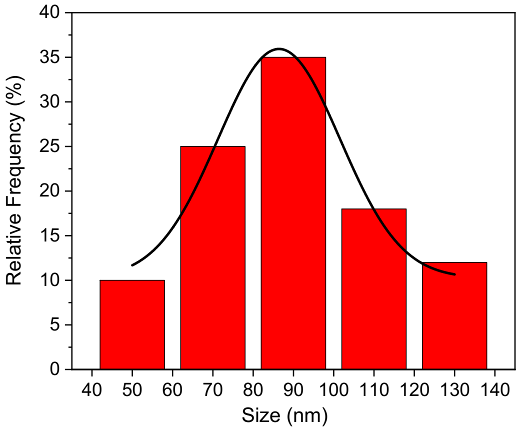

Figure S14. Size distribution of H-S revealed by SEM.

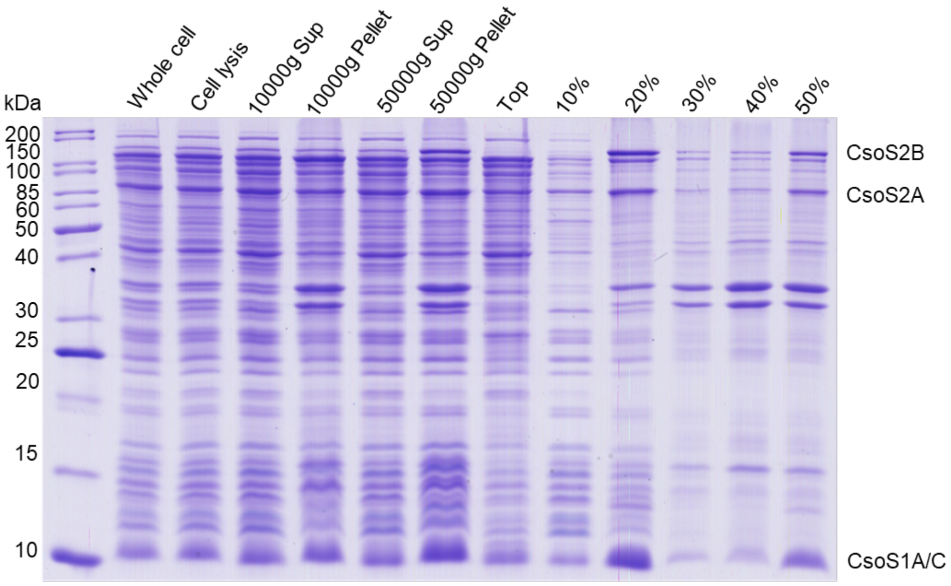

Figure S15. SDS-PAGE result of H-S after co-expression and purification by sucrose gradient ultracentrifugation. The samples in the panels are (from left to right): whole cell, after cell lysis by sonication, supernatant after 10,000g centrifugation, pellet after 10,000g centrifugation, supernatant after 50,000g centrifugation, pellet after 50,000g centrifugation, top fraction and 10% to 50% sucrose fraction after sucrose gradient ultracentrifugation, respectively.

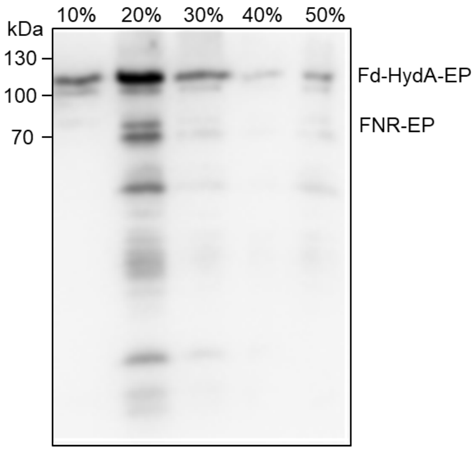

Figure S16. Immunoblot analysis of purified H-S in the 10-50% sucrose fractions confirms the presence of Fd-HydA and FNR in the nanoreactors using 6×His antibody.

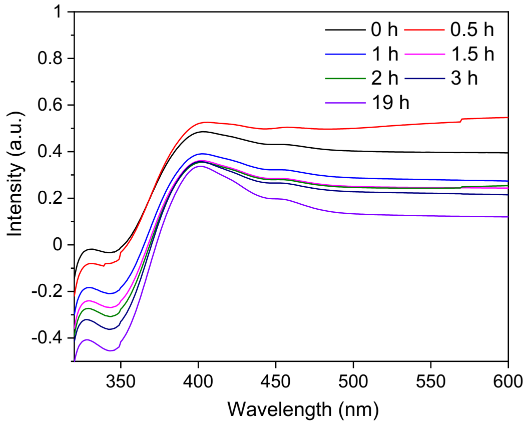

Figure S17. Colloidal stability of H-S|TBAP-α in 0.1 M neutralised AA aqueous solution monitored over time by UV-Vis spectroscopy.

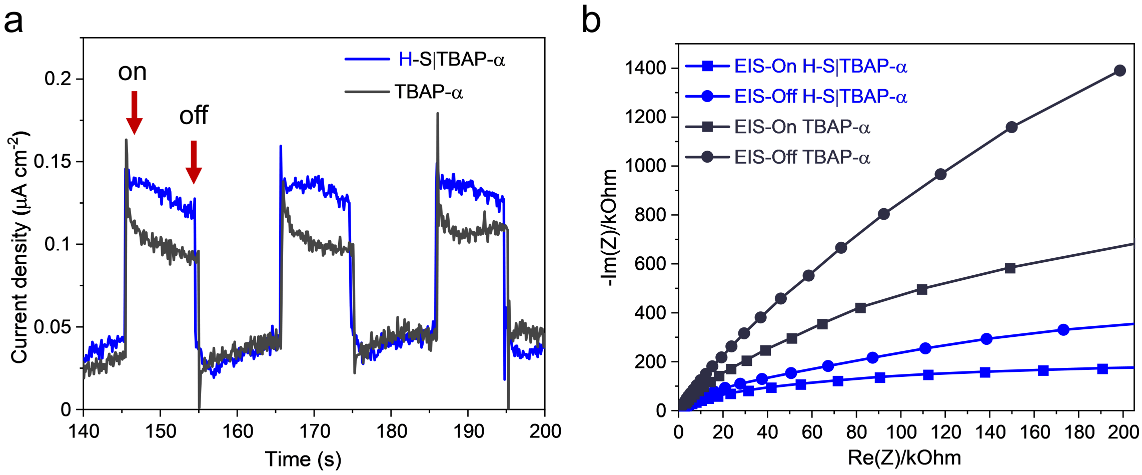

Figure S18. (a) Photocurrent responses, (b) EIS analysis for TBAP-α and H-S|TBAP-α with applied potentials at 0.4 V vs. Ag/AgCl under intermittent irradiation (300 W Xe light source with λ > 420 nm filter).

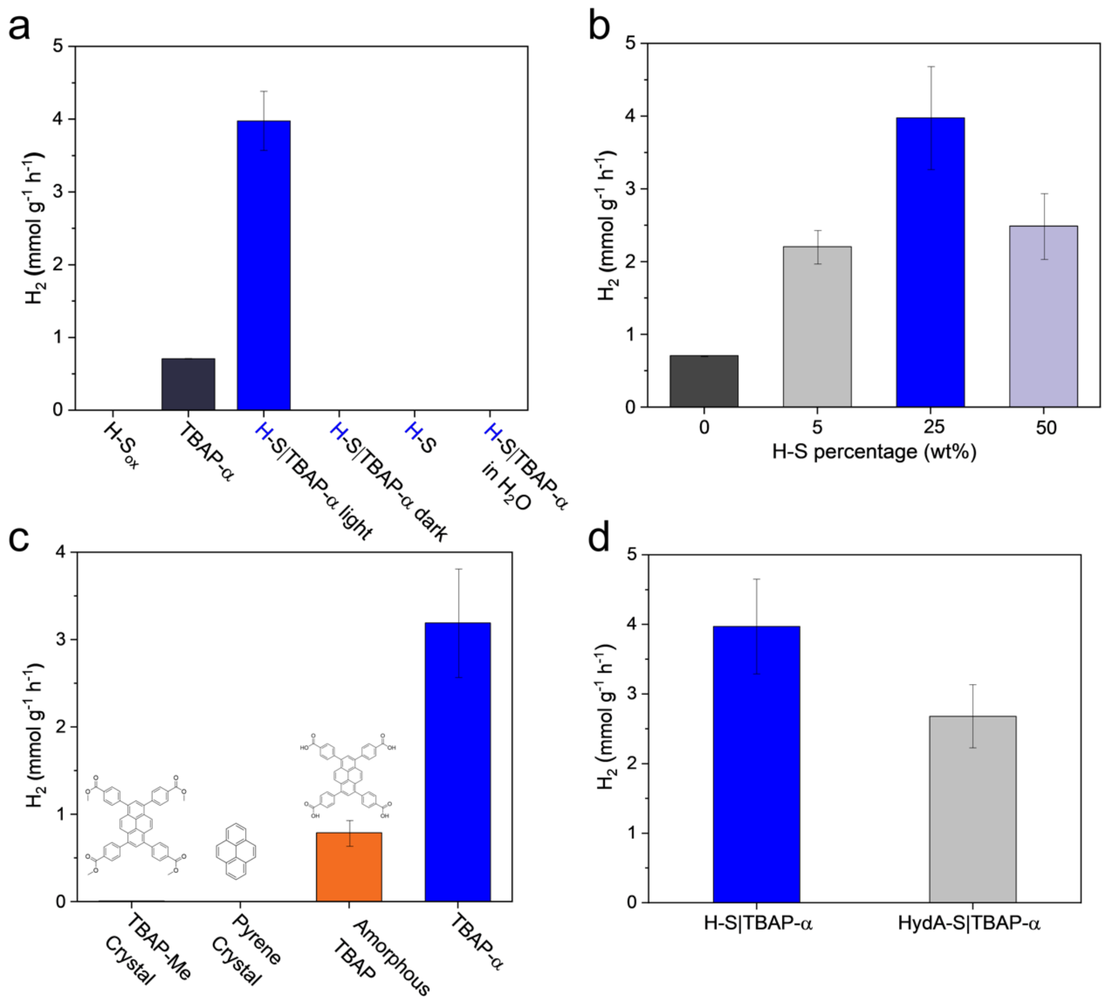

Figure S19. (a) H_2 evolution activity of H-S_{ox} detected by sodium dithionite-methyl viologen assay (H-S_{ox}); TBAP-α in 0.1 M neutralised AA aqueous solution (TBAP-α); H-S|TBAP-α in 0.1 M neutralised AA aqueous solution after solar simulator irradiation (H-S|TBAP-α light); H-S|TBAP-α in 0.1 M neutralised AA aqueous solution after being stored in the dark (H-S|TBAP-α dark), H-S|TBAP-α in water after solar simulator irradiation (H-S|TBAP-α in water). (b) The effect of H-S concentration on H-S|TBAP-α H_2 evolution activity. The protein concentration was adjusted to 1 mg mL^{-1}, measured by the Nanodrop method. (c) Photocatalytic activity of pyrene-based crystalline materials including TBAP-Me and pyrene, as well as amorphous TBAP and TBAP-α with H-S, respectively. (d) H_2-evolution activity of H-S|TBAP-α and HydA-S|TBAP-α in 0.1 M neutralised AA aqueous solution. For all the measurements, 2 mg of chemical components were dispersed in 4.5 mL 0.1 M neutralised AA aqueous solution or 4.5 mL water for H-S|TBAP-α in water. These solutions were degassed with N_2 followed by the addition of 0.5 mL protein solution or TN buffer for TBAP-α under N_2 gas flow. Samples were irradiated using a solar simulator with an output of 1.0 sun for 2 h (AM1.5G, Class AAA, IEC/JIS/ASTM, 1440 W Xenon, 12 × 12 in., MODEL: 94123A). Error bars represent the standard deviation of the mean of three independent experiments.

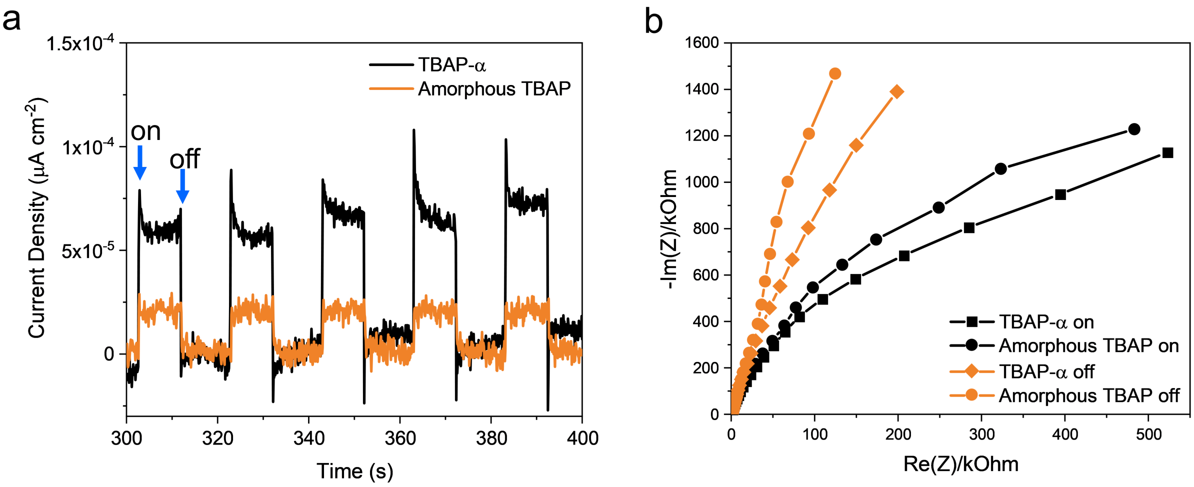

Figure S20. (a) Photocurrent response, (b) EIS analysis for amorphous TBAP and TBAP-α with applied potentials at 0.4 V vs. Ag/AgCl under intermittent irradiation (300 W Xe light source with λ > 420 nm filter). TBAP-α generated a 50% higher photocurrent density compared to amorphous TBAP, indicating better electron-hole separation performance. The arc radius of EIS plots varied in the order TBAP-α < amorphous TBAP, suggesting that TBAP-α exhibited lower electrical resistance than the amorphous TBAP.

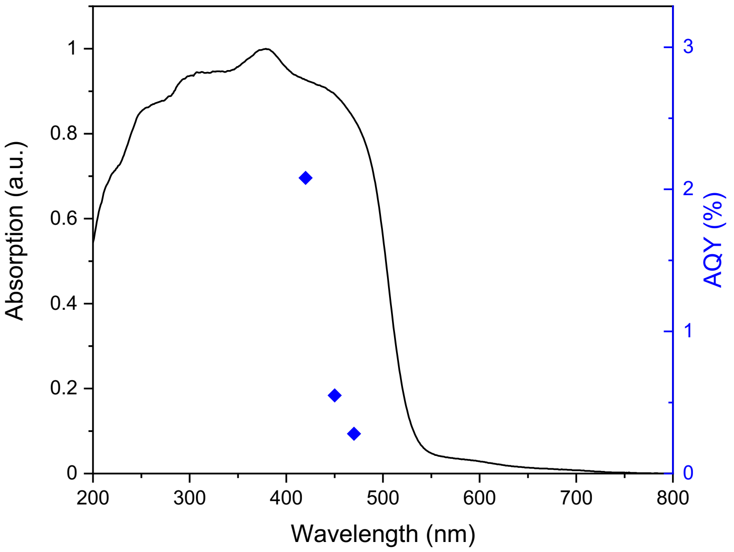

Figure S21. Wavelength-dependent AQY values (measured in the first 1.5 hours) and solid-state UV-visible spectrum of H-S|TBAP-α.

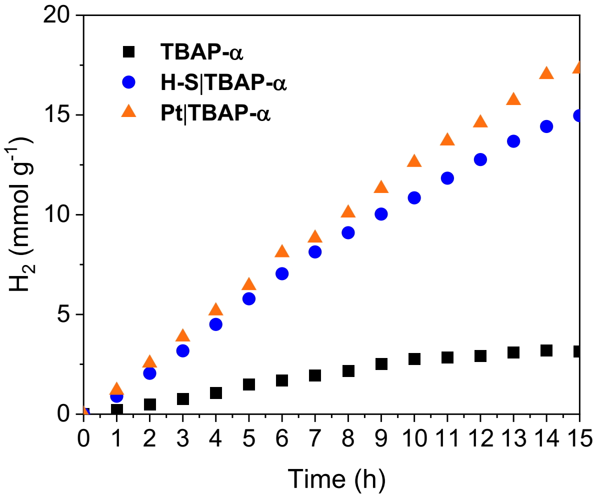

Figure S22. H_2 evolution of TBAP-α, H-S|TBAP-α and 1 wt.% Pt|TBAP-α as a function of time (300 W Xe light source with λ > 420 nm filter).

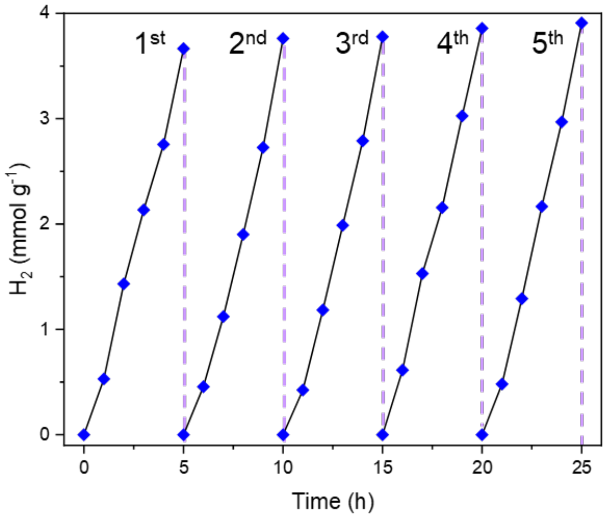

Figure S23. Cycling measurements for the photocatalytic hydrogen evolution of H-S|TBAP-α (300 W Xe light source with λ > 420 nm filter).

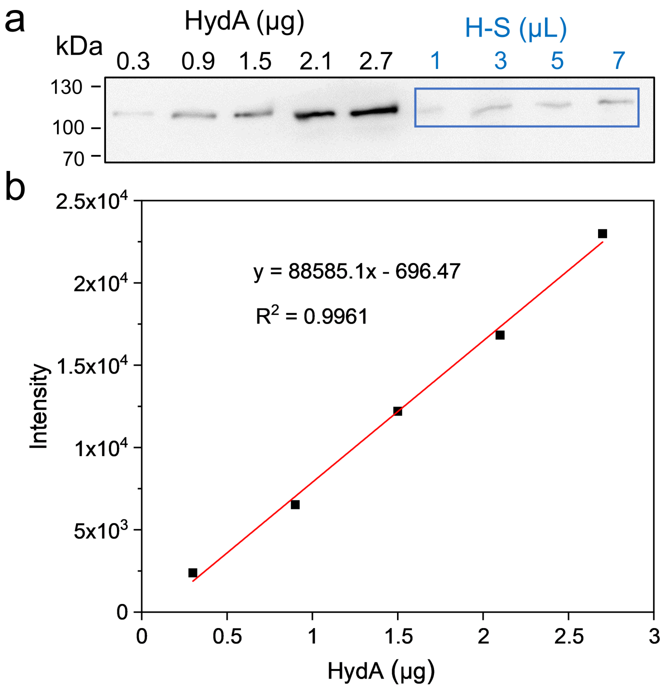

Figure S24. (a) Immunoblot analysis of purified HydA (Fd-HydA-EP) using 6×His antibody. HydA was purified by immobilized-nickel affinity chromatography followed by quantification by BCA protein assay kit. 0.3 μg, 0.9 μg, 1.5 μg, 2.1 μg and 2.7 μg of HydA and 1, 3, 5, 7 μL H-S were loaded onto each well of the gel, respectively. (b) Linear relationship between HydA content quantified by BCA protein assay kit and the corresponding HydA content quantification by immunoblot analysis. Densitometric quantitation of HydA levels was determined using ImageJ.

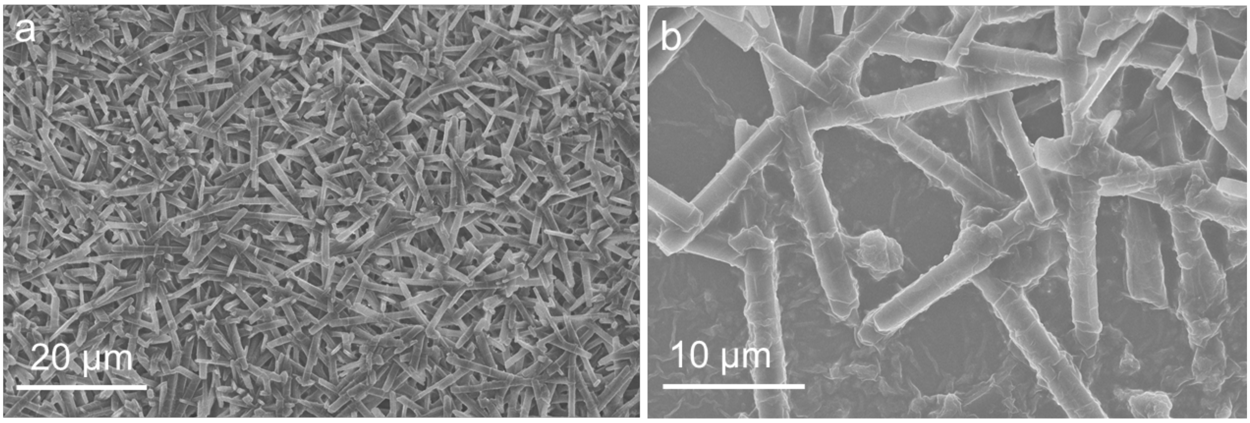

Figure S25. SEM images of H-S|TBAP-α after 30 hours irradiation (300 W Xe lamp, λ > 420 nm) (a) in a larger scale, (b) in a smaller scale.

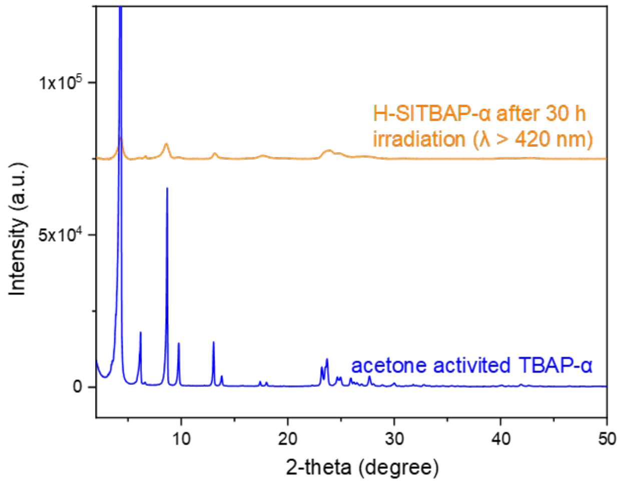

Figure S26. **PXRD patterns of acetone activated TBAP-α (blue line) and H-S|TBAP-α after 30 hours irradiation (300 W Xe lamp, λ > 420 nm)) (orange line) and acetone-activated TBAP-α without irradiation (blue line).**

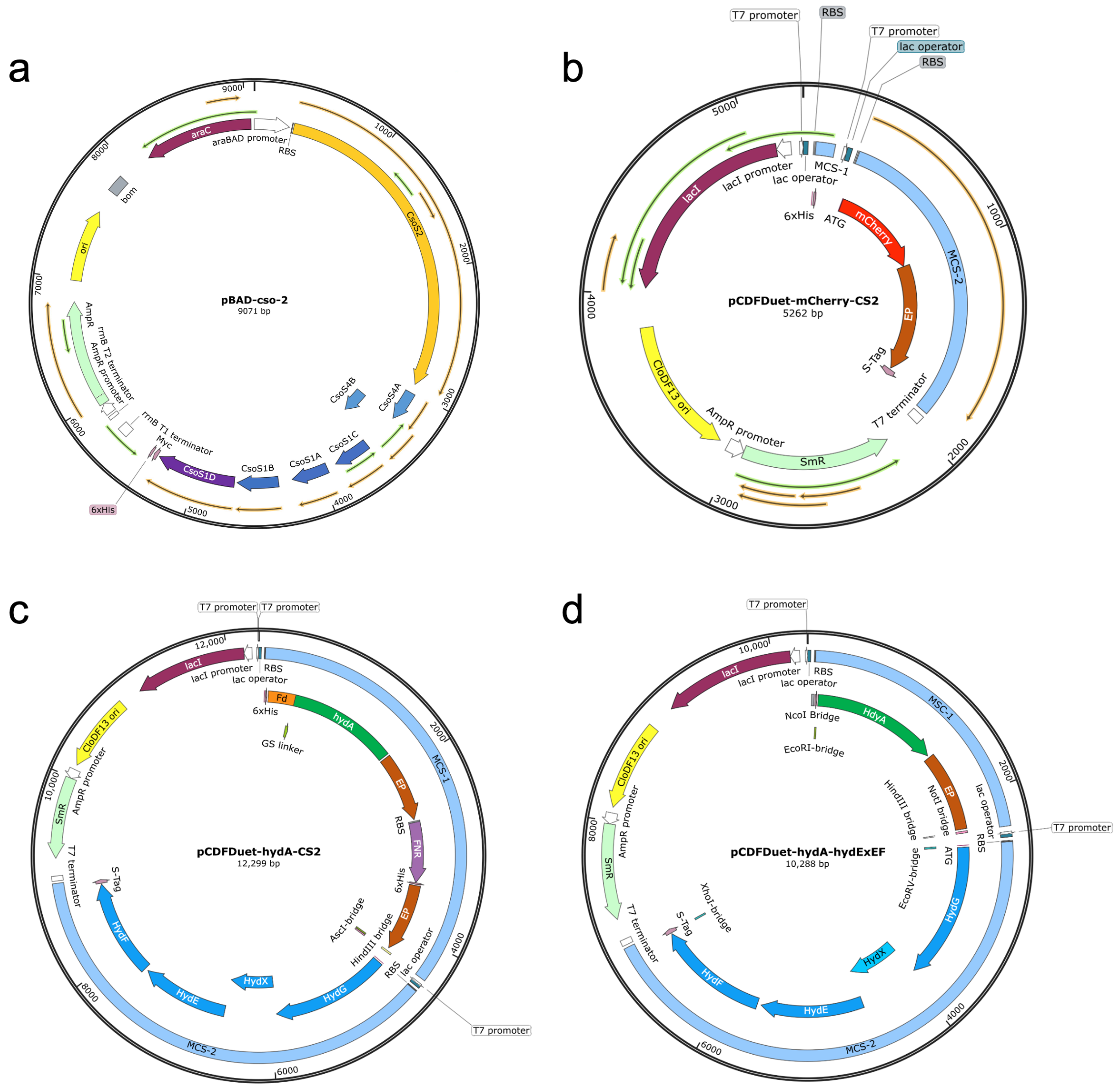

Figure S27. Plasmid sequence maps in this work. (a) *pBAD-cso-2* (shell); (b) *pCDFDuet-mCherry-CS2* (mCherry-EP); (c) *pCDFDuet-hydA-CS2* (hydA-EP) and (d) *pCDFDuet-hydA-hydGxEF-CS2* (HydA-HydGxEF-EP). Images are generated by SnapGene@ Viewer 5.3.2. The corresponding expressed protein names are in brackets.

SUPPLEMENTARY TABLES

Table S1. TBAP-α crystal data and structure refinement.

	**FRT**
Empirical formula	$C_{44}H_{26}O_8$
Formula weight	682.65
Temperature/K	300.00(10)
Crystal system	monoclinic
Space group	*C2/m*
a/Å	29.1153(15)
b/Å	28.3944(17)
c/Å	3.9048(4)
α/°	90
β/°	92.242(6)
γ/°	90
Volume/Å^3	3225.7(4)
Z	2
ρcal/(g cm^{-3})	0.703
μ/mm^{-1}	0.397
F(000)	708.0
Crystal size/mm^3	0.3 × 0.1 × 0.1
Radiation	Cu Kα (λ = 1.54184)
2Θ range for data collection/°	6.076 to 140.138
Index ranges	$-35 \leq h \leq 35$, $-34 \leq k \leq 30$, $-4 \leq l \leq 4$
Reflections collected	11334
Independent reflections	3105 [R_{int} = 0.1000, R_{sigma} = 0.0807]
Data/restraints/parameters	3105/0/123
Goodness-of-fit on F^2	1.027
Final R indexes [I>=2σ (I)]	R_1 = 0.0734, wR_2 = 0.2206
Final R indexes [all data]	R_1 = 0.0871, wR_2 = 0.2341
Largest diff. peak/hole / e Å^{-3}	0.37/-0.24

Table S2. Protein components in recombinant α-carboxysomes from *E. coli*[5].

Protein	Structure of functional unit	Molecular Weight (kDa)	Functional units of multimer per carboxysome	Percentage of functional units of multimer per carboxysome (%)
CsoS1AC	Hexamer	10.0	1001 ± 45	60.74
CsoS1B	Hexamer	11.3	79 ± 9	4.79
CsoS1D	Pseudohexamer	23.5	1 ± 0	0.06
CsoS4A	Pentamer	8.9	6 ± 1	0.36
CsoS4B	Pentamer	8.8	1 ± 1	0.06
CsoS2A	Monomer	92.4	305 ± 9	18.51
CsoS2B	Monomer	124.2	249 ± 13	15.11

Table S3. Non-covalent interactions between TBAP-α and amino acid residues of CsoS1A calculated by MD simulations. Only the interactions with over 80% probability were listed, which indicate the reliable interactions and binding sites.

Interaction	TBAP molecule number/atom	Amino acid number/name/atom	Average length (Å)	Average angles (°)	Total interaction probability of amino acids with TBAP-α
H-bonding	102/O2	243/Pro/O.CO2	1.9059	160.0159	1
H-bonding	80/O2	521/Glu/O.CO2	2.0172	157.0975	1
H-bonding	81/O2	518/Ser/O.CO2	2.1871	139.8752	0.998
H-bonding	101/O2	241/Lys/O.CO2	1.8916	153.2898	0.996
H-bonding	70/O2	761/Lys/O.CO2	1.8967	154.0540	0.994
H-bonding	106/O2	623/Pro/O.CO2	1.7756	158.8326	0.98
H-bonding	81/O.CO2	522/Asn/Nam	2.7116	123.3724	0.92
H-bonding	80/O.CO2	522/Asn/Nam	1.9748	148.9200	0.906
Salt bridge	TBAP_45	B-LYS	/	/	0.966
Salt bridge	TBAP_48	D_ARG	/	/	0.952
Salt bridge	TBAP_49	D_ARG	/	/	0.894
Salt bridge	TBAP_42	B_ARG	/	/	0.858
Salt bridge	TBAP_80	D_LYS	/	/	0.852

Table S4. The statistics of residues from TBAP-α and CsoS1A protein with decomposition binding energy over 1 kcal mol^{-1} and the corresponding energy differences determined by MD simulations.

Amino acid number OR TBAP molecule number	ΔGvdW	ΔGelec	ΔGsolv	ΔGbind
A_PRO243	-1.68	-4.83	1.53	-4.98
D_GLU521	-1.50	-2.44	-0.52	-4.46
D_SER518	-0.96	-3.51	1.94	-2.52
E_PRO623	-0.70	-3.56	1.77	-2.49
F_ALA717	-1.19	-0.91	0.24	-1.86
D_ASN522	-1.60	-3.26	3.32	-1.54
F_GLN719	-1.10	-1.47	1.11	-1.45
D_ARG466	-1.04	-10.19	10.14	-1.09
C_GLY343	-0.73	-0.77	0.40	-1.09
F_LYS716	-0.96	-0.76	0.68	-1.03
C_THR342	-1.41	-1.53	1.93	-1.01
TBAP80	-2.07	-6.38	4.14	-4.32
TBAP106	-1.51	-4.51	1.97	-4.05
TBAP102	-2.34	-2.99	1.84	-3.49
TBAP70	-1.49	-5.25	3.61	-3.14
TBAP48	-0.56	-6.32	4.47	-2.41
TBAP101	-1.69	-3.05	2.78	-1.96
TBAP103	-2.02	-1.94	2.09	-1.87
TBAP112	-1.34	-2.14	1.66	-1.83
TBAP81	-2.45	-2.57	3.35	-1.67
TBAP45	-0.61	-4.36	3.68	-1.28

GvdW is the van der Waals energy and Gelec is the electrostatic energy. All energy differences correspond to the energy of the respective complex subtracted by the energies of the ligand and receptor. ΔGbind = ΔGvdW + ΔGelec + ΔGsolv.

Table S5. Estimated fluorescence lifetimes of TBAP-α and H-S|TBAP-α.

Samples	τ_1 (ns)	τ_2 (ns)	τ_3 (ns)	A_1	A_2	A_3	τ_A (ns)*
TBAP-α	0.513	1.989	15.532	4.374	91.091	1.535	3.51
H-S|TBAP-α	0.789	2.007	10256	13.354	80.679	5.968	4.11

* τ_A is the average PL lifetime calculated from the following equation[6]:

$$\tau_A = \sum_{i=1}^{3}(A_i\tau_i^2)/\sum_{i=1}^{3}(A_i\tau_i)$$

Table S6. Hydrogen evolution reaction (HER) conditions in this work.

Sample Name	Materials	Sacrificial condition[a]	Protein[b]	$Pt^{[c]}$ (wt%)	HER[d] (µmol (g TBAP-α)$^{-1}$ h^{-1})
H-S|TBAP-α	TBAP-α	0.1 M AA, neutralised to pH 7 with NaOH	H-S	/	3976.4
			/		707.4
			H-S (5%)		2204.4
			H-S (50%)		2489.0
H-S|TBAP-α dark[e]			H-S		< 0.1[f]
$H\text{-}S_{ox}$|TBAP-α			$H\text{-}S_{ox}$		760.6
H-S|TBAP-α in H_2O		H_2O			< 0.1
H-S	/	0.1 M AA, neutralised to pH 7 with NaOH	H-S		< 0.1
$H\text{-}S_{ox}$	$Na_2S_2O_4$-MV	/	$H\text{-}S_{ox}$		< 0.1
TBAP-Me Crystal	TBAP-Me	0.1 M AA, neutralised to pH 7 with NaOH	H-S		6.96
Pyrene Crystal	Pyrene				< 0.1
Amorphous TBAP	As-synthesized amorphous TBAP				787.5
EY with H-S	Eosin Y	0.15 M TEOA, adjusted to pH 7.0 with HCl			107.2
$EY\text{-}TiO_2$ with H-S	$EY\text{-}TiO_2$				9.7
FS-COF with H-S	FS-COF	0.1 M AA, pH 2.6	H-S		< 0.1
FS-COF with Pt			/	8	5988.4
FS-COF with H-S		0.1 M AA, neutralised to pH 7 with NaOH	H-S	/	< 0.1
FS-COF with Pt			/	8	207.9

[a] 0.1 M AA aqueous solutions were adjusted to pH 7 using 1 M NaOH $_{(aq)}$.

[b] H-S was purified by sucrose gradient and diluted with TN buffer to the total protein concentration of 1 mg mL^{-1} measured by Nanodrop. 0.5 mL (the final content of total protein to synthetic materials is 25 %) protein was added unless otherwise specified.

[c] Pt loaded by photodeposition of H_2PtCl_6 (aqueous solution, 8 wt.%).

[d] Photocatalytic reaction condition: Unless otherwise stated, photocatalysts (2 mg) except for Eosin Y (EY, 1.4 mg) and $EY\text{-}TiO_2$ (5 mg) were suspended in 4.5 mL either aqueous ascorbic acid (AA, 0.1 M, pH 7.0) or aqueous triethanolamine (TEOA, 0.15 M, pH 7.0) with the addition of 0.5 mL H-S in TN buffer or TN buffer as control. All the samples were irradiated by the solar simulator (AM1.5G, Class AAA, IEC/JIS/ASTM, 1440 W Xenon, 12 × 12 in., MODEL: 94123A) for 2 h.

[e] The sample was measured without irradiation.

[f] The hydrogen produced was below the detection limit of Shimadzu GC-2010.

Table S7. Lists of hydrogen production performance of the state-of-the-art photocatalyst-hydrogenase hybrid systems. We note here that hydrogen production rates are unique to the precise photocatalytic set-up (that is, not only the light intensity and wavelength) and hence comparisons of absolute hydrogen evolution rates (HERs) between studies should be made cautiously.

Photosen-sitizer	Hydrogenase	Sacrificial agent	Light source & Wavelength	Redox Mediator	HER	t_{max}	Ref
TBAP-α	[FeFe]-H₂ease[a] Encased with α-carboxysome (H-S)	0.1 M Ascorbic Acid, neutralized to pH 7 with NaOH	300 W Xe lamp (> 420 nm)	No	1,137.8 ± 47.2 μmol H_2 (g TBAP-α)$^{-1}$ h^{-1} OR 11,694 ± 484.5 mol H_2 (mol H_2ease)$^{-1}$ h^{-1} OR 106.3 ± 4.4 μmol H_2 (mg H_2ease)$^{-1}$ h^{-1} in 30 h	30 h+	This work
MPA capped-CdS nanorod	[FeFe]-H_2ase I[d]	0.1 M Ascorbic Acid	LED (405 nm, I = 800 μE m^{-2} s^{-1})	No	3 mol H_2 (mol CaI)$^{-1}$ s^{-1}	6 h	7
			White light (I = 30,000 μE m^{-2} s^{-1})	No	983 mol H_2 (mol CaI)$^{-1}$ s^{-1}	/	
Ru(II)-photosensitizer	[NiFe]-H_2ase[b]	0.045 M EDTA, adjusted to pH 5.5	150,000 lx Xe lamp	4 mM MV	224 ± 3 nmol H_2 (mg protein)$^{-1}$ min^{-1}	/	8
g-C_3N_4	[NiFeSe]-H_2ease[c]	0.1 M EDTA, adjust to pH 6	W-halogen lamp (1000 W, 100 mW cm^{-2}) (> 300 nm)	No	5,532 ± 553 mol H_2 (mol H_2ase)$^{-1}$ h^{-1} or 55.3 ± 5.5 μmol H_2 (g CNx)$^{-1}$ h^{-1} after 1 h	48 h+	9
				5 μmol MV	22 x improvement		
CNx-TiO_2 hybrid	[NiFeSe]-H_2ease[c]	0.1 M EDTA, adjust to pH 6	Xe lamp (1000 W, 100 mW cm^{-2}) (> 420 nm)	No	63 ± 6 μmol H_2 (g CNx-TiO_2)$^{-1}$ h^{-1} after 1 h	72 h+	10
				5 μmol MV	183 μmol H_2 (g CNx-TiO_2)$^{-1}$ h^{-1} after 72 h		
Eosin Y	[NiFeSe]-H_2ease[c]	0.15 M TEOA, neutralised to pH 7 with HCl	solar light simulator (AM 1.5 G, 100 mW cm^{-2}) (>420 nm)	No	13.9 ± 0.7 mol H_2 (mol H_2ease)$^{-1}$ s^{-1} in 15 h	24 h	11
RuP-TiO_2	[NiFeSe]-H_2ease[c]	0.025 M TEOA, neutralised to pH 7 with HCl	W-halogen lamp (250 W, 45 mW cm^{-2})	NO	712 μmol H_2 (g TiO_2)$^{-1}$ h^{-1} OR 50 mol H_2 (mol H_2ease)$^{-1}$ s^{-1} after 1 h	72 h	12
	[FeFe]-H_2ease[a]				168 μmol H_2 (g TiO_2)$^{-1}$ h^{-1} after 1 h		
nc-CdTe	[FeFe]-H_2ase I[d]	0.05 M Ascorbic Acid, adjust to pH 4.75	USHIO halogen projector lamp (150 W, 21 V)	No	1.94 ± 0.30 μmol H_2 (mg H_2ease)$^{-1}$ min^{-1} after 5 min	50 min (max shown)	13
CD-$NHMe_2^+$	[NiFeSe]-H_2ase[c]	0.1 M EDTA, adjust to pH 6.0	Xe lamp (AM 1.5 G, 100 mW cm^{-1})	No	3.9 ± 0.9 ×10^3 mol H_2 (mol H_2ease)$^{-1}$ h^{-1}	48 h	14
				5 μM MV	6 x improvement	72 h	
aspartic acid-based carbon dots (AspCDs)	[FeFe]-H_2ease (CrHydA1) [a]	0.67 M TEOA, neutralised to pH 7 with HCl	LED (I = 50 mW cm^{-1}) (420 - 700 nm)	No	1.73 μmol H_2 (mg H_2ease)$^{-1}$ min^{-1}	24 h	15
				10 μmol MV		170 h	

[a] [FeFe]-H_2ase from Chlamydomonas reinhardtii (Cr);

[b] [NiFe]-H_2ase from T. roseopersicina;

[c] [NiFeSe]-H_2ase from Desulfomicrobium baculatum (Dmb);

[d] [FeFe]-H_2ase I from C. acetobutylicum.

Table S8. Primers used for pCDFDuet-mCherry-CS2 plasmid construction.

Primers	Primer sequence
M-F	AGTATAAGAAGGAGATATACATATGGTGAGCAAGGGCGAGGAGGATAAC
M-C-R	AGCTTCGGGCTCTGGGGTGCTCGTCTTGTACAGCTCGTCCAT
M-C-F	ATGGACGAGCTGTACAAGACGAGCACCCCAGAGCCCGAAGCT
C-R	CAGCGGTTTCTTTACCAGACTCGATCAACCGCGCGCGCCGCCGG

Table S9. Plamisd gene sequences in this work.

Plasmid	Gene Sequence
pBAD-cso-2	AAGAAACCAATTGTCCATATTGCATCAGACATTGCCGTCACTGCGTCTTTTACTGGCTC<br>TTCTCGCTAACCAAACCGGTAACCCCGCTTATTAAAAGCATTCTGTAACAAAGCGGGA<br>CCAAAGCCATGACAAAAACGCGTAACAAAAGTGTCTATAATCACGGCAGAAAAGTCC<br>ACATTGATTATTTGCACGGCGTCACACTTTGCTATGCCATAGCATTTTTATCCATAAGA<br>TTAGCGGATCCTACCTGACGCTTTTTATCGCAACTCTCTACTGTTTCTCCATACCCGTTT<br>TTTGGGCTAACAGGAGGAATTAACCATGGGGTCAAACATGCCTTCACAGTCAGGAATG<br>AATCCTGCCGACCTGAGCGGACTCTCTGGCAAGGAACTGGCACGCGCACGCCGCGCTG<br>CACTATCCAAGCAAGGGAAAGCAGCTGTTTCTAATAAAACGGCTAGCGTAAACCGTAG<br>CACTAAACAGGCGGCATCTTCGATCAATACAAATCAGGTGCGCTCTTCTGTAAATGAA<br>GTGCCCACTGATTACCAAATGGCGGATCAATTGTGCTCTACGATTGATCATGCTGACTT<br>TGGTACCGAAAGCAATCGCGTTAGAGATCTCTGCCGTCAACGCAGAGAGGCACTATCA<br>ACTATCGGTAAAAAAGCGGTTAAAACCAACGGCAAGCCGTCGGGTCGCGTTCGACCA<br>CAGCAATCAGTGGTTCACAACGACGCAATGATCGAAAATGCCGGTGATACTAACCAAT<br>CATCGTCCACTTCATTAAATAATGAACTTTCCGAAATCTGCTCCATAGCAGACGACATG<br>CCGGAGCGTTTTGGTTCACAAGCCAAAACCGTCCGTGATATCTGCCGTGCACGCCGTC<br>AAGCGCTCTCTGAGCGTGGAACTCGCGCCGTGCCGCCAAAGCCGCAATCTCAAGGTGG<br>TCCAGGACGCAATGGCTATCAAATTGATGGATACCTAGATACCGCACTTCATGGCCGC<br>GATGCCGCCAAGCGCCACCGTGAAATGCTCTGTCAATACGGCCGCGGCACAGCACCTT<br>CCTGCAAGCCAACAGGCCGTGTCAAAAATTCTGTACAGTCGGGCAACGCAGCGCCAA<br>AAAAGGTTGAAACCGGTCACACCCTATCCGGCGGATCTGTTACGGGCACGCAAGTGGA<br>TCGTAAATCTCATGTGACTGGAAACGAGCCGGGCACTTGCCGAGCAGTCACGGGCACC<br>GAGTACGTAGGTACTGAGCAATTCACCTCTTTTTGCAATACCAGCCCCAAGCCAAATG<br>CGACGAAGGTCAATGTGACCACAACGGCTCGTGGTCGCCCTGTTAGCGGTACGGAAGT<br>TTCACGGACCGAGAAGGTAACTGGCAACGAATCCGGTGTCTGCCGTAACGTTACCGGC<br>ACCGAATACATGAGTAATGAAGCTCACTTTTCTCTATGTGGAACAGCCGCAAAGCCTT<br>CACAAGCGGATAAAGTCATGTTCGGCGCCACAGCACGAACGCATCAAGTGGTCAGTG<br>GCAGTGATGAATTCAGGCCCTCTTCTGTTACGGGTAACGAATCGGGTGCAAAACGCAC<br>AATTACCGGCTCGCAGTACGCAGACGAAGGTCTTGCGCGACTCACGATCAACGGAGCA<br>CCTGCAAAAGTAGCCAGAACCCACACCTTTGCGGGCTCTGACGTTACCGGCACGGAAA<br>TCGGTCGCTCTACTCGCGTAACTGGTGATGAAAGCGGTTCGTGTCGTTCAATCTCAGGC

	ACCGAGTATCTCAGTAACGAGCAATTCCAATCCTTTTGTGACACAAAACCTCAACGCA<br>GCCCGTTCAAGGTTGGCCAAGATCGCACGAACAAGGGTCAGTCTGTGACTGGTAACTT<br>GGTTGATCGTTCCGAACTGGTTACAGGTAACGAACCAGGTTCATGCTCTCGGGTTACA<br>GGCTCTCAGTATGGCCAAAGCAAAATCTGCGGTGGTGGCGTGGGAAAAGTGCGCTCA<br>ATGCGCACCCTTCGCGGCACCTCAGTATCTGGCCAACAGCTAGATCATGCCCCAAAGA<br>TGTCCGGTGACGAGCGCGGCGGGTGCATGCCCGTCACCGGTAATGAGTACTACGGTCG<br>TGAACATTTCGAACCGTTTTGTACGAGCACCCCAGAGCCCGAAGCTCAATCAACTGAA<br>CAATCATTGACCTGTGAAGGACAAATTATTAGCGGCACTTCAGTTGACGCCAGTGATT<br>TGGTCACAGGAAATGAAATCGGTGAACAGCAACTCATCAGCGGTGACGCCTATGTTGG<br>CGCGCAGCAGACAGGTTGCCTTCCCACTAGTCCACGCTTCAACCAAACTGGCAATGTT<br>CAGTCAATGGGTTTTAAGAACACCAATCAGCCAGAACAAAACTTTGCACCAGGTGAAG<br>TAATGCCTACTGACTTTAGTATTCAAACCCCAGCTCGCTCGGCTCAGAATCGCATTACA<br>GGTAACGACATTGCGCCCTCAGGTCGCATTACAGGCCCTGGTATGCTGGCAACCGGCT<br>TGATTACAGGAACCCCCGAATTCAGGCACGCTGCGCGCGAGTTGGTTGGTTCTCCACA<br>ACCCATGGCAATGGCCATGGCCAACCGTAATAAGGCTGCTCAAGCACCTGTTGTGCAG<br>CCAGAAGTGGTTGCAACTCAGGAAAAGCCTGAGTTGGTATGTGCACCAAGAAGCGAT<br>CAAATGGATCGTGTGAGTGGCGAAGGCAAAGAACGTTGCCACATCACTGGCGATGAC<br>TGGTCAGTTAACAAGCACATCACCGGTACAGCCGGTCAATGGGCGAGTGGTCGCAACC<br>CTTCCATGCGCGGTAATGCGCGTGTGGTCGAAACCAGCGCGTTTGCCAATCGCAATGT<br>GCCAAAACCTGAAAAGCCGGGCTCCAAGATCACGGGCAGTAGTGGTAATGACACCCA<br>AGGTAGTCTGATCACTTACTCCGGCGGCGCGCGCGGTTGATTAAGTAAAGTGTAACGA<br>TCTTTGAGCGTTCAGGCGCAGGATATCGGAAGCCCGATTGAAGAGGTTGCATCCGCAT<br>GAAAATCATGCAAGTTGAGAAAACGTTGGTTTCAACAAACCGTATTGCTGATATGGGT<br>CACAAACCACTATTAGTGGTATGGGAGAAACCGGGCGCGCCCAGGCAGGTCGCCGTG<br>GATGCGATTGGCTGCATACCGGGCGATTGGGTTTTGTGCGTTGGGTCATCGGCAGCAC<br>GAGAGGCTGCAGGAAGCAAGTCTTATCCCTCTGATTTGACGATTATCGGGATTATTGA<br>TCAGTGGAATGGTGAGTAATGGAAGTAATGCGCGTTCGTTCCGACCTAATCGCAACAC<br>GCAGGATTCCCGGTCTTAAAAATATCTCTTTGCGTGTCATGGAGGATGCTACGGGTAA<br>GGTCAGTGTCGCTTGCGATCCCATTGGCGTTCCCGAGGGATGTTGGGTCTTTACGATTA<br>GCGGCTCTGCCGCTCGGTTTGGCGTGGGTGATTTTGAGATTCTCACGGATTTGACGATT<br>GGTGGCATCATCGATCACTGGGTAACTTGACCATCGCTAGATGAGTTGATTTTGAATG<br>AGTCTTTATTGAGGAGAGAAGAAATGGCAGCAGTAACAGGTATTGCACTGGGTATGAT<br>TGAAACACGTGGTCTGGTTCCAGCGATTGAAGCTGCCGATGCCATGACCAAGGCCGCC<br>GAAGTACGTTTGGTTGGCCGTCAATTTGTTGGTGGTGGTTACGTGACCGTTTTGGTCCG<br>TGGTGAAACCGGTGCCGTCAACGCAGCAGTTCGTGCGGGCGCTGATGCCTGCGAACGA<br>GTCGGCGATGGTCTGGTCGCGGCGCATATCATTGCCCGTGTCCATTCCGAAGTCGAAA<br>ACATCCTGCCGAAAGCCCCTGAAGCTTAAGGATTGGGAAAGACGAACCGGCGCAGGC<br>TTGTTCCGGTTCTTTGCATAAAGTGACAGCTTAGGAGTTTATTTAAATGGCTGATGTAA<br>CTGGTATTGCTCTGGGTATGATCGAAACACGTGGCTTAGTTCCTGCGATTGAAGCAGC<br>GGACGCCATGACTAAAGCGGCTGAAGTGCGTTTGGTCGGTCGTCAATTTGTTGGTGGC<br>GGTTACGTCACCGTATTGGTTCGGGGCGAAACAGGCGCTGTTAACGCCGCTGTTCGTG

	CTGGCGCCGATGCTTGCGAACGTGTTGGTGATGGCTTGGTTGCTGCGCACATCATTGCG<br>CGTGTCCACTCAGAAGTAGAAAACATTCTGCCTAAGGCGCCACAAGCCTAAGTCAGAT<br>ATTCCTAAGACGGCTCACTTCGGCGACACCCGTCGCCGAAACCGTCTAACCAAATTTC<br>TGGGCTCGTTCTGAACGTGCTCAACACTAGTTTTAGAGGATCTGTTATGGCAACGACTC<br>ACGGTATTGCCCTGGGCATGATTGAAACACGAGGATTGGTTCCTGCCATTGAAGCCGC<br>AGATGCCATGACCAAAGCGGCGGAAGTCCGTCTGGTCGGACGATCATTTGTTGGCGGC<br>GGTTACGTGACCGTAATGGTTCGTGGTGAGACAGGTGCAGTAAATGCTGCCGTTCGTG<br>CGGGTGCTGACGCCTGTGAACGTGTTGGCGATGGCCTGGTTGCTGCGCACATCATTGC<br>GCGCGTTCATTCTGAAGTTGAGATCATCCTACCCGAGACGCCCGAAGACTCAGATTCC<br>GCGTGGTGTATCGCAAATCTGAATAGCTAATGTCTAGTAGGGAAGATGCGCATGAACA<br>ACATTGATTTGCGCGTCTATTCGTTTATCGATTCGCTTCAACCGCAGCTTGCATCCTATC<br>TTGCGACATCATCGCAAGGCTTTCTTCCCGTTCCGGGTGATGCCTGCTTGTGGATTGAA<br>GTCGCGCCGGGCATGGCCGTCCATCGCCTCAGTGATATTGCGCTAAAGGCCACGAACG<br>TTCGTCTCGGCGAACAGGTAGTCGAGCGTGCTTTTGGCTCGATGGAAATTCATTACCG<br>AAACCAAAGCGACGTTCTCGCATCCGGTGAGGCCGTTTTAAGAGAAATCAATCACGCG<br>CAAGAAGATCGTCTGCCTTGTCGCATCGCATGGAAAGAGATCATTCGAGCGATTACCC<br>CCGATCATGCCACCTTGATCAATCGCCAGTTGCGTAAAGGCTCTATGCTGTTGCCGGGC<br>AAAAGCATGTTCATCCTTGAAACAGAACCGGCAGGTTATATTGTTCAGGCTGCCAACG<br>AGGCCGAGAAAGCAGCTCATGTGACTCTGATCGATGTACGTGCTTTTGGTAACTTTGG<br>TCGCCTGACCATGATGGGCAGCGAAGCGGAAACCGAAGAAGCCATGCGGGCGGCTGA<br>GGCCACAATCGCAAGCATCAACGCGCGTGCGCGTCGCGCTGAAGGGTTCTAATAACGA<br>AGCTTACGTAGAACAAAAACTCATCTCAGAAGAGGATCTGAATAGCGCCGTCGACCAT<br>CATCATCATCATCATTGAGTTTAAACGGTCTCCAGCTTGGCTGTTTTGGCGGATGAGAG<br>AAGATTTTCAGCCTGATACAGATTAAATCAGAACGCAGAAGCGGTCTGATAAAACAG<br>AATTTGCCTGGCGGCAGTAGCGCGGTGGTCCCACCTGACCCCATGCCGAACTCAGAAG<br>TGAAACGCCGTAGCGCCGATGGTAGTGTGGGGTCTCCCCATGCGAGAGTAGGGAACTG<br>CCAGGCATCAAATAAAACGAAAGGCTCAGTCGAAAGACTGGGCCTTTCGTTTTATCTG<br>TTGTTTGTCGGTGAACGCTCTCCTGAGTAGGACAAATCCGCCGGGAGCGGATTTGAAC<br>GTTGCGAAGCAACGGCCCGGAGGGTGGCGGGCAGGACGCCCGCCATAAACTGCCAGG<br>CATCAAATTAAGCAGAAGGCCATCCTGACGGATGGCCTTTTTGCGTTTCTACAAACTCT<br>TTTGTTTATTTTTCTAAATACATTCAAATATGTATCCGCTCATGAGACAATAACCCTGA<br>TAAATGCTTCAATAATATTGAAAAAGGAAGAGTATGAGTATTCAACATTTCCGTGTCG<br>CCCTTATTCCCTTTTTTGCGGCATTTTGCCTTCCTGTTTTTGCTCACCCAGAAACGCTGG<br>TGAAAGTAAAAGATGCTGAAGATCAGTTGGGTGCACGAGTGGGTTACATCGAACTGG<br>ATCTCAACAGCGGTAAGATCCTTGAGAGTTTTCGCCCCGAAGAACGTTTTCCAATGAT<br>GAGCACTTTTAAAGTTCTGCTATGTGGCGCGGTATTATCCCGTGTTGACGCCGGGCAA<br>GAGCAACTCGGTCGCCGCATACACTATTCTCAGAATGACTTGGTTGAGTACTCACCAG<br>TCACAGAAAAGCATCTTACGGATGGCATGACAGTAAGAGAATTATGCAGTGCTGCCAT<br>AACCATGAGTGATAACACTGCGGCCAACTTACTTCTGACAACGATCGGAGGACCGAAG<br>GAGCTAACCGCTTTTTTGCACAACATGGGGGATCATGTAACTCGCCTTGATCGTTGGG<br>AACCGGAGCTGAATGAAGCCATACCAAACGACGAGCGTGACACCACGATGCCTGTAG

	CAATGGCAACAACGTTGCGCAAACTATTAACTGGCGAACTACTTACTCTAGCTTCCCG<br>GCAACAATTAATAGACTGGATGGAGGCGGATAAAGTTGCAGGACCACTTCTGCGCTCG<br>GCCCTTCCGGCTGGCTGGTTTATTGCTGATAAATCTGGAGCCGGTGAGCGTGGGTCTCG<br>CGGTATCATTGCAGCACTGGGGCCAGATGGTAAGCCCTCCCGTATCGTAGTTATCTAC<br>ACGACGGGGAGTCAGGCAACTATGGATGAACGAAATAGACAGATCGCTGAGATAGGT<br>GCCTCACTGATTAAGCATTGGTAACTGTCAGACCAAGTTTACTCATATATACTTTAGAT<br>TGATTTAAAACTTCATTTTTAATTTAAAAGGATCTAGGTGAAGATCCTTTTTGATAATC<br>TCATGACCAAAATCCCTTAACGTGAGTTTTCGTTCCACTGAGCGTCAGACCCCGTAGA<br>AAAGATCAAAGGATCTTCTTGAGATCCTTTTTTTCTGCGCGTAATCTGCTGCTTGCAAA<br>CAAAAAAACCACCGCTACCAGCGGTGGTTTGTTTGCCGGATCAAGAGCTACCAACTCT<br>TTTTCCGAAGGTAACTGGCTTCAGCAGAGCGCAGATACCAAATACTGTCCTTCTAGTGT<br>AGCCGTAGTTAGGCCACCACTTCAAGAACTCTGTAGCACCGCCTACATACCTCGCTCT<br>GCTAATCCTGTTACCAGTGGCTGCTGCCAGTGGCGATAAGTCGTGTCTTACCGGGTTGG<br>ACTCAAGACGATAGTTACCGGATAAGGCGCAGCGGTCGGGCTGAACGGGGGGTTCGT<br>GCACACAGCCCAGCTTGGAGCGAACGACCTACACCGAACTGAGATACCTACAGCGTG<br>AGCTATGAGAAAGCGCCACGCTTCCCGAAGGGAGAAAGGCGGACAGGTATCCGGTAA<br>GCGGCAGGGTCGGAACAGGAGAGCGCACGAGGGAGCTTCCAGGGGGAAACGCCTGGT<br>ATCTTTATAGTCCTGTCGGGTTTCGCCACCTCTGACTTGAGCGTCGATTTTTGTGATGCT<br>CGTCAGGGGGGCGGAGCCTATGGAAAAACGCCAGCAACGCGGCCTTTTTACGGTTCCT<br>GGCCTTTTGCTGGCCTTTTGCTCACATGTTCTTTCCTGCGTTATCCCCTGATTCTGTGGA<br>TAACCGTATTACCGCCTTTGAGTGAGCTGATACCGCTCGCCGCAGCCGAACGACCGAG<br>CGCAGCGAGTCAGTGAGCGAGGAAGCGGAAGAGCGCCTGATGCGGTATTTTCTCCTTA<br>CGCATCTGTGCGGTATTTCACACCGCATATGGTGCACTCTCAGTACAATCTGCTCTGAT<br>GCCGCATAGTTAAGCCAGTATACACTCCGCTATCGCTACGTGACTGGGTCATGGCTGC<br>GCCCCGACACCCGCCAACACCCGCTGACGCGCCCTGACGGGCTTGTCTGCTCCCGGCA<br>TCCGCTTACAGACAAGCTGTGACCGTCTCCGGGAGCTGCATGTGTCAGAGGTTTTCAC<br>CGTCATCACCGAAACGCGCGAGGCAGCAGATCAATTCGCGCGCGAAGGCGAAGCGGC<br>ATGCATAATGTGCCTGTCAAATGGACGAAGCAGGGATTCTGCAAACCCTATGCTACTC<br>CGTCAAGCCGTCAATTGTCTGATTCGTTACCAATTATGACAACTTGACGGCTACATCAT<br>TCACTTTTTCTTCACAACCGGCACGGAACTCGCTCGGGCTGGCCCCGGTGCATTTTTA<br>AATACCCGCGAGAAATAGAGTTGATCGTCAAAACCAACATTGCGACCGACGGTGGCG<br>ATAGGCATCCGGGTGGTGCTCAAAAGCAGCTTCGCCTGGCTGATACGTTGGTCCTCGC<br>GCCAGCTTAAGACGCTAATCCCTAACTGCTGGCGGAAAAGATGTGACAGACGCGACG<br>GCGACAAGCAAACATGCTGTGCGACGCTGGCGATATCAAAATTGCTGTCTGCCAGGTG<br>ATCGCTGATGTACTGACAAGCCTCGCGTACCCGATTATCCATCGGTGGATGGAGCGAC<br>TCGTTAATCGCTTCCATGCGCCGCAGTAACAATTGCTCAAGCAGATTTATCGCCAGCA<br>GCTCCGAATAGCGCCCTTCCCCTTGCCCGGCGTTAATGATTTGCCCAAACAGGTCGCTG<br>AAATGCGGCTGGTGCGCTTCATCCGGGCGAAAGAACCCCGTATTGGCAAATATTGACG<br>GCCAGTTAAGCCATTCATGCCAGTAGGCGCGCGGACGAAAGTAAACCCACTGGTGATA<br>CCATTCGCGAGCCTCCGGATGACGACCGTAGTGATGAATCTCTCCTGGCGGGAACAGC<br>AAAATATCACCCGGTCGGCAAACAAATTCTCGTCCCTGATTTTTCACCACCCCCTGACC

	GCGAATGGTGAGATTGAGAATATAACCTTTCATTCCCAGCGGTCGGTCGATAAAAAAA<br>TCGAGATAACCGTTGGCCTCAATCGGCGTTAAACCCGCCACCAGATGGGCATTAAACG<br>AGTATCCCGGCAGCAGGGGATCATTTTGCGCTTCAGCCATACTTTTCATACTCCCGCCA<br>TTCAGAG
pCDFDuet-mCherry-CS2	GGGGAATTGTGAGCGGATAACAATTCCCCTGTAGAAATAATTTTGTTTAACTTTAATA<br>AGGAGATATACCATGGGCAGCAGCCATCACCATCATCACCACAGCCAGGATCCGAATT<br>CGAGCTCGGCGCGCCTGCAGGTCGACAAGCTTGCGGCCGCATAATGCTTAAGTCGAAC<br>AGAAAGTAATCGTATTGTACACGGCCGCATAATCGAAATTAATACGACTCACTATAGG<br>GGAATTGTGAGCGGATAACAATTCCCCATCTTAGTATATTAGTTAAGTATAAGAAGGA<br>GATATACATATGGTGAGCAAGGGCGAGGAGGATAACATGGCCATCATCAAGGAGTTC<br>ATGCGCTTCAAGGTGCACATGGAGGGCTCCGTGAACGGCCACGAGTTCGAGATCGAG<br>GGCGAGGGCGAGGGCCGCCCCTACGAGGGCACCCAGACCGCCAAGCTGAAGGTGACC<br>AAGGGTGGCCCCCTGCCCTTCGCCTGGGACATCCTGTCCCCTCAGTTCATGTACGGCTC<br>CAAGGCCTACGTGAAGCACCCCGCCGACATCCCCGACTACTTGAAGCTGTCCTTCCCC<br>GAGGGCTTCAAGTGGGAGCGCGTGATGAACTTCGAGGACGGCGGCGTGGTGACCGTG<br>ACCCAGGACTCCTCCCTCCAGGACGGCGAGTTCATCTACAAGGTGAAGCTGCGCGGCA<br>CCAACTTCCCCTCCGACGGCCCCGTAATGCAGAAGAAGACCATGGGCTGGGAGGCCTC<br>CTCCGAGCGGATGTACCCCGAGGACGGCGCCCTGAAGGGCGAGATCAAGCAGAGGCT<br>GAAGCTGAAGGACGGCGGCCACTACGACGCTGAGGTCAAGACCACCTACAAGGCCAA<br>GAAGCCCGTGCAGCTGCCCGGCGCCTACAACGTCAACATCAAGTTGGACATCACCTCC<br>CACAACGAGGACTACACCATCGTGGAACAGTACGAACGCGCCGAGGGCCGCCACTCC<br>ACCGGCGGCATGGACGAGCTGTACAAGACGAGCACCCCAGAGCCCGAAGCTCAATCA<br>ACTGAACAATCATTGACCTGTGAAGGACAAATTATTAGCGGCACTTCAGTTGACGCCA<br>GTGATTTGGTCACAGGAAATGAAATCGGTGAACAGCAACTCATCAGCGGTGACGCCTA<br>TGTTGGCGCGCAGCAGACAGGTTGCCTTCCCACTAGTCCACGCTTCAACCAAACTGGC<br>AATGTTCAGTCAATGGGTTTTAAGAACACCAATCAGCCAGAACAAAACTTTGCACCAG<br>GTGAAGTAATGCCTACTGACTTTAGTATTCAAACCCCAGCTCGCTCGGCTCAGAATCG<br>CATTACAGGTAACGACATTGCGCCCTCAGGTCGCATTACAGGCCCTGGTATGCTGGCA<br>ACCGGCTTGATTACAGGAACCCCCGAATTTAGGCACGCTGCGCGCGAGTTGGTTGGTT<br>CTCCACAACCGATGGCAATGGCGATGGCCAACCGTAATAAGGCTGCTCAAGCACCTGT<br>TGTGCAGCCAGAAGTGGTTGCAACTCAGGAAAAGCCTGAGTTGGTATGTGCACCAAGA<br>AGCGATCAAATGGATCGTGTGAGTGGCGAAGGCAAAGAACGTTGCCACATCACTGGC<br>GATGACTGGTCAGTTAACAAGCACATCACCGGTACAGCCGGTCAATGGGCGAGTGGTC<br>GCAACCCTTCCATGCGCGGTAATGCGCGTGTGGTCGAAACCAGCGCGTTTGCCAATCG<br>CAATGTGCCAAAACCTGAAAAGCCGGGCTCCAAGATCACGGGCAGTAGTGGTAATGA<br>CACCCAAGGTAGTCTGATCACTTACTCCGGCGGCGCGCGCGGTTGATCGAGTCTGGTA<br>AAGAAACCGCTGCTGCGAAATTTGAACGCCAGCACATGGACTCGTCTACTAGCGCAGC<br>TTAATTAACCTAGGCTGCTGCCACCGCTGAGCAATAACTAGCATAACCCCTTGGGGCC<br>TCTAAACGGGTCTTGAGGGGTTTTTTGCTGAAACCTCAGGCATTTGAGAAGCACACGG<br>TCACACTGCTTCCGGTAGTCAATAAACCGGTAAACCAGCAATAGACATAAGCGGCTAT<br>TTAACGACCCTGCCCTGAACCGACGACCGGGTCATCGTGGCCGGATCTTGCGGCCCCT

	CGGCTTGAACGAATTGTTAGACATTATTTGCCGACTACCTTGGTGATCTCGCCTTTCAC<br>GTAGTGGACAAATTCTTCCAACTGATCTGCGCGCGAGGCCAAGCGATCTTCTTCTTGTC<br>CAAGATAAGCCTGTCTAGCTTCAAGTATGACGGGCTGATACTGGGCCGGCAGGCGCTC<br>CATTGCCCAGTCGGCAGCGACATCCTTCGGCGCGATTTTGCCGGTTACTGCGCTGTACC<br>AAATGCGGGACAACGTAAGCACTACATTTCGCTCATCGCCAGCCCAGTCGGGCGGCGA<br>GTTCCATAGCGTTAAGGTTTCATTTAGCGCCTCAAATAGATCCTGTTCAGGAACCGGAT<br>CAAAGAGTTCCTCCGCCGCTGGACCTACCAAGGCAACGCTATGTTCTCTTGCTTTTGTC<br>AGCAAGATAGCCAGATCAATGTCGATCGTGGCTGGCTCGAAGATACCTGCAAGAATGT<br>CATTGCGCTGCCATTCTCCAAATTGCAGTTCGCGCTTAGCTGGATAACGCCACGGAAT<br>GATGTCGTCGTGCACAACAATGGTGACTTCTACAGCGCGGAGAATCTCGCTCTCTCCA<br>GGGGAAGCCGAAGTTTCCAAAAGGTCGTTGATCAAAGCTCGCCGCGTTGTTTCATCAA<br>GCCTTACGGTCACCGTAACCAGCAAATCAATATCACTGTGTGGCTTCAGGCCGCCATC<br>CACTGCGGAGCCGTACAAATGTACGGCCAGCAACGTCGGTTCGAGATGGCGCTCGATG<br>ACGCCAACTACCTCTGATAGTTGAGTCGATACTTCGGCGATCACCGCTTCCCTCATACT<br>CTTCCTTTTTCAATATTATTGAAGCATTTATCAGGGTTATTGTCTCATGAGCGGATACA<br>TATTTGAATGTATTTAGAAAAATAAACAAATAGCTAGCTCACTCGGTCGCTACGCTCC<br>GGGCGTGAGACTGCGGCGGGCGCTGCGGACACATACAAAGTTACCCACAGATTCCGT<br>GGATAAGCAGGGGACTAACATGTGAGGCAAAACAGCAGGGCCGCGCCGGTGGCGTTT<br>TTCCATAGGCTCCGCCCTCCTGCCAGAGTTCACATAAACAGACGCTTTTCCGGTGCATC<br>TGTGGGAGCCGTGAGGCTCAACCATGAATCTGACAGTACGGGCGAAACCCGACAGGA<br>CTTAAAGATCCCCACCGTTTCCGGCGGGTCGCTCCCTCTTGCGCTCTCCTGTTCCGACC<br>CTGCCGTTTACCGGATACCTGTTCCGCCTTTCTCCCTTACGGGAAGTGTGGCGCTTTCT<br>CATAGCTCACACACTGGTATCTCGGCTCGGTGTAGGTCGTTCGCTCCAAGCTGGGCTGT<br>AAGCAAGAACTCCCCGTTCAGCCCGACTGCTGCGCCTTATCCGGTAACTGTTCACTTGA<br>GTCCAACCCGGAAAAGCACGGTAAAACGCCACTGGCAGCAGCCATTGGTAACTGGGA<br>GTTCGCAGAGGATTTGTTTAGCTAAACACGCGGTTGCTCTTGAAGTGTGCGCCAAAGT<br>CCGGCTACACTGGAAGGACAGATTTGGTTGCTGTGCTCTGCGAAAGCCAGTTACCACG<br>GTTAAGCAGTTCCCCAACTGACTTAACCTTCGATCAAACCACCTCCCCAGGTGGTTTTT<br>TCGTTTACAGGGCAAAAGATTACGCGCAGAAAAAAAGGATCTCAAGAAGATCCTTTG<br>ATCTTTTCTACTGAACCGCTCTAGATTTCAGTGCAATTTATCTCTTCAAATGTAGCACCT<br>GAAGTCAGCCCCATACGATATAAGTTGTAATTCTCATGTTAGTCATGCCCCGCGCCCAC<br>CGGAAGGAGCTGACTGGGTTGAAGGCTCTCAAGGGCATCGGTCGAGATCCCGGTGCCT<br>AATGAGTGAGCTAACTTACATTAATTGCGTTGCGCTCACTGCCCGCTTTCCAGTCGGGA<br>AACCTGTCGTGCCAGCTGCATTAATGAATCGGCCAACGCGCGGGGAGAGGCGGTTTGC<br>GTATTGGGCGCCAGGGTGGTTTTTCTTTTCACCAGTGAGACGGGCAACAGCTGATTGC<br>CCTTCACCGCCTGGCCCTGAGAGAGTTGCAGCAAGCGGTCCACGCTGGTTTGCCCCAG<br>CAGGCGAAAATCCTGTTTGATGGTGGTTAACGGCGGGATATAACATGAGCTGTCTTCG<br>GTATCGTCGTATCCCACTACCGAGATGTCCGCACCAACGCGCAGCCCGGACTCGGTAA<br>TGGCGCGCATTGCGCCCAGCGCCATCTGATCGTTGGCAACCAGCATCGCAGTGGGAAC<br>GATGCCCTCATTCAGCATTTGCATGGTTTGTTGAAAACCGGACATGGCACTCCAGTCGC<br>CTTCCCGTTCCGCTATCGGCTGAATTTGATTGCGAGTGAGATATTTATGCCAGCCAGCC

	AGACGCAGACGCGCCGAGACAGAACTTAATGGGCCCGCTAACAGCGCGATTTGCTGG<br>TGACCCAATGCGACCAGATGCTCCACGCCCAGTCGCGTACCGTCTTCATGGGAGAAAA<br>TAATACTGTTGATGGGTGTCTGGTCAGAGACATCAAGAAATAACGCCGGAACATTAGT<br>GCAGGCAGCTTCCACAGCAATGGCATCCTGGTCATCCAGCGGATAGTTAATGATCAGC<br>CCACTGACGCGTTGCGCGAGAAGATTGTGCACCGCCGCTTTACAGGCTTCGACGCCGC<br>TTCGTTCTACCATCGACACCACCACGCTGGCACCCAGTTGATCGGCGCGAGATTTAATC<br>GCCGCGACAATTTGCGACGGCGCGTGCAGGGCCAGACTGGAGGTGGCAACGCCAATC<br>AGCAACGACTGTTTGCCCGCCAGTTGTTGTGCCACGCGGTTGGGAATGTAATTCAGCT<br>CCGCCATCGCCGCTTCCACTTTTTCCCGCGTTTTCGCAGAAACGTGGCTGGCCTGGTTC<br>ACCACGCGGGAAACGGTCTGATAAGAGACACCGGCATACTCTGCGACATCGTATAAC<br>GTTACTGGTTTCACATTCACCACCCTGAATTGACTCTCTTCCGGGCGCTATCATGCCAT<br>ACCGCGAAAGGTTTTGCGCCATTCGATGGTGTCCGGGATCTCGACGCTCTCCCTTATGC<br>GACTCCTGCATTAGGAAATTAATACGACTCACTATA
pCDFDuet-hydA-CS2	GGGGAATTGTGAGCGGATAACAATTCCCCTGTAGAAATAATTTTGTTTAACTTTAATA<br>AGGAGATATACCATGGGCAGCAGCCATCACCATCATCACCACAGCCAGGATCCGGGT<br>ACCGCCTATAAAGTTACCCTGAAAACCCCGAGCGGTGATAAAACCATTGAATGTCCGG<br>CAGATACCTATATTCTGGATGCAGCAGAAGAAGCAGGTCTGGATCTGCCGTATAGCTG<br>TCGTGCCGGTGCATGTAGCAGCTGTGCAGGTAAAGTTGCAGCAGGCACCGTGGATCAG<br>AGCGATCAGAGTTTTCTGGATGATGCACAGATGGGTAATGGTTTTGTTCTGACCTGTGT<br>TGCATATCCGACCAGCGATTGTACCATTCAGACCCATCAAGAAGAGGCACTGTACGGA<br>GGTGGTGGTAGCGGTGGTGGTGGTTCAGGTGGTGGCGGTAGCGCAGCACCGGCAGCT<br>GAAGCACCGCTGAGCCATGTTCAGCAGGCCCTGGCAGAACTGGCAAAACCGAAAGAT<br>GATCCGACACGTAAACATGTGTGTGTTCAGGTTGCTCCGGCAGTTCGTGTTGCCATTGC<br>AGAAACCCTGGGTTTAGCACCGGGTGCAACCACACCGAAACAGCTGGCAGAAGGTCT<br>GCGTCGTCTGGGTTTTGATGAAGTTTTTGATACCCTGTTTGGTGCCGATCTGACCATTA<br>TGGAAGAAGGTAGCGAACTGCTGCACCGTCTGACCGAACATCTGGAAGCACATCCGC<br>ATAGTGATGAACCGCTGCCGATGTTTACCAGCTGTTGTCCTGGTTGGATTGCAATGCTG<br>GAAAAAAGCTATCCGGATCTGATTCCGTATGTTAGCAGCTGCAAAAGTCCGCAGATGA<br>TGCTGGCAGCAATGGTTAAAAGTTATCTGGCCGAAAAAAAAGGGATTGCACCTAAAG<br>ATATGGTGATGGTTAGCATTATGCCGTGTACGCGTAAACAGAGCGAAGCAGATCGTGA<br>TTGGTTTTGTGTTGATGCAGATCCGACGCTGCGTCAGCTGGATCATGTTATTACCACCG<br>TTGAACTGGGCAACATTTTCAAAGAACGTGGTATTAATCTGGCGGAACTGCCTGAAGG<br>TGAATGGGATAATCCGATGGGTGTTGGTAGTGGTGCCGGTGTTCTGTTTGGTACAACC<br>GGTGGTGTTATGGAAGCAGCACTGCGTACCGCATATGAACTGTTTACCGGTACACCGC<br>TGCCTCGTCTGAGCCTGAGCGAAGTTCGTGGTATGGATGGTATTAAAGAAACCAACAT<br>TACCATGGTTCCGGCACCGGGTAGTAAATTTGAAGAACTGCTGAAACATCGTGCCGCA<br>GCACGTGCCGAAGCAGCAGCACATGGTACACCGGGTCCGCTGGCATGGGATGGTGGT<br>GCAGGTTTTACCAGCGAAGATGGTCGTGGTGGTATTACCCTGCGTGTTGCAGTTGCAA<br>ATGGTCTGGGTAATGCAAAAAAACTGATCACCAAAATGCAGGCAGGCGAAGCCAAAT<br>ATGATTTTGTTGAAATTATGGCATGTCCCGCAGGTTGTGTTGGCGGTGGCGGTCAGCCT<br>CGTAGCACCGATAAAGCAATTACACAGAAACGTCAGGCAGCACTGTATAACCTGGAT

	GAAAAAAGCACCCTGCGTCGTAGCCATGAAAATCCGAGCATTCGTGAACTGTATGATA<br>CCTATCTGGGCGAACCGCTGGGTCATAAAGCACATGAGCTGCTGCATACCCATTACGT<br>CGCAGGCGGTGTCGAGGAGAAGGACGAGAAGAAGGAGCTCGGAGCACAAGACTGGC<br>CTCCGTTTTGTACGAGCACCCCAGAGCCCGAAGCTCAATCAACTGAACAATCATTGAC<br>CTGTGAAGGACAAATTATTAGCGGCACTTCAGTTGACGCCAGTGATTTGGTCACAGGA<br>AATGAAATCGGTGAACAGCAACTCATCAGCGGTGACGCCTATGTTGGCGCGCAGCAG<br>ACAGGTTGCCTTCCCACTAGTCCACGCTTCAACCAAACTGGCAATGTTCAGTCAATGG<br>GTTTTAAGAACACCAATCAGCCAGAACAAAACTTTGCACCAGGTGAAGTAATGCCTAC<br>TGACTTTAGTATTCAAACCCCAGCTCGCTCGGCTCAGAATCGCATTACAGGTAACGAC<br>ATTGCGCCCTCAGGTCGCATTACAGGCCCTGGTATGCTGGCAACCGGCTTGATTACAG<br>GAACCCCCGAATTCAGGCACGCTGCGCGCGAGTTGGTTGGTTCTCCACAACCCATGGC<br>AATGGCCATGGCCAACCGTAATAAGGCTGCTCAAGCACCTGTTGTGCAGCCAGAAGTG<br>GTTGCAACTCAGGAAAAGCCTGAGTTGGTATGTGCACCAAGAAGCGATCAAATGGATC<br>GTGTGAGTGGCGAAGGCAAAGAACGTTGCCACATCACTGGCGATGACTGGTCAGTTAA<br>CAAGCACATCACCGGTACAGCCGGTCAATGGGCGAGTGGTCGCAACCCTTCCATGCGC<br>GGTAATGCGCGTGTGGTCGAAACCAGCGCGTTTGCCAATCGCAATGTGCCAAAACCTG<br>AAAAGCCGGGCTCCAAGATCACGGGCAGTAGTGGTAATGACACCCAAGGTAGTCTGA<br>TCACTTACTCCGGCGGCGCGCGCGGTTGATTAAGTAAAGTGTAACGATCCACAGAGGA<br>ACAGGTATGGCTGATTGGGTAACAGGCAAAGTCACTAAAGTGCAGAACTGGACCGAC<br>GCCCTGTTTAGTCTCACCGTTCACGCCCCTGTGCATCCGTTTACCGCCGGGCAATTCAC<br>TAAACTTGGCCTTGAAATTGACGGCGAACGCGTCCAACGCGCCTACTCCTATGTTAAC<br>TCGCCCGATAATCCCGATCTGGAGTTTTACCTGGTCACCGTCCCCGATGGCAAATTAAG<br>CCCACGATTGGCGGCACTGAAACCGGGCGATGAAGTGCAGGTGGTTAGCGAAGCAGC<br>TGGCTTCTTTGTTCTGGATGAAGTACCAGATTGCGAAACGCTATGGATGCTGGCAACC<br>GGTACAGCGATTGGCCCTTATTTATCGATTCTGCAACTAGGCAAAGATTTAGATCGCTT<br>CAAAAATCTGGTCCTGGTTCACGCCGCACGTTATGCCGCCGACTTAAGCTATTTGCCAC<br>TGATGCAAGAACTGGAAAAACGCTACGAAGGCAAACTGCGCATTCAGACGGTGGTCA<br>GTCGGGAAACGGCAGCGGGGTCGCTCACCGGACGGATACCGGCATTAATTGAAAGTG<br>GGGAACTGGAAAGCGCGATTGGCCTGCCGATGAATAAAGAAACCAGCCATGTGATGC<br>TGTGCGGCAATCCACAGATGGTGCGCGATACACAACAGTTGCTGAAAGAGACCCGGC<br>AGATGACGAAACATTTACGTCGCCGACCGGGCCATATGACAGCGGAGCATTACTGGCA<br>CCATCATCACCACCATGGTAGCGGTAGTGGACCGTTTTGTACGAGCACCCCAGAGCCC<br>GAAGCTCAATCAACTGAACAATCATTGACCTGTGAAGGACAAATTATTAGCGGCACTT<br>CAGTTGACGCCAGTGATTTGGTCACAGGAAATGAAATCGGTGAACAGCAACTCATCAG<br>CGGTGACGCCTATGTTGGCGCGCAGCAGACAGGTTGCCTTCCCACTAGTCCACGCTTC<br>AACCAAACTGGCAATGTTCAGTCAATGGGTTTTAAGAACACCAATCAGCCAGAACAAA<br>ACTTTGCACCAGGTGAAGTAATGCCTACTGACTTTAGTATTCAAACCCCAGCTCGCTCG<br>GCTCAGAATCGCATTACAGGTAACGACATTGCGCCCTCAGGTCGCATTACAGGCCCTG<br>GTATGCTGGCAACCGGCTTGATTACAGGAACCCCCGAATTCAGGCACGCTGCGCGCGA<br>GTTGGTTGGTTCTCCACAACCCATGGCAATGGCCATGGCCAACCGTAATAAGGCTGCT<br>CAAGCACCTGTTGTGCAGCCAGAAGTGGTTGCAACTCAGGAAAAGCCTGAGTTGGTAT

	GTGCACCAAGAAGCGATCAAATGGATCGTGTGAGTGGCGAAGGCAAAGAACGTTGCC<br>ACATCACTGGCGATGACTGGTCAGTTAACAAGCACATCACCGGTACAGCCGGTCAATG<br>GGCGAGTGGTCGCAACCCTTCCATGCGCGGTAATGCGCGTGTGGTCGAAACCAGCGCG<br>TTTGCCAATCGCAATGTGCCAAAACCTGAAAAGCCGGGCTCCAAGATCACGGGCAGTA<br>GTGGTAATGACACCCAAGGTAGTCTGATCACTTACTCCGGCGGCGCGCGCGGTTGATT<br>AAGTAAAGTGTAACGATCCCTGCAGGTCGACAAGCTTGCGGCCAGCATAATGCTTAAG<br>TCGAACAGAAAGTAATCGTATTGTACACGGCCGCATAATCGAAATTAATACGACTCAC<br>TATAGGGGAATTGTGAGCGGATAACAATTCCCCATCTTAGTATATTAGTTAAGTATAA<br>GAAGGAGATATACATATGGCAGATCTCAATTGGATAAGCACACACGAGCATCACTCCA<br>TTACACTTTCGGACTACAATCCCAACGTCAACTTTATCGACGATAAAGCGATTTGGCA<br>GACCATTGAAGACGCCAGTGATCCAAGTCGCGAGCAAGTTCTCGCCATTCTCGACAAG<br>GCGCGCCAGTGTGAAGGCTTAAGCATTAGCGAGACCGCCCTTTTGCTGCAAAACCAAG<br>ATAAGACCTTGGATGAAATGCTTTTTAGCGTCGCCCGTGAGATTAAAAACACTATTTA<br>CGGCAACCGTATTGTGATGTTTGCACCGCTGTATGTATCGAATCATTGCGCCAACAGTT<br>GTAGTTATTGCGGCTTTAACGCCGATAACCATGAGCTCAAACGTAAAACCTTAAAACA<br>GGATGAGATCCGCCAAGAGGTTGCGATCCTTGAAGAAATGGGCCACAAGCGGATCCTT<br>GCAGTCTATGGCGAACATCCTCGCAACAATGTGCAAGCCATTGTTGAAAGTATTCAAA<br>CCATGTACAGCGTTAAGCAGGGCAAGGGCGGAGAAATACGCCGTATCAACGTCAACT<br>GCGCGCCAATGAGTGTGGAGGACTTTAAGCAACTTAAAACCGCGGCGATAGGCACTTA<br>TCAATGCTTCCAAGAAACCTATCATCAAGACACCTACAGCCAAGTCCATCTTAAAGGT<br>AAAAAAACCGACTTTTTATACCGCCTCTACGCCATGCACAGGGCGATGGAAGCAGGAA<br>TTGACGATGTCGGCATTGGCGCCCTCTTTGGCCTGTATGATCATAGATTCGAGCTCCTT<br>GCCATGCTCACCCATGTTCAGCAACTCGAAAAAGACTGTGGCGTTGGCCCACACACTA<br>TCTCCTTTCCGCGGATTGAACCCGCCCATGGCTCTGCTATCAGTGAAAAGCCGCCCTAT<br>GAGGTCGATGATGACTGCTTTAAGCGCATTGTTGCCATCACTCGCCTTGCCGTGCCTTA<br>TACAGGGTTAATTATGAGCACGCGGGAAAGTGCAGCGCTGCGCAAAGAACTATTAGA<br>ACTCGGGGTTTCACAAATCAGCGCAGGCTCGCGTACCGCGCCGGGTGGATATCAAGAC<br>AGCAAACAAAATCAACATGATGCCGAGCAATTCAGCCTTGGTGACCACCGAGAAATG<br>GACGAAATCATCTATGAATTAGTCACCGACTCGGATGCCATCCCCTCCTTCTGCACTGG<br>CTGTTACCGCAAAGGGCGAACTGGCGATCATTTTATGGGATTAGCCAAACAGCAGTTT<br>ATTGGTAAATTCTGCCAGCCCAATGCATTGATCACCTTTAAGGAATATTTGAACGATTA<br>CGCCAGTGAAAAGACCCGCGAGGCTGGCAATGCGCTGATAGAGCGAGAGCTGGCTAA<br>AATGAGCCCGTCACGGGCACGCAATGTGCGCGGCTGTTTGCAAAAAACCGATGCGGGT<br>GAACGGGATATCTATCTGTAAATGGCATCTTATTTATTTCTGAGCCCGGCGTGCAGTT<br>GCATTTTGGCCTTAACACTAACGCCTTACTGAATGCGATAAAACGCGCTGATGCAATC<br>GTGCTACACGCGGCGATTTACAGTAATTTTGCCGATAATGCCGTGGGGCAATTGTTATT<br>ACAGCGGCTACAAAACGCCAGCTTAAAACAACTCACCCTGATTAAACTCGAACCCACC<br>CGTCCGTGGCAGGATGAGTTTGCCACCATCCTAAGGCCCGAGATGCCGCTGCAGGACA<br>TTGAGCAGCTGTATGATATTTCCCGCCACTGGTGCGCCGAGCTTAAAACGCAGTTTCCA<br>GAGGCAGTCAATCTCATGTCGACCCAAGCGTTACCACTGCAACCCATACTGCTGATTG<br>GCGATAAACTCTTTATTGGTCACTACGCCCACAGTAATTGCACCTCGGCTCAAGGACTT

	TGGCTGGAGTTTGATATCCTTGGTTTAGGCATTGCGCCAAACACCTTAATCGATTGGTT<br>TGATACTGGCGTCCCCAGTGAGCAAAACTCAGCGGTGGCAATTGCCCTTGGCCGTTAT<br>GTTGAAGAATGCCGCCGCGCAGTGGGCGATCTTTGGTATCAGAGCTTGATTGAGCTAG<br>ATAAAGGGTTTAACAGATGATCACTCGCCCTAGCCCGCCGGCGCCAGTCACTCAGCCA<br>ACGTCCGTTAAACCGACGTTGCTTAACACAGTGTTTTCCTATGCCGAAATCCTTTCGCT<br>ACTTCAGGGGCAAGACGACGAATGGCTATTTAGTCGCGCAAAGCTCGCCACTGAGTTG<br>GAATTTAATCAACAGGTGTATTTACGCGGCATTGTCGAATTTTCGAATCACTGTCGTAA<br>CCACTGCCATTACTGTGGTTTACGCACCGAAAATCGGCAGGTAACACGCTATCGGCTC<br>TCGAACGAAGAAATCCTTAACGCAGTGGATAGCATAGCCGAGCTGGGACTAGGTACT<br>GTGGTGCTGCAATCGGGGGATGACTTTAACTACAGCGGCAATCGTATTAGTACCCTTA<br>TTACTGAAATTAAACGTCACCACAATCTAGCAATCACTCTGTCACTGGGTGATCGCAA<br>ACATCAAGAGCTGGAAAAATGGCGCGAAGCGGGCGCAGATCGCTATTTGCTCAAAAT<br>GGAAACCTTCGACCGCGCCCTCTTTGCTCAGTGTCGCCCTAAAGCCAATTTTGACGAGC<br>GCATTGCAAGGCTCAATTATCTTAAGTCACTGGGATATCAAACTGGCTCAGGAATTAT<br>TGTCGATTTACCGGGAATGACGGATGCAATCTTAGCCCGTGATATTCAGCATTTATCTG<br>AGTTGCAGCTCGATATGCTCGCCTGCGGCCCCTTTATCGCCCATCACCAAACGCCGTTT<br>ACGACTTCGCCCAATGGCAGCGCGCTTAAGAGTCACAGGGTGAGCGCTATTTTGCGAC<br>TGATGAATCCGGGGGCGAATATTCCCGCCACCAGTTCACTCGACGCCCTCGATAAGGG<br>CGCAAGAGAGCAGGCACTCAAACGGGGATGTAATGTGATTATGCCTTCCTTTACACCA<br>ACAAAGGTCAGTGGCGATTACAGCATCTATCCAGGAAAAAACCAGCAGCAACACCCC<br>GCAGCAGAGCGACTTAACCAAGTCTGCCAGCAAATTCAACGCCATGGGCTAATACCCT<br>CCTTTAGCCGTGGTGATTCAAAAAGGACTCAATATGTGTCAAGGCATTAATCCACAAG<br>AACATTCCCAAGCCCAATCGGCGCCAAGAGGGATGCGTTACCATATCGCCTTGGTCGG<br>GCGGCGTAATTCGGGGAAATCCTCCTTACTCAATATGCTTGCAGGGCAGCAAATCTCG<br>ATAGTATCCGATATAAAAGGCACGACGACAGATGCCGTGGCCAAGGCTTATGAGTTAC<br>AGCCCTTGGGACCTGTAACCTTTTACGATACCGCTGGCATCGACGATGAAGGGACATT<br>AGGTGCAATGCGAGTGAGCGCCACTCGTAGAGTGTTGTTCCGCTCCGATATGGCACTC<br>TTAGTCGTTGATGAGCAGGGACTATGCCCCTCAGACATGGCATTAATAGACGAAATAC<br>GCCAATTGCAGATGCCAATACTTATGGTATTCAATAAGGCCGATATCTGCACACCCAA<br>AGCTGAGGATATTGCCTTTTGCCAGAATCAATCCTTACCCTTTATTGTCGTTTCTGCCG<br>CCACAGGTCTTGCGGGTAAACAGCTTAAGCAGTTGATGGTCGAATTAGCGCCCGCGGA<br>ATATAAGCAGGAGCCGCTCCTAGCGGGCGACCTCTATCAAGCGGGGGACGTTATTCTC<br>TGCGTCGTCCCTATCGATATGGCCGCACCTAAAGGGCGTCTGATCTTGCCACAGGTGC<br>AAATACTGCGTGAGGCGCTCGATCGCAGCGCGATCGCTATGGTCGTCAAAGAAACAG<br>AACTCGCGCAGGCATTATCTGTCGTTACGCCTAAACTGGTGATTTCCGACGCCCAAGC<br>GATTAAGCAAGTCGCCGCCATCGTGCCAGACGCCGTCCCTCTCACCACCTTCTCGACCC<br>TGTTTGCCCGTTTTAAAGGCGATTTAGCTGCACTCGCCACTGGAGCTGATGCCCTAGAT<br>ACTCTGCAAGATGGCGACAAAGTGCTGATCAGTGAAGCCTGTAGCCACAATGTACAGG<br>AAGATGATATTGGCCGAGTTAAACTCCCCCGCTGGATCAATAGCTACACAGGCAAACA<br>ACTCGAGTTTGTAGTCACATCAGGACACGACTTTCCCAATGATTTAGAGCAATATGCC<br>CTCGTTATTCACTGCGGCGCTTGTATGTTTAATCGCAATGAAATGCTCCGTCGCATCCG

	CGAATGTCAACGGCGGCAGGTGCCCATCACTAACTATGGGGTGGCGATTTCTAAATTA
	CAGGGCGTGCTGCCTCGAGTGCTTACGCCTTTTAACCGCAATCCTCAGCAATAGTAAG
	TCTGGTAAAGAAACCGCTGCTGCGAAATTTGAACGCCAGCACATGGACTCGTCTACTA
	GCGCAGCTTAATTAACCTAGGCTGCTGCCACCGCTGAGCAATAACTAGCATAACCCCT
	TGGGGCCTCTAAACGGGTCTTGAGGGGTTTTTTGCTGAAACCTCAGGCATTTGAGAAG
	CACACGGTCACACTGCTTCCGGTAGTCAATAAACCGGTAAACCAGCAATAGACATAAG
	CGGCTATTTAACGACCCTGCCCTGAACCGACGACCGGGTCATCGTGGCCGGATCTTGC
	GGCCCCTCGGCTTGAACGAATTGTTAGACATTATTTGCCGACTACCTTGGTGATCTCGC
	CTTTCACGTAGTGGACAAATTCTTCCAACTGATCTGCGCGCGAGGCCAAGCGATCTTCT
	TCTTGTCCAAGATAAGCCTGTCTAGCTTCAAGTATGACGGGCTGATACTGGGCCGGCA
	GGCGCTCCATTGCCCAGTCGGCAGCGACATCCTTCGGCGCGATTTTGCCGGTTACTGCG
	CTGTACCAAATGCGGGACAACGTAAGCACTACATTTCGCTCATCGCCAGCCCAGTCGG
	GCGGCGAGTTCCATAGCGTTAAGGTTTCATTTAGCGCCTCAAATAGATCCTGTTCAGG
	AACCGGATCAAAGAGTTCCTCCGCCGCTGGACCTACCAAGGCAACGCTATGTTCTCTT
	GCTTTTGTCAGCAAGATAGCCAGATCAATGTCGATCGTGGCTGGCTCGAAGATACCTG
	CAAGAATGTCATTGCGCTGCCATTCTCCAAATTGCAGTTCGCGCTTAGCTGGATAACGC
	CACGGAATGATGTCGTCGTGCACAACAATGGTGACTTCTACAGCGCGGAGAATCTCGC
	TCTCTCCAGGGGAAGCCGAAGTTTCCAAAAGGTCGTTGATCAAAGCTCGCCGCGTTGT
	TTCATCAAGCCTTACGGTCACCGTAACCAGCAAATCAATATCACTGTGTGGCTTCAGG
	CCGCCATCCACTGCGGAGCCGTACAAATGTACGGCCAGCAACGTCGGTTCGAGATGGC
	GCTCGATGACGCCAACTACCTCTGATAGTTGAGTCGATACTTCGGCGATCACCGCTTCC
	CTCATACTCTTCCTTTTTCAATATTATTGAAGCATTTATCAGGGTTATTGTCTCATGAGC
	GGATACATATTTGAATGTATTTAGAAAAATAAACAAATAGCTAGCTCACTCGGTCGCT
	ACGCTCCGGGCGTGAGACTGCGGCGGGCGCTGCGGACACATACAAAGTTACCCACAG
	ATTCCGTGGATAAGCAGGGGACTAACATGTGAGGCAAAACAGCAGGGCCGCGCCGGT
	GGCGTTTTTCCATAGGCTCCGCCCTCCTGCCAGAGTTCACATAAACAGACGCTTTTCCG
	GTGCATCTGTGGGAGCCGTGAGGCTCAACCATGAATCTGACAGTACGGGCGAAACCCG
	ACAGGACTTAAAGATCCCCACCGTTTCCGGCGGGTCGCTCCCTCTTGCGCTCTCCTGTT
	CCGACCCTGCCGTTTACCGGATACCTGTTCCGCCTTTCTCCCTTACGGGAAGTGTGGCG
	CTTTCTCATAGCTCACACACTGGTATCTCGGCTCGGTGTAGGTCGTTCGCTCCAAGCTG
	GGCTGTAAGCAAGAACTCCCCGTTCAGCCCGACTGCTGCGCCTTATCCGGTAACTGTTC
	ACTTGAGTCCAACCCGGAAAAGCACGGTAAAACGCCACTGGCAGCAGCCATTGGTAA
	CTGGGAGTTCGCAGAGGATTTGTTTAGCTAAACACGCGGTTGCTCTTGAAGTGTGCGC
	CAAAGTCCGGCTACACTGGAAGGACAGATTTGGTTGCTGTGCTCTGCGAAAGCCAGTT
	ACCACGGTTAAGCAGTTCCCCAACTGACTTAACCTTCGATCAAACCACCTCCCCAGGT
	GGTTTTTTCGTTTACAGGGCAAAAGATTACGCGCAGAAAAAAAGGATCTCAAGAAGAT
	CCTTTGATCTTTTCTACTGAACCGCTCTAGATTTCAGTGCAATTTATCTCTTCAAATGTA
	GCACCTGAAGTCAGCCCCATACGATATAAGTTGTAATTCTCATGTTAGTCATGCCCCGC
	GCCCACCGGAAGGAGCTGACTGGGTTGAAGGCTCTCAAGGGCATCGGTCGAGATCCC
	GGTGCCTAATGAGTGAGCTAACTTACATTAATTGCGTTGCGCTCACTGCCCGCTTTCCA
	GTCGGGAAACCTGTCGTGCCAGCTGCATTAATGAATCGGCCAACGCGCGGGGAGAGG

	CGGTTTGCGTATTGGGCGCCAGGGTGGTTTTTCTTTTCACCAGTGAGACGGGCAACAG<br>CTGATTGCCCTTCACCGCCTGGCCCTGAGAGAGTTGCAGCAAGCGGTCCACGCTGGTT<br>TGCCCCAGCAGGCGAAAATCCTGTTTGATGGTGGTTAACGGCGGGATATAACATGAGC<br>TGTCTTCGGTATCGTCGTATCCCACTACCGAGATGTCCGCACCAACGCGCAGCCCGGA<br>CTCGGTAATGGCGCGCATTGCGCCCAGCGCCATCTGATCGTTGGCAACCAGCATCGCA<br>GTGGGAACGATGCCCTCATTCAGCATTTGCATGGTTTGTTGAAAACCGGACATGGCAC<br>TCCAGTCGCCTTCCCGTTCCGCTATCGGCTGAATTTGATTGCGAGTGAGATATTTATGC<br>CAGCCAGCCAGACGCAGACGCGCCGAGACAGAACTTAATGGGCCCGCTAACAGCGCG<br>ATTTGCTGGTGACCCAATGCGACCAGATGCTCCACGCCCAGTCGCGTACCGTCTTCATG<br>GGAGAAAATAATACTGTTGATGGGTGTCTGGTCAGAGACATCAAGAAATAACGCCGG<br>AACATTAGTGCAGGCAGCTTCCACAGCAATGGCATCCTGGTCATCCAGCGGATAGTTA<br>ATGATCAGCCCACTGACGCGTTGCGCGAGAAGATTGTGCACCGCCGCTTTACAGGCTT<br>CGACGCCGCTTCGTTCTACCATCGACACCACCACGCTGGCACCCAGTTGATCGGCGCG<br>AGATTTAATCGCCGCGACAATTTGCGACGGCGCGTGCAGGGCCAGACTGGAGGTGGC<br>AACGCCAATCAGCAACGACTGTTTGCCCGCCAGTTGTTGTGCCACGCGGTTGGGAATG<br>TAATTCAGCTCCGCCATCGCCGCTTCCACTTTTTCCCGCGTTTTCGCAGAAACGTGGCT<br>GGCCTGGTTCACCACGCGGGAAACGGTCTGATAAGAGACACCGGCATACTCTGCGACA<br>TCGTATAACGTTACTGGTTTCACATTCACCACCCTGAATTGACTCTCTTCCGGGCGCTA<br>TCATGCCATACCGCGAAAGGTTTTGCGCCATTCGATGGTGTCCGGGATCTCGACGCTCT<br>CCCTTATGCGACTCCTGCATTAGGAAATTAATACGACTCACTATA
pCDFDueT-<br>*hydA-*<br>*hydGxEF-CS2*	GGGGAATTGTGAGCGGATAACAATTCCCCTGTAGAAATAATTTTGTTTAACTTTAATA<br>AGGAGATATACCATGGGCAGCAGCCATCACCATCATCACCACAGCCAGGATCCGGCA<br>GCACCGGCAGCTGAAGCACCGCTGAGCCATGTTCAGCAGGCCCTGGCAGAACTGGCA<br>AAACCGAAAGATGATCCGACACGTAAACATGTGTGTGTTCAGGTTGCTCCGGCAGTTC<br>GTGTTGCCATTGCAGAAACCCTGGGTTTAGCACCGGGTGCAACCACACCGAAACAGCT<br>GGCAGAAGGTCTGCGTCGTCTGGGTTTTGATGAAGTTTTTGATACCCTGTTTGGTGCCG<br>ATCTGACCATTATGGAAGAAGGTAGCGAACTGCTGCACCGTCTGACCGAACATCTGGA<br>AGCACATCCGCATAGTGATGAACCGCTGCCGATGTTTACCAGCTGTTGTCCTGGTTGG<br>ATTGCAATGCTGGAAAAAAGCTATCCGGATCTGATTCCGTATGTTAGCAGCTGCAAAA<br>GTCCGCAGATGATGCTGGCAGCAATGGTTAAAAGTTATCTGGCCGAAAAAAAAGGGA<br>TTGCACCTAAAGATATGGTGATGGTTAGCATTATGCCGTGTACGCGTAAACAGAGCGA<br>AGCAGATCGTGATTGGTTTTGTGTTGATGCAGATCCGACGCTGCGTCAGCTGGATCAT<br>GTTATTACCACCGTTGAACTGGGCAACATTTTCAAAGAACGTGGTATTAATCTGGCGG<br>AACTGCCTGAAGGTGAATGGGATAATCCGATGGGTGTTGGTAGTGGTGCCGGTGTTCT<br>GTTTGGTACAACCGGTGGTGTTATGGAAGCAGCACTGCGTACCGCATATGAACTGTTT<br>ACCGGTACACCGCTGCCTCGTCTGAGCCTGAGCGAAGTTCGTGGTATGGATGGTATTA<br>AAGAAACCAACATTACCATGGTTCCGGCACCGGGTAGTAAATTTGAAGAACTGCTGAA<br>ACATCGTGCCGCAGCACGTGCCGAAGCAGCAGCACATGGTACACCGGGTCCGCTGGC<br>ATGGGATGGTGGTGCAGGTTTTACCAGCGAAGATGGTCGTGGTGGTATTACCCTGCGT<br>GTTGCAGTTGCAAATGGTCTGGGTAATGCAAAAAAACTGATCACCAAAATGCAGGCA<br>GGCGAAGCCAAATATGATTTTGTTGAAATTATGGCATGTCCCGCAGGTTGTGTTGGCG

	GTGGCGGTCAGCCTCGTAGCACCGATAAAGCAATTACACAGAAACGTCAGGCAGCAC<br>TGTATAACCTGGATGAAAAAAGCACCCTGCGTCGTAGCCATGAAAATCCGAGCATTCG<br>TGAACTGTATGATACCTATCTGGGCGAACCGCTGGGTCATAAAGCACATGAGCTGCTG<br>CATACCCATTACGTCGCAGGCGGTGTCGAGGAGAAGGACGAGAAGAAGGAGCTCGGA<br>GCACAAGACTGGCCTCCGTTTTGTACGAGCACCCCAGAGCCCGAAGCTCAATCAACTG<br>AACAATCATTGACCTGTGAAGGACAAATTATTAGCGGCACTTCAGTTGACGCCAGTGA<br>TTTGGTCACAGGAAATGAAATCGGTGAACAGCAACTCATCAGCGGTGACGCCTATGTT<br>GGCGCGCAGCAGACAGGTTGCCTTCCCACTAGTCCACGCTTCAACCAAACTGGCAATG<br>TTCAGTCAATGGGTTTTAAGAACACCAATCAGCCAGAACAAAACTTTGCACCAGGTGA<br>AGTAATGCCTACTGACTTTAGTATTCAAACCCCAGCTCGCTCGGCTCAGAATCGCATTA<br>CAGGTAACGACATTGCGCCCTCAGGTCGCATTACAGGCCCTGGTATGCTGGCAACCGG<br>CTTGATTACAGGAACCCCCGAATTCAGGCACGCTGCGCGCGAGTTGGTTGGTTCTCCA<br>CAACCCATGGCAATGGCCATGGCCAACCGTAATAAGGCTGCTCAAGCACCTGTTGTGC<br>AGCCAGAAGTGGTTGCAACTCAGGAAAAGCCTGAGTTGGTATGTGCACCAAGAAGCG<br>ATCAAATGGATCGTGTGAGTGGCGAAGGCAAAGAACGTTGCCACATCACTGGCGATG<br>ACTGGTCAGTTAACAAGCACATCACCGGTACAGCCGGTCAATGGGCGAGTGGTCGCAA<br>CCCTTCCATGCGCGGTAATGCGCGTGTGGTCGAAACCAGCGCGTTTGCCAATCGCAAT<br>GTGCCAAAACCTGAAAAGCCGGGCTCCAAGATCACGGGCAGTAGTGGTAATGACACC<br>CAAGGTAGTCTGATCACTTACTCCGGCGGCGCGCGCGGTTGATTAAGTAAAGTGTAAC<br>GATCGCATAATGCTTAAGTCGAACAGAAAGTAATCGTATTGTACACGGCCGCATAATC<br>GAAATTAATACGACTCACTATAGGGGAATTGTGAGCGGATAACAATTCCCCATCTTAG<br>TATATTAGTTAAGTATAAGAAGGAGATATACATATGGCAGATCTCAATTGGATAAGCA<br>CACACGAGCATCACTCCATTACACTTTCGGACTACAATCCCAACGTCAACTTTATCGAC<br>GATAAAGCGATTTGGCAGACCATTGAAGACGCCAGTGATCCAAGTCGCGAGCAAGTTC<br>TCGCCATTCTCGACAAGGCGCGCCAGTGTGAAGGCTTAAGCATTAGCGAGACCGCCCT<br>TTTGCTGCAAAACCAAGATAAGACCTTGGATGAAATGCTTTTTAGCGTCGCCCGTGAG<br>ATTAAAAACACTATTTACGGCAACCGTATTGTGATGTTTGCACCGCTGTATGTATCGAA<br>TCATTGCGCCAACAGTTGTAGTTATTGCGGCTTTAACGCCGATAACCATGAGCTCAAA<br>CGTAAAACCTTAAAACAGGATGAGATCCGCCAAGAGGTTGCGATCCTTGAAGAAATG<br>GGCCACAAGCGGATCCTTGCAGTCTATGGCGAACATCCTCGCAACAATGTGCAAGCCA<br>TTGTTGAAAGTATTCAAACCATGTACAGCGTTAAGCAGGGCAAGGGCGGAGAAATAC<br>GCCGTATCAACGTCAACTGCGCGCCAATGAGTGTGGAGGACTTTAAGCAACTTAAAAC<br>CGCGGCGATAGGCACTTATCAATGCTTCCAAGAAACCTATCATCAAGACACCTACAGC<br>CAAGTCCATCTTAAAGGTAAAAAAACCGACTTTTTATACCGCCTCTACGCCATGCACA<br>GGGCGATGGAAGCAGGAATTGACGATGTCGGCATTGGCGCCCTCTTTGGCCTGTATGA<br>TCATAGATTCGAGCTCCTTGCCATGCTCACCCATGTTCAGCAACTCGAAAAAGACTGT<br>GGCGTTGGCCCACACACTATCTCCTTTCCGCGGATTGAACCCGCCCATGGCTCTGCTAT<br>CAGTGAAAAGCCGCCCTATGAGGTCGATGATGACTGCTTTAAGCGCATTGTTGCCATC<br>ACTCGCCTTGCCGTGCCTTATACAGGGTTAATTATGAGCACGCGGGAAAGTGCAGCGC<br>TGCGCAAAGAACTATTAGAACTCGGGGTTTCACAAATCAGCGCAGGCTCGCGTACCGC<br>GCCGGGTGGATATCAAGACAGCAAACAAAATCAACATGATGCCGAGCAATTCAGCCT

	TGGTGACCACCGAGAAATGGACGAAATCATCTATGAATTAGTCACCGACTCGGATGCC<br>ATCCCCTCCTTCTGCACTGGCTGTTACCGCAAAGGGCGAACTGGCGATCATTTTATGGG<br>ATTAGCCAAACAGCAGTTTATTGGTAAATTCTGCCAGCCCAATGCATTGATCACCTTTA<br>AGGAATATTTGAACGATTACGCCAGTGAAAAGACCCGCGAGGCTGGCAATGCGCTGA<br>TAGAGCGAGAGCTGGCTAAAATGAGCCCGTCACGGGCACGCAATGTGCGCGGCTGTTT<br>GCAAAAAACCGATGCGGGTGAACGGGATATCTATCTGTAAATGGCATCTTATTTATTT<br>TCTGAGCCCGGCGTGCAGTTGCATTTTGGCCTTAACACTAACGCCTTACTGAATGCGAT<br>AAAACGCGCTGATGCAATCGTGCTACACGCGGCGATTTACAGTAATTTTGCCGATAAT<br>GCCGTGGGGCAATTGTTATTACAGCGGCTACAAAACGCCAGCTTAAAACAACTCACCC<br>TGATTAAACTCGAACCCACCCGTCCGTGGCAGGATGAGTTTGCCACCATCCTAAGGCC<br>CGAGATGCCGCTGCAGGACATTGAGCAGCTGTATGATATTTCCCGCCACTGGTGCGCC<br>GAGCTTAAAACGCAGTTTCCAGAGGCAGTCAATCTCATGTCGACCCAAGCGTTACCAC<br>TGCAACCCATACTGCTGATTGGCGATAAACTCTTTATTGGTCACTACGCCCACAGTAAT<br>TGCACCTCGGCTCAAGGACTTTGGCTGGAGTTTGATATCCTTGGTTTAGGCATTGCGCC<br>AAACACCTTAATCGATTGGTTTGATACTGGCGTCCCCAGTGAGCAAAACTCAGCGGTG<br>GCAATTGCCCTTGGCCGTTATGTTGAAGAATGCCGCCGCGCAGTGGGCGATCTTTGGT<br>ATCAGAGCTTGATTGAGCTAGATAAAGGGTTTAACAGATGATCACTCGCCCTAGCCCG<br>CCGGCGCCAGTCACTCAGCCAACGTCCGTTAAACCGACGTTGCTTAACACAGTGTTTTC<br>CTATGCCGAAATCCTTTCGCTACTTCAGGGGCAAGACGACGAATGGCTATTTAGTCGC<br>GCAAAGCTCGCCACTGAGTTGGAATTTAATCAACAGGTGTATTTACGCGGCATTGTCG<br>AATTTTCGAATCACTGTCGTAACCACTGCCATTACTGTGGTTTACGCACCGAAAATCGG<br>CAGGTAACACGCTATCGGCTCTCGAACGAAGAAATCCTTAACGCAGTGGATAGCATAG<br>CCGAGCTGGGACTAGGTACTGTGGTGCTGCAATCGGGGGATGACTTTAACTACAGCGG<br>CAATCGTATTAGTACCCTTATTACTGAAATTAAACGTCACCACAATCTAGCAATCACTC<br>TGTCACTGGGTGATCGCAAACATCAAGAGCTGGAAAAATGGCGCGAAGCGGGCGCAG<br>ATCGCTATTTGCTCAAAATGGAAACCTTCGACCGCGCCCTCTTTGCTCAGTGTCGCCCT<br>AAAGCCAATTTTGACGAGCGCATTGCAAGGCTCAATTATCTTAAGTCACTGGGATATC<br>AAACTGGCTCAGGAATTATTGTCGATTTACCGGGAATGACGGATGCAATCTTAGCCCG<br>TGATATTCAGCATTTATCTGAGTTGCAGCTCGATATGCTCGCCTGCGGCCCCTTTATCG<br>CCCATCACCAAACGCCGTTTACGACTTCGCCCAATGGCAGCGCGCTTAAGAGTCACAG<br>GGTGAGCGCTATTTTGCGACTGATGAATCCGGGGGCGAATATTCCCGCCACCAGTTCA<br>CTCGACGCCCTCGATAAGGGCGCAAGAGAGCAGGCACTCAAACGGGGATGTAATGTG<br>ATTATGCCTTCCTTTACACCAACAAAGGTCAGTGGCGATTACAGCATCTATCCAGGAA<br>AAAACCAGCAGCAACACCCCGCAGCAGAGCGACTTAACCAAGTCTGCCAGCAAATTC<br>AACGCCATGGGCTAATACCCTCCTTTAGCCGTGGTGATTCAAAAAGGACTCAATATGT<br>GTCAAGGCATTAATCCACAAGAACATTCCCAAGCCCAATCGGCGCCAAGAGGGATGC<br>GTTACCATATCGCCTTGGTCGGGCGGCGTAATTCGGGGAAATCCTCCTTACTCAATATG<br>CTTGCAGGGCAGCAAATCTCGATAGTATCCGATATAAAAGGCACGACGACAGATGCC<br>GTGGCCAAGGCTTATGAGTTACAGCCCTTGGGACCTGTAACCTTTTACGATACCGCTG<br>GCATCGACGATGAAGGGACATTAGGTGCAATGCGAGTGAGCGCCACTCGTAGAGTGTT<br>GTTCCGCTCCGATATGGCACTCTTAGTCGTTGATGAGCAGGGACTATGCCCCTCAGAC

	ATGGCATTAATAGACGAAATACGCCAATTGCAGATGCCAATACTTATGGTATTCAATA<br>AGGCCGATATCTGCACACCCAAAGCTGAGGATATTGCCTTTTGCCAGAATCAATCCTT<br>ACCCTTTATTGTCGTTTCTGCCGCCACAGGTCTTGCGGGTAAACAGCTTAAGCAGTTGA<br>TGGTCGAATTAGCGCCCGCGGAATATAAGCAGGAGCCGCTCCTAGCGGGCGACCTCTA<br>TCAAGCGGGGGACGTTATTCTCTGCGTCGTCCCTATCGATATGGCCGCACCTAAAGGG<br>CGTCTGATCTTGCCACAGGTGCAAATACTGCGTGAGGCGCTCGATCGCAGCGCGATCG<br>CTATGGTCGTCAAAGAAACAGAACTCGCGCAGGCATTATCTGTCGTTACGCCTAAACT<br>GGTGATTTCCGACGCCCAAGCGATTAAGCAAGTCGCCGCCATCGTGCCAGACGCCGTC<br>CCTCTCACCACCTTCTCGACCCTGTTTGCCCGTTTTAAAGGCGATTTAGCTGCACTCGC<br>CACTGGAGCTGATGCCCTAGATACTCTGCAAGATGGCGACAAAGTGCTGATCAGTGAA<br>GCCTGTAGCCACAATGTACAGGAAGATGATATTGGCCGAGTTAAACTCCCCCGCTGGA<br>TCAATAGCTACACAGGCAAACAACTCGAGTTTGTAGTCACATCAGGACACGACTTTCC<br>CAATGATTTAGAGCAATATGCCCTCGTTATTCACTGCGGCGCTTGTATGTTTAATCGCA<br>ATGAAATGCTCCGTCGCATCCGCGAATGTCAACGGCGGCAGGTGCCCATCACTAACTA<br>TGGGGTGGCGATTTCTAAATTACAGGGCGTGCTGCCTCGAGTGCTTACGCCTTTTAACC<br>GCAATCCTCAGCAATAGTAAGTCTGGTAAAGAAACCGCTGCTGCGAAATTTGAACGCC<br>AGCACATGGACTCGTCTACTAGCGCAGCTTAATTAACCTAGGCTGCTGCCACCGCTGA<br>GCAATAACTAGCATAACCCCTTGGGGCCTCTAAACGGGTCTTGAGGGGTTTTTTGCTG<br>AAACCTCAGGCATTTGAGAAGCACACGGTCACACTGCTTCCGGTAGTCAATAAACCGG<br>TAAACCAGCAATAGACATAAGCGGCTATTTAACGACCCTGCCCTGAACCGACGACCGG<br>GTCATCGTGGCCGGATCTTGCGGCCCCTCGGCTTGAACGAATTGTTAGACATTATTTGC<br>CGACTACCTTGGTGATCTCGCCTTTCACGTAGTGGACAAATTCTTCCAACTGATCTGCG<br>CGCGAGGCCAAGCGATCTTCTTCTTGTCCAAGATAAGCCTGTCTAGCTTCAAGTATGAC<br>GGGCTGATACTGGGCCGGCAGGCGCTCCATTGCCCAGTCGGCAGCGACATCCTTCGGC<br>GCGATTTTGCCGGTTACTGCGCTGTACCAAATGCGGGACAACGTAAGCACTACATTTC<br>GCTCATCGCCAGCCCAGTCGGGCGGCGAGTTCCATAGCGTTAAGGTTTCATTTAGCGC<br>CTCAAATAGATCCTGTTCAGGAACCGGATCAAAGAGTTCCTCCGCCGCTGGACCTACC<br>AAGGCAACGCTATGTTCTCTTGCTTTTGTCAGCAAGATAGCCAGATCAATGTCGATCGT<br>GGCTGGCTCGAAGATACCTGCAAGAATGTCATTGCGCTGCCATTCTCCAAATTGCAGT<br>TCGCGCTTAGCTGGATAACGCCACGGAATGATGTCGTCGTGCACAACAATGGTGACTT<br>CTACAGCGCGGAGAATCTCGCTCTCTCCAGGGGAAGCCGAAGTTTCCAAAAGGTCGTT<br>GATCAAAGCTCGCCGCGTTGTTTCATCAAGCCTTACGGTCACCGTAACCAGCAAATCA<br>ATATCACTGTGTGGCTTCAGGCCGCCATCCACTGCGGAGCCGTACAAATGTACGGCCA<br>GCAACGTCGGTTCGAGATGGCGCTCGATGACGCCAACTACCTCTGATAGTTGAGTCGA<br>TACTTCGGCGATCACCGCTTCCCTCATACTCTTCCTTTTTCAATATTATTGAAGCATTTA<br>TCAGGGTTATTGTCTCATGAGCGGATACATATTTGAATGTATTTAGAAAAATAAACAA<br>ATAGCTAGCTCACTCGGTCGCTACGCTCCGGGCGTGAGACTGCGGCGGGCGCTGCGGA<br>CACATACAAAGTTACCCACAGATTCCGTGGATAAGCAGGGGACTAACATGTGAGGCA<br>AAACAGCAGGGCCGCGCCGGTGGCGTTTTTCCATAGGCTCCGCCCTCCTGCCAGAGTT<br>CACATAAACAGACGCTTTTCCGGTGCATCTGTGGGAGCCGTGAGGCTCAACCATGAAT<br>CTGACAGTACGGGCGAAACCCGACAGGACTTAAAGATCCCCACCGTTTCCGGCGGGTC

	GCTCCCTCTTGCGCTCTCCTGTTCCGACCCTGCCGTTTACCGGATACCTGTTCCGCCTTT<br>CTCCCTTACGGGAAGTGTGGCGCTTTCTCATAGCTCACACACTGGTATCTCGGCTCGGT<br>GTAGGTCGTTCGCTCCAAGCTGGGCTGTAAGCAAGAACTCCCCGTTCAGCCCGACTGC<br>TGCGCCTTATCCGGTAACTGTTCACTTGAGTCCAACCCGGAAAAGCACGGTAAAACGC<br>CACTGGCAGCAGCCATTGGTAACTGGGAGTTCGCAGAGGATTTGTTTAGCTAAACACG<br>CGGTTGCTCTTGAAGTGTGCGCCAAAGTCCGGCTACACTGGAAGGACAGATTTGGTTG<br>CTGTGCTCTGCGAAAGCCAGTTACCACGGTTAAGCAGTTCCCCAACTGACTTAACCTTC<br>GATCAAACCACCTCCCCAGGTGGTTTTTTCGTTTACAGGGCAAAAGATTACGCGCAGA<br>AAAAAAGGATCTCAAGAAGATCCTTTGATCTTTTCTACTGAACCGCTCTAGATTTCAGT<br>GCAATTTATCTCTTCAAATGTAGCACCTGAAGTCAGCCCCATACGATATAAGTTGTAAT<br>TCTCATGTTAGTCATGCCCCGCGCCCACCGGAAGGAGCTGACTGGGTTGAAGGCTCTC<br>AAGGGCATCGGTCGAGATCCCGGTGCCTAATGAGTGAGCTAACTTACATTAATTGCGT<br>TGCGCTCACTGCCCGCTTTCCAGTCGGGAAACCTGTCGTGCCAGCTGCATTAATGAATC<br>GGCCAACGCGCGGGGAGAGGCGGTTTGCGTATTGGGCGCCAGGGTGGTTTTTCTTTTC<br>ACCAGTGAGACGGGCAACAGCTGATTGCCCTTCACCGCCTGGCCCTGAGAGAGTTGCA<br>GCAAGCGGTCCACGCTGGTTTGCCCCAGCAGGCGAAAATCCTGTTTGATGGTGGTTAA<br>CGGCGGGATATAACATGAGCTGTCTTCGGTATCGTCGTATCCCACTACCGAGATGTCC<br>GCACCAACGCGCAGCCCGGACTCGGTAATGGCGCGCATTGCGCCCAGCGCCATCTGAT<br>CGTTGGCAACCAGCATCGCAGTGGGAACGATGCCCTCATTCAGCATTTGCATGGTTTG<br>TTGAAAACCGGACATGGCACTCCAGTCGCCTTCCCGTTCCGCTATCGGCTGAATTTGAT<br>TGCGAGTGAGATATTTATGCCAGCCAGCCAGACGCAGACGCGCCGAGACAGAACTTA<br>ATGGGCCCGCTAACAGCGCGATTTGCTGGTGACCCAATGCGACCAGATGCTCCACGCC<br>CAGTCGCGTACCGTCTTCATGGGAGAAAATAATACTGTTGATGGGTGTCTGGTCAGAG<br>ACATCAAGAAATAACGCCGGAACATTAGTGCAGGCAGCTTCCACAGCAATGGCATCCT<br>GGTCATCCAGCGGATAGTTAATGATCAGCCCACTGACGCGTTGCGCGAGAAGATTGTG<br>CACCGCCGCTTTACAGGCTTCGACGCCGCTTCGTTCTACCATCGACACCACCACGCTGG<br>CACCCAGTTGATCGGCGCGAGATTTAATCGCCGCGACAATTTGCGACGGCGCGTGCAG<br>GGCCAGACTGGAGGTGGCAACGCCAATCAGCAACGACTGTTTGCCCGCCAGTTGTTGT<br>GCCACGCGGTTGGGAATGTAATTCAGCTCCGCCATCGCCGCTTCCACTTTTTCCCGCGT<br>TTTCGCAGAAACGTGGCTGGCCTGGTTCACCACGCGGGAAACGGTCTGATAAGAGACA<br>CCGGCATACTCTGCGACATCGTATAACGTTACTGGTTTCACATTCACCACCCTGAATTG<br>ACTCTCTTCCGGGCGCTATCATGCCATACCGCGAAAGGTTTTGCGCCATTCGATGGTGT<br>CCGGGATCTCGACGCTCTCCCTTATGCGACTCCTGCATTAGGAAATTAATACGACTCAC<br>TATA

SUPPLEMENTARY REFERENCES

1. Stylianou, K. C.; Heck, R.; Chong, S. Y.; Bacsa, J.; Jones, J. T. A.; Khimyak, Y. Z.; Bradshaw, D.; Rosseinsky, M. J., A guest-responsive fluorescent 3D microporous metal-organic framework derived from a long-lifetime pyrene core. *J. Am. Chem. Soc.* **2010,** *132*, 4119–4130.

2. Gelderman, K. L., L.; Donne, S. W., Flat-Band Potential of a Semiconductor: Using the Mott–Schottky Equation. *J. Chem. Educ.* **2007,** *84*, 685-688.

3. Wan, C.; Zhou, L.; Sun, L.; Xu, L.; Cheng, D.-g.; Chen, F.; Zhan, X.; Yang, Y., Boosting visible-light-driven hydrogen evolution from formic acid over AgPd/2D g-C_3N_4 nanosheets Mott-Schottky photocatalyst. *Chem. Eng. J.* **2020,** *396*, 125229-125240.

4. Zhang, M.; Lu, M.; Lang, Z. L.; Liu, J.; Liu, M.; Chang, J. N.; Li, L. Y.; Shang, L. J.; Wang, M.; Li, S. L.; Lan, Y. Q., Semiconductor/Covalent-Organic-Framework Z-Scheme Heterojunctions for Artificial Photosynthesis. *Angew. Chem. Int. Ed.* **2020,** *59*, 6500-6506.

5. Sun, Y.; Lin, Y.; Harman, V. M.; Beynon, R. J.; Johnson, J. R.; Liu, L.-N., Decoding the Absolute Stoichiometric Composition and Structural Plasticity of a-Carboxysomes. *mBio* **2022,** *13*, e03629-21.

6. Shi, R.; Cao, Y.; Bao, Y.; Zhao, Y.; Waterhouse, G. I. N.; Fang, Z.; Wu, L. Z.; Tung, C. H.; Yin, Y.; Zhang, T., Self-Assembled Au/CdSe Nanocrystal Clusters for Plasmon-Mediated Photocatalytic Hydrogen Evolution. *Adv. Mater.* **2017,** *29*, 1-7.

7. Brown, K. A.; Wilker, M. B.; Boehm, M.; Dukovic, G.; King, P. W., Characterization of photochemical processes for H_2 production by CdS nanorod-[FeFe] hydrogenase complexes. *J. Am. Chem. Soc.* **2012,** *134*, 5627-5636.

8. Zadvornyy, O. A.; Lucon, J. E.; Gerlach, R.; Zorin, N. A.; Douglas, T.; Elgren, T. E.; Peters, J. W., Photo-induced H_2 production by [NiFe]-hydrogenase from *T. roseopersicina* covalently linked to a Ru(II) photosensitizer. *J. Inorg. Biochem.* **2012,** *106*, 151-155.

9. Caputo, C. A.; Gross, M. A.; Lau, V. W.; Cavazza, C.; Lotsch, B. V.; Reisner, E., Photocatalytic hydrogen production using polymeric carbon nitride with a hydrogenase and a bioinspired synthetic Ni catalyst. *Angew. Chem. Int. Ed.* **2014,** *53*, 11538-11542.

10. Caputo, C. A.; Wang, L.; Beranek, R.; Reisner, E., Carbon nitride-TiO_2 hybrid modified with hydrogenase for visible light driven hydrogen production. *Chem. Sci.* **2015,** *6*, 5690-5694.

11. Sakai, T.; Mersch, D.; Reisner, E., Photocatalytic hydrogen evolution with a hydrogenase in a mediator-free system under high levels of oxygen. *Angew. Chem. Int. Ed.* **2013,** *52*, 12313-12316.

12. Reisner, E.; Powell, D. J.; Cavazza, C.; Fontecilla-Camps, J. C.; Armstrong, F. A., Visible Light-Driven H_2 Production by Hydrogenases Attached to Dye-Sensitized TiO_2 Nanoparticles. *J. Am. Chem. Soc.* **2009,** *131*, 18457–18466.

13. Brown, K. A.; Dayal, S.; Ai, X.; Rumbles, G.; King, P. W., Controlled Assembly of Hydrogenase-CdTe Nanocrystal Hybrids for Solar Hydrogen Production. *J. Am. Chem. Soc.* **2010,** *132*, 9672–9680.

14. Hutton, G. A. M. M.; Reuillard, B.; Martindale, B. C. M. M.; Caputo, C. A.; Lockwood, C. W. J. J.; Butt, J. N.; Reisner, E., Carbon Dots as Versatile Photosensitizers for Solar-Driven Catalysis with Redox Enzymes. *J. Am. Chem. Soc.* **2016,** *138*, 16722-16730.

15. Holá, K.; Pavliuk, M. V.; Németh, B.; Huang, P.; Zdražil, L.; Land, H.; Berggren, G.; Tian, H., Carbon Dots and [FeFe] Hydrogenase Biohybrid Assemblies for Efficient Light-Driven Hydrogen Evolution. *ACS Catal.* **2020,** *10*, 9943-9952.
